# Interventions for the management of long covid (post-covid condition): living systematic review

**DOI:** 10.1136/bmj-2024-081318

**Published:** 2024-11-27

**Authors:** Dena Zeraatkar, Michael Ling, Sarah Kirsh, Tanvir Jassal, Mahnoor Shahab, Hamed Movahed, Jhalok Ronjan Talukdar, Alicia Walch, Samantha Chakraborty, Tari Turner, Lyn Turkstra, Roger S McIntyre, Ariel Izcovich, Lawrence Mbuagbaw, Thomas Agoritsas, Signe A Flottorp, Paul Garner, Tyler Pitre, Rachel J Couban, Jason W Busse

**Affiliations:** 1 Department of Anesthesia, McMaster University, Hamilton, ON, Canada; 2 Department of Health Research Methods, Evidence, and Impact, McMaster University, Hamilton, ON, Canada; 3 Faculty of Health Sciences, McMaster University, Hamilton, ON, Canada; 4 School of Public Health and Preventive Medicine, Monash University, Melbourne, Australia; 5 School of Rehabilitation Science and Program in Neuroscience, McMaster University, ON, Canada; 6 Department of Psychiatry and Pharmacology, University of Toronto, Toronto, ON, Canada; 7 Department of Medicine, Universidad del Salvador, Buenos Aires, Argentina; 8 Division General Internal Medicine, Department of Medicine, University Hospitals of Geneva, Geneva, Switzerland; 9 The MAGIC Evidence Ecosystem Foundation, Oslo, Norway; 10 Centre for Epidemic Interventions Research, Norwegian Institute of Public Health, Oslo, Norway; 11 Department of Clinical Sciences, Liverpool School of Tropical Medicine, Liverpool, UK; 12 Division of Respirology, Department of Medicine, University of Toronto, Toronto, ON, Canada

## Abstract

**Objective:**

To compare the effectiveness of interventions for the management of long covid (post-covid condition).

**Design:**

Living systematic review.

**Data sources:**

Medline, Embase, CINAHL, PsycInfo, Allied and Complementary Medicine Database, and Cochrane Central Register of Controlled Trials from inception to December 2023.

**Eligibility criteria:**

Trials that randomised adults (≥18 years) with long covid to drug or non-drug interventions, placebo or sham, or usual care.

**Results:**

24 trials with 3695 patients were eligible. Four trials (n=708 patients) investigated drug interventions, eight (n=985) physical activity or rehabilitation, three (n=314) behavioural, four (n=794) dietary, four (n=309) medical devices and technologies, and one (n=585) a combination of physical exercise and mental health rehabilitation. Moderate certainty evidence suggested that, compared with usual care, an online programme of cognitive behavioural therapy (CBT) probably reduces fatigue (mean difference −8.4, 95% confidence interval (CI) −13.11 to −3.69; Checklist for Individual Strength fatigue subscale; range 8-56, higher scores indicate greater impairment) and probably improves concentration (mean difference −5.2, −7.97 to −2.43; Checklist for Individual Strength concentration problems subscale; range 4-28; higher scores indicate greater impairment). Moderate certainty evidence suggested that, compared with usual care, an online, supervised, combined physical and mental health rehabilitation programme probably leads to improvement in overall health, with an estimated 161 more patients per 1000 (95% CI 61 more to 292 more) experiencing meaningful improvement or recovery, probably reduces symptoms of depression (mean difference −1.50, −2.41 to −0.59; Hospital Anxiety and Depression Scale depression subscale; range 0-21; higher scores indicate greater impairment), and probably improves quality of life (0.04, 95% CI 0.00 to 0.08; Patient-Reported Outcomes Measurement Information System 29+2 Profile; range −0.022-1; higher scores indicate less impairment). Moderate certainty evidence suggested that intermittent aerobic exercise 3-5 times weekly for 4-6 weeks probably improves physical function compared with continuous exercise (mean difference 3.8, 1.12 to 6.48; SF-36 physical component summary score; range 0-100; higher scores indicate less impairment). No compelling evidence was found to support the effectiveness of other interventions, including, among others, vortioxetine, leronlimab, combined probiotics-prebiotics, coenzyme Q10, amygdala and insula retraining, combined L-arginine and vitamin C, inspiratory muscle training, transcranial direct current stimulation, hyperbaric oxygen, a mobile application providing education on long covid.

**Conclusion:**

Moderate certainty evidence suggests that CBT and physical and mental health rehabilitation probably improve symptoms of long covid.

**Systematic review registration:**

Open Science Framework https://osf.io/9h7zm/.

**Readers’ note:**

This article is a living systematic review that will be updated to reflect emerging evidence. Updates may occur for up to two years from the date of original publication.

## Introduction

The covid-19 pandemic has affected hundreds of millions of people worldwide, with major consequences for health and economies.^1^ ^2^ Although most patients recover, evidence suggests that as many as 15% might experience long term health effects from covid-19, including fatigue, myalgia, and impaired cognitive function, called post-covid condition, or long covid.^3^ ^4^ ^5^ ^6^ ^7^ ^8^ ^9^ ^10^ ^11^ ^12^ The prevalence of long covid is difficult to establish because most symptoms are non-specific, and many studies lack sufficiently rigorous designs to confidently attribute symptoms to covid-19 infection.^13^ ^14^ Estimates suggest that at least 65 million people globally experience symptoms that impair their functional and cognitive capacity.^15^ ^16^

The pathophysiology of long covid is uncertain, and investigators have proposed several potential causes, including viral persistence, autoimmunity, “micro-clots,” and psychological mechanisms.^17^ Moreover, the definition of long covid is heterogeneous and might comprise several distinct phenotypes.^18^

Risk factors for the development of long covid include female sex, greater comorbidity, and patient reported psychological distress.^19^ ^20^ ^21^ Conversely, severity of acute covid-19 infection may not predict long covid, and even patients with mild infections appear to be susceptible.^22^ Symptoms of long covid may persist after acute infection, or they may relapse and remit.^23^ Evidence on the trajectory of long covid is limited, but some studies suggest that many patients experience a reduction in symptoms at one year after acute infection.^24^ ^25^ Also, research into the burden of long covid in low and middle income countries is scarce.^16^ ^26^ Evidence suggests that patients in these countries currently receive fragmented care, owing to constraints on health resources and competing priorities.^16^

Considerable resources have been invested to study long covid, including $1bn (£0.8bn; €0.9bn) from the US National Institutes of Health (NIH).^27^ Several trials testing interventions for the management of long covid have been published to date,^28^ ^29^ ^30^ ^31^ and hundreds more are planned or are ongoing.^32^ ^33^ ^34^ ^35^ ^36^ However, these trials will be published faster than evidence users, such as clinicians and patients, can read or interpret them; they could produce conflicting results; and will come with strengths and limitations that might not be immediately apparent.

Healthcare providers are increasingly encountering patients with long covid, and, in the absence of trustworthy and up-to-date summaries of the evidence, patients may receive unproven, costly, and harmful treatments.^37^ ^38^ ^39^ ^40^ ^41^ ^42^ Some patients and healthcare providers have questioned the credibility of interventions in published trials, such as exercise and cognitive behavioural therapy (CBT).^43^ ^44^ ^45^ Trustworthy systematic reviews that clarify the benefits and harms of available interventions are critical to promote evidence based care. Therefore, we present the first iteration of a living systematic review of interventions for the management of long covid.

## Methods

We submitted our review protocol to MedRxiv in March 2024.^46^

### Eligibility criteria

Eligible studies enrolled adults (≥18 years) with long covid—defined by the World Health Organization (WHO) as symptoms at ≥3 months after laboratory confirmed, probable, or suspected covid-19 infection that persisted for at least two months—and randomised them either to any drug or non-drug intervention, placebo or sham, usual care, or to alternative drug or non-drug interventions, without any restrictions on date or language of publication.^23^ This definition, although broad, is consistent with the most recent definition published by the National Academies of Sciences, Engineering, and Medicine and reflects the limitations in current scientific knowledge about long covid.^47^ ^48^ Based on empirical evidence showing that preprints and published reports of randomised trials generally provide consistent results, we included both preprint and published trial reports.^49^ ^50^ ^51^ ^52^

We planned to conduct sensitivity analyses excluding trials that did not report the time since acute covid-19 infection or the duration of long covid symptoms according to WHO criteria. It was not possible to perform these analyses, however, owing to the limited number of trials addressing each class of intervention and outcome.

We excluded trials if ≥20% of patients had recovered from covid-19 less than three months before randomisation; pseudorandomised trials; trials of animals; and trials investigating treatments for acute covid-19 or interventions to prevent long covid.^23^ ^53^ Trials were also excluded that targeted patients experiencing only anosmia and hyposmia after covid-19 infection, as these patients likely form a group that is distinct from those with other typical symptoms of long covid (eg, fatigue, pain, shortness of breath, cognitive impairment). Additionally, we excluded randomised trials with fewer than 25 participants in each arm. Smaller trials are unlikely to meaningfully contribute to meta-analyses, more likely to include unrepresentative samples and arms that are prognostically imbalanced, and at higher risk of publication bias.^54^

### Search strategy

We worked with an experienced research librarian to search Medline, Embase, Cochrane Central Register of Controlled Trials, PsycInfo, Allied and Complementary Medicine Database, and CINAHL from inception to December 2023 (see supplement 1). Our search combined terms related to long covid with a filter for randomised trials. In February 2024, we supplemented our search using the Epistemonikos covid-19 Repository—a living catalogue of covid-19 research—and by reviewing the references of relevant systematic reviews and soliciting experts for eligible trials.^30^ ^33^ ^55^ ^56^

### Study selection

Following training and calibration exercises to ensure sufficient agreement, pairs of reviewers worked independently and in duplicate to screen the titles and abstracts of search records and subsequently the full texts of articles considered potentially eligible. We used the online systematic review software Covidence (https://www.covidence.org) to assist with screening. Reviewers resolved disagreements by discussion, or, if necessary, adjudication by a third reviewer.

### Data extraction

Following training and calibration exercises to ensure sufficient agreement, pairs of reviewers worked independently and in duplicate to collect data from eligible trials using a pilot tested Excel spreadsheet (Microsoft Office Excel 2019). Reviewers resolved disagreements by discussion or by consultation with a third reviewer. A third experienced reviewer checked all consensus data to confirm accuracy.

Reviewers collected data on trial characteristics (eg, trial design, country of origin, funding sources, diagnostic criteria for long covid), patient characteristics (eg, age, sex, employment and education status, receipt of covid-19 vaccination, method of acute covid-19 diagnosis, severity of acute covid-19 infection, duration of long covid symptoms, number of covid-19 infections, long covid symptoms), characteristics of interventions and comparators (eg, type of intervention, treatment duration), and patient important outcomes. Our outcomes of interest were informed by a published core outcome set for long covid^57^ ^58^ and discussions with patient partners and clinicians. We included fatigue, pain, post-exertional malaise, changes in education or employment status, cognitive function, mental health, dyspnoea, quality of life, patient reported physical function, recovery or improvement, and serious adverse events (as defined by each trial).^57^ ^58^ We extracted data for all instruments used in trials that measured any of our outcomes of interest.

For dichotomous outcomes, reviewers extracted the number of patients and events in each arm, and, for continuous outcomes, the number of patients, a measure of central tendency (mean or median), and a measure of variability (eg, standard deviation, standard error, 95% confidence interval, P value). For continuous outcomes, reviewers prioritised extracting changes in the outcome measure from baseline, and, if not reported, the outcome measure at follow-up.

For each outcome, reviewers preferentially extracted the results from intention-to-treat analyses without imputation for missing data. We extracted results immediately after the end of the intervention and at the longest reported point of follow-up at which randomisation was maintained. Given the relapsing and remitting nature of long covid and the potential for interventions to have long term effects, for crossover trials we only collected data for the first phase of the trial before washout and crossover of patients.

Long covid can comprise several distinct phenotypes, and we anticipated that the effects of interventions might differ based on the predominant symptoms patients experience. Accordingly, based on previous classifications of long covid and the eligibility criteria of trials,^18^ ^59^ ^60^ we categorised trials as including patients with either general symptoms such as fatigability and an impairment in functional capacity to perform routine activities of daily living, primarily respiratory sequelae characterized by dyspnoea, or primarily neurological or cognitive sequelae characterized by cognitive impairments and brain fog.

We also anticipated that the effects of interventions might depend on diagnostic criteria for long covid, severity of acute covid-19 infection, time since infection, number of infections, vaccination status, and SARS-CoV-2 variant.^19^ When reported, we extracted stratified data based on these factors for subgroup analyses.

In response to growing concerns about untrustworthy trial publications,^61^ ^62^ reviewers applied the trustworthiness in randomised controlled trials (TRACT) checklist to assess each trial for signs of data fabrication, data falsification (manipulation of data or results), and errors in the conduct of the trial or analysis of data that could undermine the conclusions, such as confusing standard errors with standard deviations and misclassification of intervention and control groups.^63^ This checklist includes 19 items in seven domains: governance, author group, plausibility of intervention, timeframe, dropouts, baseline characteristics, and outcomes. The checklist does not include a cut-off at which a trial is considered suspicious, and experience in applying the checklist to systematic reviews is currently limited. Therefore, the core authorship group reviewed all trials flagged as having potential concerns in one or more domain and identified those they considered untrustworthy by consensus.

### Risk of bias assessments

Following training and calibration, reviewers worked independently and in duplicate to assess risk of bias of eligible trials using a modified version of the Cochrane endorsed risk of bias 2.0 tool.^64^ This instrument assesses the risk of bias across five domains: bias due to randomisation, bias due to deviations from the intended intervention, bias due to missing outcome data, bias due to measurement of the outcome, and selective outcome reporting.

The risk of bias 2.0 tool necessitates that reviewers distinguish between whether they are interested in the effect of assignment or adherence to the intervention. We assessed the risk of bias of the effect of assignment rather than adherence to the intervention because this effect is likely to be observed in clinical settings.

Our modified version of the tool includes the same domains as the original risk of bias 2.0 tool, but with revised response options (ie, definitely low risk of bias, probably low risk of bias, probably high risk of bias, and definitely high risk of bias) and guidance tailored to issues relevant for the present review. Specifically, we removed guidance for assessing risk of bias of adhering to the intervention and listed important cointerventions that may be imbalanced between trial arms for consideration in making judgements about deviations from the intended intervention (eg, activity management, physical activity, social engagement).

We considered trials without blinding of patients, healthcare providers, and investigators at high risk of bias owing to deviations from intended intervention and measurement of outcome. An exception was made for trials that compared two or more interventions that were matched for level of interaction between trial participants and healthcare providers.^65^ ^66^ Patients might expect interventions with higher levels of interaction to be more effective, potentially influencing their perception of outcomes and their likelihood of pursuing additional beneficial activities. When interventions are matched for interaction, patients are less likely to have strong preconceptions about their comparative effectiveness.

Information reported in published trial protocols or trial registrations formed the basis of our judgements about selective reporting. Reviewers resolved disagreements by discussion, or consultation with a third reviewer when necessary.

### Data synthesis and analysis

We used descriptive characteristics to describe trials and participants. Means, medians, and associated measures of variability (eg, 95% confidence intervals (CIs), interquartile ranges (IQRs)) were used for continuous variables, whereas counts and proportions were used for dichotomous and categorical variables.

Although we intended to perform network meta-analyses to summarise the comparative efficacy and harms of interventions, the available evidence was too sparse. In situations in which network meta-analysis is not possible, we had planned to perform frequentist random effects pairwise meta-analyses with the restricted maximum likelihood heterogeneity estimator.^67^ ^68^ Overall, the diversity in interventions and outcome measures precluded meta-analyses. Therefore, for most comparisons and outcome measures, we describe the results of individual trials.

The number of participants and events were used to calculate relative risks for dichotomous outcomes, except for serious adverse events, when risk differences were calculated owing to the propensity for trials to report 0 events for control arms. We used the number of participants and mean change or mean end scores to calculate mean differences for continuous outcomes.^69^ Based on evidence suggesting that the two methods are comparable for randomised trials, we did not calculate mean change scores from baseline for trials that reported outcome measures at end of follow-up.^69^

To enhance interpretation, reviewers may convert effects measured by different instruments assessing the same construct into a commonly used or familiar instrument.^70^ ^71^ We avoided converting effects across instruments owing to potential differences in the range of constructs covered by each instrument. We also avoided standardised mean differences as they can be influenced by differences in variability across trial populations.^71^ Although we intended to test for small study effects for analyses that included ≥10 trials, too few trials were available across all comparisons.^72^ ^73^

To enhance interpretability of results, we transformed relative risks to absolute effects (number of patients with the outcome per 1000 patients), using the control group event rate as the baseline risk.^74^ We performed all analyses using the *meta* package, version 4.1.2, in R (Vienna, Austria). All data and code to reproduce our results are freely accessible on Open Science Framework (https://osf.io/9h7zm/).

### Subgroup and sensitivity analysis

To explain potential heterogeneity in results across trials, we generated seven a priori factors: diagnostic criteria for long covid, time since infection, number of infections, vaccination status, severity of acute covid-19, SARS-CoV-2 variant, and predominant symptoms experienced by patients .^19^ ^75^ We also intended to avoid indiscriminately pooling trials rated at low and high risk of bias. We planned to test for differences between the results of trials at low and high risk of bias, and, if important differences were detected, to rely only on trials at low risk of bias. However, we did not identify sufficient evidence to perform any subgroup analyses. As more evidence accumulates from trials, we intend to perform future subgroup analyses to investigate these factors.

### Certainty of evidence

We used the Grading of Recommendations Assessment, Development and Evaluation (GRADE) approach to assess the certainty (quality) of evidence.^76^ This approach rates the certainty of evidence as high, moderate, low, or very low certainty based on considerations of risk of bias (study limitations), inconsistency (heterogeneity in results across trials), indirectness (differences between questions addressed in studies and the question of interest), publication bias (tendency for studies with positive results to be published, published faster, or published in journals with higher visibility), and imprecision (random error). High or moderate certainty evidence indicates confidence that the estimated effect represents the true effect, and low or very low certainty evidence indicates the estimated effect may be substantially different from the true effect.

To enable imprecision to be judged, we considered whether effect estimates met or exceeded the minimal important difference (MID)—the smallest difference in an outcome that patients find important.^77^ When the point estimate met or exceeded the MID, we rated the certainty of there being an important effect. Conversely, when the point estimate was between the MID and the null, we rated the certainty of there being no important effect. We anticipate that decision makers will further contextualise our judgements about the certainty of evidence to make decisions or formulate guideline recommendations.^78^ ^79^

After discussion with coauthors and patient partners, we considered a risk difference of 50 per 1000 patients as the MID for the outcome of important improvement and recovery, and a risk difference of 20 per 10000 patients as minimally important for serious adverse events. To source MIDs for other patient reported outcomes from published studies, we performed pragmatic searches of Google Scholar using terms related to MIDs and the measure of interest.

MIDs of patient reported outcomes are determined using either anchor based methods or distribution based methods.^80^ Anchor based methods rely on an external “anchor” to interpret the magnitude of change in a measure or outcome. Conversely, distribution based methods rely on the distribution of the data to interpret the importance of change in a measure. We prioritised anchor based MID estimates over distribution based MID estimates, because they better reflect patients’ direct experiences.^81^ ^82^

The MID of an instrument depends on the patient’s condition and the intervention being studied.^83^ We were unable to identify any MIDs specific to long covid. Instead, we prioritised MIDs for patients with other chronic health conditions. When it was not possible to identify an MID, we used distribution based MIDs, defined as 0.5 standard deviations of the measure at baseline.^84^ When several candidate MIDs or a range of MIDs were identified, we used the median MID or the MID we considered most trustworthy according to established criteria.^85^ Supplement 2 lists MIDs that guided our judgements related to imprecision.

### Reporting

We report our systematic review according to the PRISMA (preferred reporting items for systematic reviews and meta-analyses) checklist.^86^ PRISMA flow diagrams illustrate the total number of search records, the number of records excluded, reasons for exclusion, and the total number of trials included in our review. GRADE Evidence Profiles summarise effect estimates and the associated certainty of evidence for each intervention.^74^

We describe our results using GRADE plain language summaries—that is, describing high certainty evidence with declarative statements, moderate certainty evidence with “probably,” low certainty with “may,” and very low with “very uncertain.”^87^ In reporting results, we focus primarily on interventions with moderate to high certainty evidence.

### Patient and public involvement

The Long Covid Web Patient Advisory Council (https://www.longcovidweb.ca/) reviewed and offered feedback on our protocol. Furthermore, we engaged an individual with lived experience as a member of our study team, who provided feedback on our protocol and interpretation of findings. Patient perspectives guided the prioritisation of outcomes, the selection of MIDs, the interpretation of evidence, and the development of clear, easily understandable ways to communicate results.

## Results

### Study and patient characteristics

**Fig 1:**
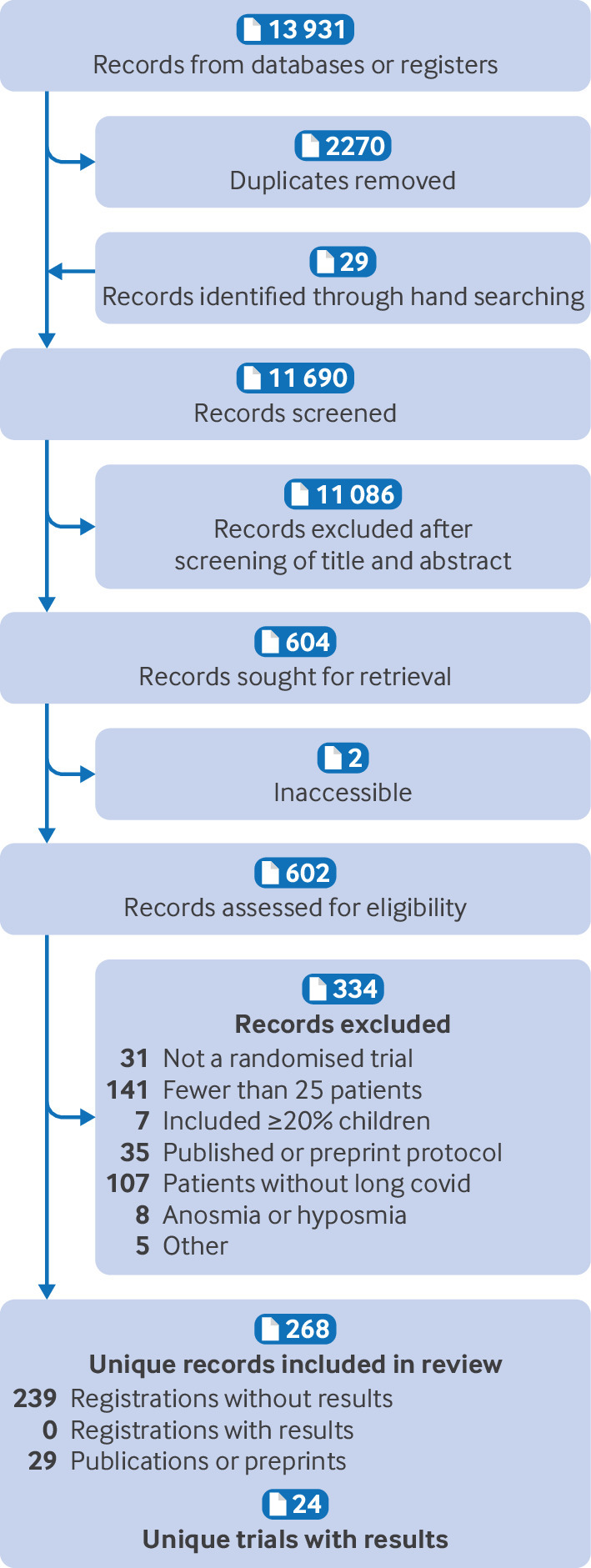
Selection of trials for inclusion in systematic review

Overall, we identified 24 unique trials with 3695 patients. We also identified 239 registered trials that were ongoing or had been completed but the results not yet published (fig 1).

Four trials (n=708 patients) investigated drug interventions,^88^ ^89^ ^90^ ^91^ eight (n=985) physical activity or rehabilitation,^92^ ^93^ ^94^ ^95^ ^96^ ^97^ ^98^ ^99^ three (n=314) behavioural,^100^ ^101^ ^102^ four (n=794) dietary,^28^ ^103^ ^104^ ^105^ four (n=309) medical devices and technologies,^106^ ^107^ ^108^ ^109^ and one (n=585) a combination of physical exercise and mental health rehabilitation.^110^

Two trials engaged patients in the design of the intervention and trial protocol.^100^ ^110^ All trials were available as publications in peer reviewed journals or were deposited as preprints and subsequently published in peer reviewed journals. Trials were predominantly conducted in the Americas or Europe, with funding from government sources or no funding, and they were typically published in either 2022 or 2023. The median number of patients randomised among trials was 100 (IQR 60-153), and outcomes were reported at up to one year of follow-up.

Two trials (n=523 patients) reported on patients with neurological or cognitive symptoms,^89^ ^108^ and three trials (n=401) on patients with respiratory symptoms.^92^ ^93^ ^97^ The remaining 19 trials reported on patients with general symptoms of long covid. These symptoms included fatigue,^96^ ^100^ ^105^ ^106^ ^107^ reduced functional capacity,^94^ ^100^ ^106^ and one or more of a range of different symptoms typically including general fatigue and lethargy.^88^ ^103^ ^104^ Seven trials did not report the specific symptoms experienced by patients.^28^ ^90^ ^91^ ^98^ ^99^ ^101^ ^102^ ^111^

More than half of patients had a reported history of laboratory confirmed SARS-CoV-2 infection, and about a third were admitted to hospital with severe covid-19. Vaccination status was only reported in three trials, in which most patients were fully vaccinated^28^ ^100^ ^103^ (table 1, also see supplement 3).

**Table 1:** Characteristics of randomised trials included in review. Values represent number (percentage) of trials or percentage (number) of patients included in trials

Characteristics	Estimates
Journal publication type:	
Peer reviewed	22 (91.7)
Preprint and peer reviewed	2 (8.3)
Trial design:	
Parallel	23 (95.8)
Crossover	1 (4.2)
WHO region:	
Americas	5 (20.8)
Eastern Mediterranean	3 (12.5)
Europe	15 (62.5)
Western Pacific	1 (4.2)
Registered	16 (66.7)
Funding*:	
Industry	4 (16.7)
Government	6 (25)
Institutional	5 (20.8)
Not-for-profit	2 (8.3)
None	5 (20.8)
Not reported	5 (20.8)
Year of publication:	
2021	1 (4.2)
2022	7 (29.2)
2023	15 (62.5)
2024	1 (4.2)
Method of recruitment*:	
Specialised long covid outpatient clinic	3 (12.5)
General practitioner	1 (4.2)
Social or traditional media	3 (12.5)
Other	13 (54.2)
Not reported	7 (29.2)
Median (IQR) duration of follow-up (weeks)	8 (4.8-15.6)
Subtype of long covid:	
General	19 (79.2)
Respiratory	3 (12.5)
Neurological or cognitive	2 (8.3)
Method of acute covid-19 diagnosis:	
Laboratory confirmed PCR, antigen, or antibody test	49.5 (n=1828)
At home antigen test	0 (n=0)
Doctor diagnosed	0.8 (n=31)
Not reported	49.7 (n=1836)
Severity of acute covid-19:	
Hospital admission	40 (n=1477)
ICU admission	8.6 (n=316)
Not reported	36.8 (n=1361)
Proportion of male participants across all trials	35 (n=1292)
Age weighted mean	51.1
Median (IQR) No of participants	100 (60-153)
Types of interventions:	
Drug	4 (16.7)
Physical activity and rehabilitation	8 (33.3)
Behavioural	3 (12.5)
Diet or dietary supplement	4 (16.7)
Medical devices and technologies	4 (16.7)
Combination treatments	1 (4.2)

ICU=intensive care unit; IQR=interquartile range; PCR=polymerase chain reaction; WHO=World Health Organization.

* Trials could be classified into more than one category.

We identified six trials (25%) with concerns about the integrity of the results or trial execution.^89^ ^92^ ^95^ ^96^ ^104^ ^106^ These problems included retrospective trial registration; improbably large benefits; unusually small variability in baseline characteristics or outcome data, or both; and highly similar trial arms that were unlikely considering differences that could arise naturally through randomisation.

### Risk of bias

**Fig 2:**
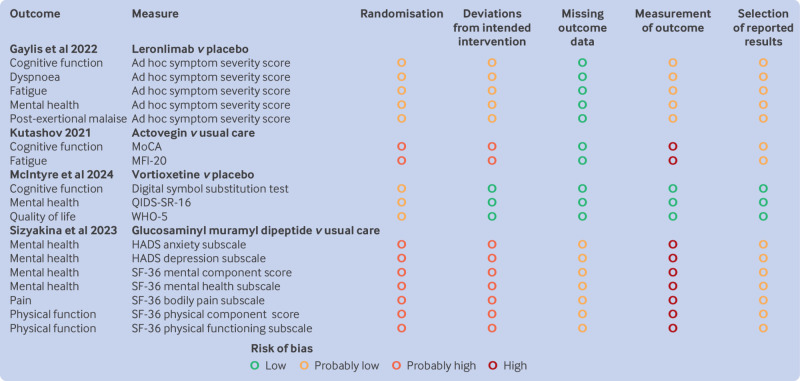
Risk of bias of trials reporting on drug interventions for symptoms of long covid. HADS=Hospital Anxiety and Depression Scale; MFI-20=Multidimensional Fatigue Inventory-20; MoCA=Montreal Cognitive Assessment test; SF-36=short form-36; QIDS-SR-16=Quick Inventory of Depressive Symptomatology-16-item; WHO-5=World Health Organization-5 wellbeing index

Figure 2 presents the risk of bias for trials that reported on drug interventions. Supplements 4-9 present the risk of bias of non-drug interventions. About half of all results were rated at high risk of bias, primarily because of concerns about imbalances in potential co-interventions and expectancy effects due to lack of blinding and comparisons with control interventions not matched for degree of interaction between patients and healthcare providers.

### Summary of findings

**Fig 3:**
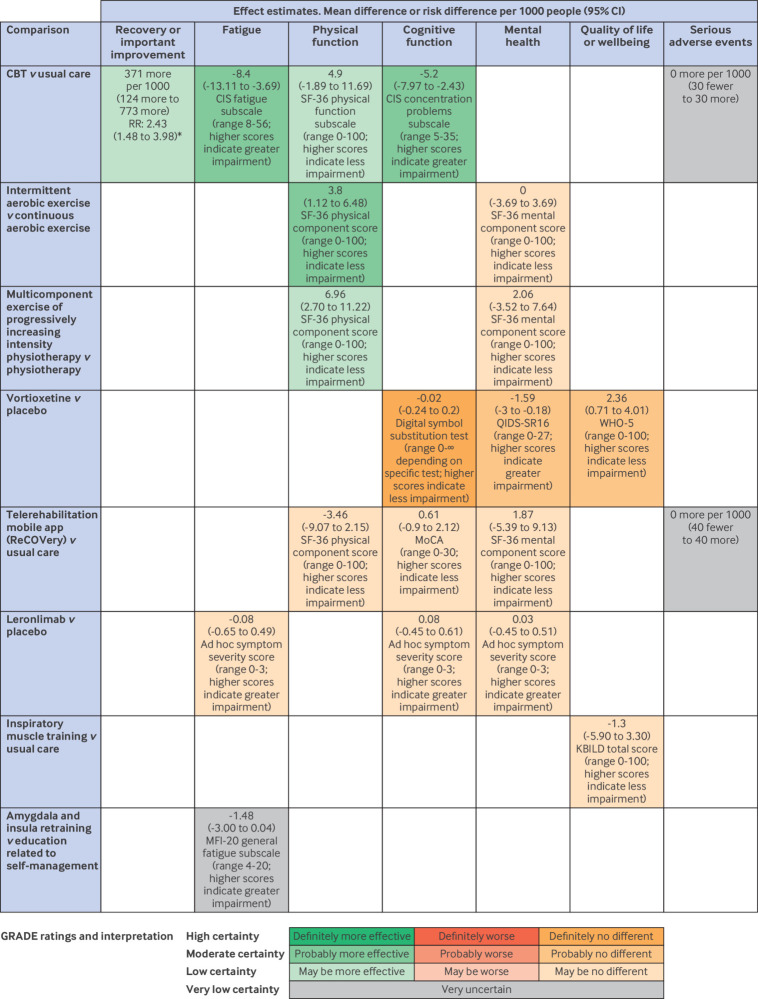
Effects of drug interventions, physical activity and rehabilitation, and behavioural interventions on symptoms of long covid. *Classified as no longer severely fatigued according to CIS fatigue subscale (score <35). CBT=cognitive behavioural therapy; CI=confidence interval; CIS=Checklist for Individual Strength; KBILD=King’s Brief Interstitial Lung Disease; MFI-20=Multidimensional Fatigue Inventory-20; MoCA=Montreal Cognitive Assessment test; SF-36=short form-36; QIDS-SR-16=Quick Inventory of Depressive Symptomatology16-item; RR=relative risk; WHO-5=World Health Organization-5 wellbeing index

**Fig 4:**
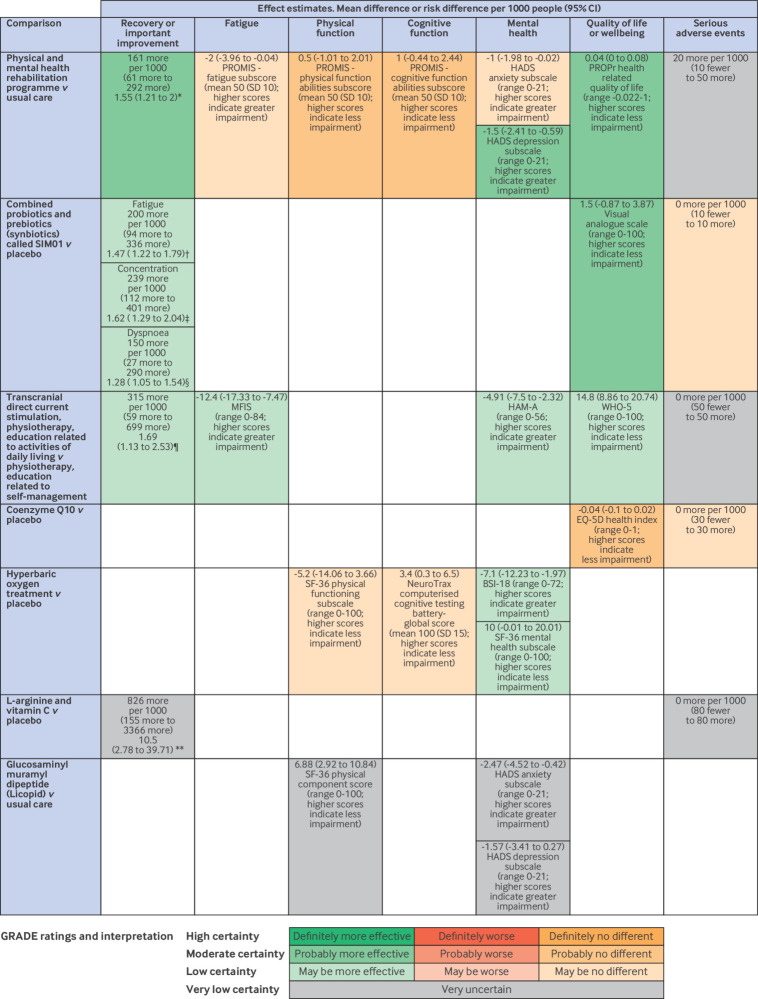
Effects of dietary interventions, medical devices and technologies, and combined interventions on symptoms of long covid. Effect estimates are mean difference or risk difference per 1000 people (95% CI). *Overall health compared with three months previously. Effect estimates are mean difference or risk difference per 1000 people (95% CI). Those who reported being “much better now” or “somewhat better now” were classified as having improved. †Reduction in severity of fatigue leading to improvement in activities of daily living using PACSQ-14 questionnaire. ‡Alleviation of difficulty in concentration leading to improvement in activities of daily living using PACSQ-14 questionnaire. §Alleviation of shortness of breath leading to improvement in activities of daily living using PACSQ-14 questionnaire. ¶Five point reduction in MFIS (range 0-84, with higher scores indicating greater impairment). **Fatigue was operationalised as the response “most or all the time” to item 7 of the Center for Epidemiological Studies Depression Scale (“I felt that everything I did was an effort”). CI=confidence interval; BSI-18=Brief Symptom Inventory-18; EQ-5D=European quality of life-5 dimensions; GRADE=Grading of Recommendations Assessment, Development and Evaluation; HADS=Hospital Anxiety and Depression Scale; HAM-A=Hamilton Anxiety Rating scale; MFIS=Modified Fatigue Impact Scale; PACSQ-14=post-acute covid-19 syndrome questionnaire; PROMIS=Patient-Reported Outcomes Measurement Information System; PROPr=PROMIS 29+2 Profile version 2.1; RR=relative risk; WHO-5=World Health Organization-5 wellbeing index

Figure 3 presents the summary of findings of drug, physical activity and rehabilitation, and behavioural interventions. Figure 4 presents the summary of findings of dietary interventions and supplements, medical devices and technologies, and combination treatments.

#### Drug interventions

Four trials (708 patients) investigated drug interventions for general symptoms of long covid.^88^ ^89^ ^90^ ^91^ Only one of these trials reported on vortioxetine, a Food and Drug Administration approved drug.^90^ Other drugs investigated included leronlimab (a monoclonal antibody that binds to C-C chemokine receptor 5),^88^ ^112^ ^113^ glucosaminyl muramyl dipeptide (called Licopid),^91^ and actovegin (derived from ultrafiltered calf blood).^89^ Supplements 10-13 present GRADE summary of findings tables for drug interventions.

High certainty evidence shows that vortioxetine treatment for eight weeks does not improve cognitive function, and moderate certainty evidence suggests that vortioxetine probably has little or no effect on depressive symptoms and quality of life.

Other interventions were supported by low or very low certainty evidence or by trials with issues that raised concerns about their integrity.

#### Physical activity and rehabilitation interventions

Eight trials (n=985 patients) investigated physical activity or rehabilitation interventions.^92^ ^93^ ^94^ ^95^ ^96^ ^97^ ^98^ ^99^ Supplements 14-19 present GRADE summary of findings tables.

Two trials (n=209 patients) compared rehabilitation programmes involving physical activity against usual care or general education about covid-19 and activities of daily living.^98^ ^99^ Physical activity programmes involved two or three 60 minute exercise sessions incorporating aerobic exercise and strength training for 12 or 15 weeks, one of which was delivered online.^98^ ^99^ These trials did not report on our outcomes of interest.^98^ ^99^

Moderate certainty evidence from one trial (n=110 patients) suggests that intermittent aerobic exercise 3-5 times weekly for 4-6 weeks probably improves physical function compared with continuous exercise (mean difference 3.8, 95% CI 1.12 to 6.48); SF-36 physical component score; range 0-100; higher scores indicate less impairment).^94^

Other trials compared a programme of multicomponent exercise of increasing intensity combined with physiotherapy against physiotherapy alone,^97^ low versus high intensity aerobic and strength training,^95^ a programme of in-patient rehabilitation combined with acupuncture against in-patient rehabilitation alone,^96^ inspiratory muscle training (a form of respiratory training to strengthen the muscles involved in inhalation) against usual care,^93^ and a combination of physiotherapy and active cycle of breathing (breathing exercises intended to improve dyspnoea) against physiotherapy alone.^92^ The effects of these interventions were supported by only low or very low certainty evidence.

#### Behavioural interventions

Three trials (n=314 patients) investigated behavioural interventions.^100^ ^101^ ^102^ ^111^ Supplements 20-22 present GRADE summary of findings tables.

One trial (n=114 patients) of general long covid symptoms, compared a 17 week online CBT programme called “fit after covid” versus usual care. The programme was developed based on existing CBT protocols for severe fatigue in long term medical conditions, with the option for trained psychologists to deliver the programme in-person for those who were unable or unwilling to use the internet based format.^100^ The programme addressed disruptive sleep-wake patterns, unhelpful beliefs about fatigue, low activity level, social support, fears and worries about covid-19, and poor pain coping mechanisms.^100^

Moderate certainty evidence suggested that CBT probably reduces fatigue (mean difference −8.4, 95% CI −13.11 to −3.69; Checklist for Individual Strength fatigue subscale; range 8-56; higher scores indicate greater impairment) and probably improves concentration (mean difference −5.2, −7.97 to −2.43; Checklist for Individual Strength concentration problems subscale; range 5-35; higher scores indicate greater impairment).

Other trials investigated an educational mobile application, called ReCOVery, that included modules advising patients on diet, sleep, and exercise^101^ ^111^ and amygdala and insula retraining—a programme involving neuroplasticity, mindfulness based meditation, alternate nostril breathing, and other lifestyle related treatments.^102^ These interventions were supported by only low or very low certainty evidence.

#### Dietary supplements and other dietary interventions

Four trials (n=794 patients) investigated dietary supplements.^28^ ^103^ ^104^ ^105^ These trials investigated a formulation of probiotics and prebiotics (synbiotics) called SIM01,^103^ coenzyme Q10,^28^ L-arginine and liposomal vitamin C,^105^ and a combination of trimethyl hydrazinium propionate and ethyl methyl hydroxy pyridine succinate (Brainmax)^104^ against placebo. Supplements 23-26 present GRADE summary of findings tables.

According to low certainty evidence from one trial (n=463 patients), a formulation of synbiotics (SIM01) might alleviate fatigue (200 more per 1000 patients, 95% CI 94 more to 336 more), improve concentration (239 more per 1000 patients, 112 more to 401 more), and improve dyspnoea (150 more per 1000 patients, 27 more to 290 more). Moderate certainty evidence, however, suggested that SIM01 probably does not improve quality of life.^103^

We judged results for alleviation of symptoms in the trial addressing the effects of SIM01 to be at high risk of bias due to selective reporting.^103^ Although early versions of the trial registration include long covid symptoms as a secondary outcome, the methods and criteria for ascertaining alleviation of these symptoms were not described.^103^ After the trial concluded, the trial registration was modified to include additional details on methods for ascertaining symptom alleviation, and this outcome was reclassified as the primary outcome.^103^ We also rated down the certainty of evidence as the trial reported a large effect on fatigue, concentration, and dyspnoea, and other symptoms such as hair loss, for which there is no plausible mechanism of action. Furthermore, this formulation of synbiotics, SIM01, has not been independently tested or shown to be effective for long covid or other conditions, except by its named innovators and patent holders.

Moderate certainty evidence from one trial (n=119 patients) suggests that coenzyme Q10, administered at 500 mg/day for six weeks, probably does not improve quality of life.

Other interventions were supported by only low or very low certainty evidence or by trials with concerns about their integrity.

#### Medical devices and technologies

Four trials (n=309 patients) investigated medical devices and technologies, including hyperbaric oxygen, active high definition transcranial direct current stimulation, photobiomodulation, and active hydrogen therapy.^106^ ^107^ ^108^ ^109^ Supplements 27-29 present GRADE summary of findings tables.

All interventions were supported only by low or very low certainty evidence, or by trials with concerns about their integrity.

#### Combination treatments

One trial (n=585 patients) in patients with general long covid symptoms and a history of severe covid-19, evaluated a combined physical and mental health rehabilitation programme versus usual care (single session of online advice and support).^110^ This intervention was delivered online over eight weeks by exercise physiologists, physiotherapists, and health psychologists and consisted of weekly live, supervised, group exercise and psychological support sessions that focused on motivation, fear avoidance, managing emotions, fatigue, and stress and anxiety.^110^ Supplement 30 presents the GRADE summary of findings table.

Moderate certainty evidence suggested that a combined programme of physical and mental health rehabilitation probably increases the proportion of patients who experience recovery or important improvements (161 more per 1000 patients, 95% CI 61 more to 292 more) and probably improves quality of life (mean difference 0.04, 95% CI 0.00 to 0.08; PROMIS 29+2 Profile v2.1; range −0.022-1; higher scores indicate less impairment) versus providing one session of advice and support. Moderate certainty evidence also suggested that physical and mental health rehabilitation probably has little or no effect on physical and cognitive function. Moderate certainty evidence suggested that physical and mental health rehabilitation probably reduces symptoms of depression but may have little or no effect on symptoms of anxiety. No compelling evidence of benefit on fatigue, pain, or dyspnoea was found. We are very uncertain of the effects of the programme on serious adverse events.

## Discussion

Our systematic review and meta-analysis of 24 trials comprising 3695 patients with long covid identified moderate certainty evidence that an online CBT programme probably improves fatigue and concentration, and a programme of physical and mental health rehabilitation probably increases the proportion of patients who experience recovery or important improvements. We also found moderate certainty evidence suggesting that intermittent aerobic exercise probably improves physical function compared with continuous exercise. Effects of these interventions were modest, just reaching the MID for most outcomes.^110^

We did not find compelling evidence to support the effectiveness of other interventions, including, among others, vortioxetine, leronlimab, a synbiotic (SIM01), coenzyme Q10, amygdala and insula retraining, combined L-arginine and vitamin C, inspiratory muscle training, transcranial direct current stimulation, hyperbaric oxygen, and a mobile application providing education on long covid (telerehabilitation mobile app).

These findings, however, come with caveats. Long covid may be a heterogeneous condition, and it is unclear whether these interventions are broadly effective across all patients with long covid. For example, the evidence addressing physical and mental health rehabilitation came from patients who experienced severe acute covid-19 infection requiring hospital admission, and it is possible that effects may be different in patients with mild to moderate covid-19 infection.^110^

Promising interventions were investigated in single trials, and replication in other settings is required to inform generalisability. The success of interventions such as physical and mental health rehabilitation and CBT may depend on the fidelity with which they are replicated in future trials and settings, along with the experience of therapists. Notably, both the physical and mental health rehabilitation programme and CBT were delivered online, which can facilitate future widespread implementation.^100^ ^114^

Of most concern was our observation that one in four trials raised doubts about the integrity of the study results or execution. Such issues may not be immediately apparent to evidence users, potentially misleading patients and healthcare providers and adversely impacting care.

Our findings show that despite urgency and investments from research funding organisations, few randomised trials of interventions for long covid have been published. This might be due to most funds being allocated to observational research and mechanistic studies.^115^ This is an important finding, highlighting an opportunity for the health research community and funding organisations to re-evaluate their priorities.

### Relation to previous research

Both CBT and physical activity have long been shown to improve health and quality of life for people living with other chronic diseases.^116^ ^117^ ^118^ ^119^ Notably, both graduated physical activity and CBT have been found effective for myalgic encephalomyelitis (chronic fatigue syndrome or ME/CFS)—a condition with a striking resemblance to long covid that often emerges after viral infection.^120^ ^121^ ^122^

CBT and graduated physical activity are offered to patients with long covid and ME/CFS based on the observation that patients often reduce activity in response to their symptoms.^123^ Consequently, patients may become physically deconditioned, develop disrupted sleep-wake patterns, and hold unhelpful beliefs about fatigue.^124^ Interventions such as CBT and supervised physical activity which gradually reintroduce patients to activity may help with reconditioning, regularising patterns of activity, optimising rest and sleep, and addressing patients’ unhelpful beliefs about fatigue and activity. Despite supporting evidence, the role of exercise and CBT for long covid and other post-viral fatigue syndromes remains contentious, with some interpreting their success as evidence that the condition is “not real.”^45^ ^125^ ^126^ Our findings suggest it is reasonable to offer CBT and mental and physical rehabilitation to patients.

We emphasise that the effectiveness of CBT and physical rehabilitation for long covid neither indicates the condition is psychological nor negates a possible somatic cause. It is possible that CBT and physical rehabilitation only offer patients mechanisms to cope with symptoms from biological causes.

Preliminary evidence suggests that some patients with long covid may also present with alterations in gut flora (gut dysbiosis).^127^ ^128^ For example, investigators of the SIM01 trial have previously reported that several bacterial species, including *Bifidobacterium adolescentis* and *Bifidobacterium longum*, are all substantially lower in the gut of patients with covid-19 compared with healthy controls.^129^ ^130^ ^131^ The receptor for SARS-CoV-2, angiotensin converting enzyme 2, is widely expressed in the lining of the gut, and about 50% of patients with covid-19 present with vomiting, diarrhoea, and abdominal pain.^132^ Use of synbiotics to increase diversity of the gut microbiome could help to reduce some symptoms associated with long covid. The one single centre trial of synbiotics (SIM01) for long covid reported improvement in fatigue, concentration, and dyspnoea, but not quality of life.^103^ These findings, however, were supported by only low certainty evidence and require replication, ideally by non-conflicted investigators.

Finally, although previous research has addressed the long term consequences of other coronaviruses, including SARS-CoV-1 and MERS-CoV, such as damage to the respiratory system and cognitive sequelae, no research has addressed strategies for the management of these conditions.^133^

### Strengths and limitations of this review

Strengths of our systematic review include involvement of people with lived and living experience of long covid in the development of our protocol, a rigorous and comprehensive search for eligible trials, screening and extraction of data in duplicate, and a focus on patient important outcomes. We reported strengths and limitations of the evidence that may not be immediately apparent to evidence users, and used the GRADE approach to evaluate the certainty of evidence. We also reviewed trials for problems related to integrity that could call into question their conclusions.^63^

Our review also has limitations. Our methodological decisions were motivated by both rigor and feasibility. For example, we performed pragmatic searches of Google Scholar to identify reasonable ranges of MIDs to inform our judgements related to imprecision. Although systematic searches for MIDs may have offered a more comprehensive overview of MID estimates, performing these systematic searches for all measurement instruments would compromise our ability to perform timely updates of the review.

Despite our rigorous search of the literature, it is possible we missed eligible trials. We mitigated this issue by also reviewing the references of similar systematic reviews and soliciting experts to identify additional eligible trials that may not have come up in our search.

We assessed the certainty of evidence using the GRADE approach. Although this approach presents a comprehensive framework for systematically and transparently considering all factors that may bear on the certainty of evidence, its application is ultimately subjective, and others may come to different conclusions about the certainty of evidence.^134^

Our judgements about imprecision required knowledge of MIDs.^77^ For recovery, improvement, and serious adverse events, we established MIDs through discussion among the authorship group and patient partners. For continuous outcome measures, we sourced MIDs from the literature. It is possible for other investigators and patients to come to different conclusions about the certainty of evidence, depending on their threshold for what is considered a minimally important effect. Nonetheless, since we transparently reported all effect estimates and associated measures of precision, evidence users can make their own judgements considering alternative MIDs.

Likewise, although we attempted to identify untrustworthy trials due to data fabrication, falsification, or errors in conduct or analysis, it is possible that we may have missed some of these issues or misidentified trials. Nonetheless, methods to detect these problems in trials without individual participant data have poor sensitivity and specificity. It is possible that we missed some problematic trials or misclassified trustworthy trials as problematic.

We anticipated that the effects of interventions may vary according to diagnostic criteria for long covid, time since infection, number of infections, vaccination status, severity of acute covid-19, predominant long covid symptoms patients experienced, and SARS-CoV-2 variant, but encountered insufficient evidence in most circumstances to be able to investigate the influence of these factors on the effects of interventions.

Several trials recruited patients from social media groups, which may have included individuals without medically confirmed long covid.^102^ Nonetheless, established diagnostic criteria for long covid remain vague, and doctors are likely to encounter patients with self-diagnosed long covid.^23^

Our review relied on self-reported measures rather than observations by health professionals or biomarkers. This approach is justified since the symptoms of long covid, such as fatigue, are subjectively experienced, and no objective laboratory measures have been established to predict benefit in terms of how patients with long covid feel or function. Patient reported outcomes directly capture a patient’s own perceptions, experiences, and feelings, whereas laboratory or functional measures might not reflect the degree of impairment patients experience.^135^ ^136^ ^137^

### Implications

Our findings suggest that offering patients with long covid a programme of CBT or a programme of physical and mental health rehabilitation will probably improve symptoms. However, both CBT and physical and mental health rehabilitation require active patient engagement, which may be challenging owing to some patient groups expressing concerns about the safety and efficacy of these approaches and that the effectiveness of CBT and rehabilitation implies that long covid is not “real” but “psychological.”^43^ ^44^ ^45^

The evidence addressing CBT and physical and mental health rehabilitation was also at high risk of bias due to lack of blinding and imbalances in the degree of interactions between patients and healthcare providers between arms.^100^ ^110^ We suggest that future trials compare interventions with other active interventions, such as education or pacing programmes that include comparable interaction between patients and healthcare providers to reduce potential for expectancy effects.^138^

A trial of the synbiotic formulation SIM01 showed promising results,^103^ but independent investigators need to replicate these findings. Unlike CBT and physical and mental health rehabilitation, which multiple independent investigators have shown to be effective for similar conditions, this formulation of synbiotics, SIM01, has not been independently tested or shown to be effective for long covid or other conditions, except by its named innovators and patent holders.

Our findings have implications for the design of future studies on treatments for long covid in that only a single trial supported all interventions found to be effective.^139^ To maximise applicability, future trials should replicate these findings and include patients with a range of different phenotypes of long covid. Furthermore, only one of four drug interventions investigated in trials were FDA approved drugs, which is of concern because investigational drugs, even if found to be effective, will not be immediately available to patients. We also showed that, despite urgency, trial evidence testing interventions remains scarce. We call on the research community to identify efficiencies and prioritise randomised trials of promising interventions for long covid.

Currently, guidance on the optimal management of patients with long covid is limited. When guidance has been published, it is largely consensus based, does not base recommendations on rigorous systematic reviews, or provides limited advice on management.^140^ ^141^ ^142^ ^143^ For example, current guidance for the management of patients with long covid largely prioritises activity management (pacing) over physical activity owing to concerns about post-exertional malaise.^143^ ^144^ This symptom, frequently reported by patients with long covid and ME/CFS, involves worsening fatigue after physical or mental exertion.^43^ ^44^ ^45^ The trial we identified that investigated physical and mental health rehabilitation, however, did not report any instances of post-exertional malaise, despite closely monitoring patients for this symptom.^110^ Furthermore, a recent crossover trial found tailored exercise rehabilitation can be effective for long covid without escalation of symptoms.^145^ Together, these results suggest that interventions involving supervised, negotiated, and moderate physical activity can be safe for patients with long covid.

We trust that this systematic review will inform future guideline recommendations about the care of patients with long covid. We invite organisations responsible for the development of guidelines to join our committee of evidence users, who inform the type of data that we collect and our methodological approaches to ensure that our products align with their needs.

### Conclusion

Moderate certainty evidence suggests that a programme of CBT probably reduces fatigue and improves cognitive function in patients with long covid, and a programme of physical and mental health rehabilitation probably increases the proportion of patients who experience recovery or important improvements.

**What is already known on this topic**

Although most patients recover from covid-19, up to 15% might experience long term health effects, including fatigue, myalgia, and impaired cognitive function
Healthcare providers increasingly encounter patients with long covid, and, in the absence of trustworthy and up-to-date summaries of the evidence, patients may receive unproven, costly, and ineffective or harmful treatments

**What this study adds**

Moderate certainty evidence suggests that cognitive behavioural therapy and physical and mental health rehabilitation are probably effective for the treatment of long covid
Moderate certainty evidence suggests that intermittent aerobic exercise probably improves physical function compared with continuous aerobic exercise
No compelling evidence supported the effectiveness of other interventions, including, among others, vortioxetine, leronlimab, a synbiotic (SIM01), coenzyme Q10, amygdala and insula retraining, combined L-arginine and vitamin C, inspiratory muscle training, transcranial direct current stimulation, hyperbaric oxygen, and a mobile application providing education on long covid (telerehabilitation mobile app)

## Data Availability

Additional data are available at https://osf.io/9h7zm/.

## Web extra

Extra material supplied by authors

## Notes

Contributors: DZ and JWB conceived the study. RJC performed the literature search. ML, SK, TJ, MS, HM, JRK, and AW collected data. DZ and ML performed data analysis and synthesis. DZ and ML wrote the first draft of the manuscript. All authors reviewed the manuscript and provided critical feedback. DZ and JWB are the guarantors. The corresponding author attests that all listed authors meet authorship criteria and that no others meeting the criteria have been omitted.

Transparency: The lead author and the last author (the manuscript’s guarantors) affirm that the manuscript is an honest, accurate, and transparent account of the study being reported; that no important aspects of the study have been omitted; and that any discrepancies from the study as planned (and, if relevant, registered) have been explained.

Dissemination to participants and related patient and public communities: We intend to disseminate our results via our connections to long covid organisations and advocacy groups, such as Long Covid Web.

Provenance and peer review: Not commissioned; externally peer reviewed.
