# Supplementary material for: Interventions for the management of long covid (post-covid condition): living systematic review
Source: BMJ. 2024 Nov 27;387:e081318. doi: 10.1136/bmj-2024-081318 (PMC11600537; doi:10.1136/bmj-2024-081318)
Supplement: Supplementary file 1 — Supplementary information: Supplements 1-30 [file zerd081318.ww.pdf]

Interventions for the management of long COVID (post COVID) condition: a living systematic review

Contents

Supplement 1. Summary of search strategy 4
Supplement 2. Minimal important differences of measurement tools 14
Supplement 3. Trial characteristics 51
Supplement 4. Risk of bias of trials reporting on pharmacologic interventions 77
Supplement 5. Risk of bias of trials reporting on physical activity and rehabilitative interventions 78
Supplement 6. Risk of bias of trials reporting on behavioral interventions 79
Supplement 7. Risk of bias of trials reporting on dietary supplements and other dietary interventions 80
Supplement 8. Risk of bias of trials reporting on medical devices and technologies 81
Supplement 9: Risk of bias of trials reporting on combination therapies 82
Supplement 10: Summary of findings table comparing Vortioxetine and Placebo 83
Supplement 11: Summary of findings table comparing Leronlimab and Placebo 84
Supplement 12: Summary of findings table comparing Glucosaminyl muramyl dipeptide ('Licopid') and Usual care 85
Supplement 13: Summary of findings table comparing Actovegin and Usual care 88
Supplement 14: Summary of findings table comparing Physiotherapy, Multicomponent exercise of progressively increasing intensity and Physiotherapy 89
Supplement 15: Summary of findings table comparing Intermittent aerobic exercise and Continuous aerobic exercise 91
Supplement 16: Summary of findings table comparing Low-intensity aerobic exercise, Strength training and High-intensity aerobic exercise, Strength training 92
Supplement 17: Summary of findings table comparing Inspiratory muscle training and Usual care 93
Supplement 18: Summary of findings table comparing Active cycle of breathing technique, Physiotherapy and Physiotherapy 94
Supplement 19: Summary of findings table comparing In-patient rehabilitation, Physiotherapy, Acupuncture and In-patient rehabilitation, Physiotherapy 95
Supplement 20: Summary of findings table comparing an Online cognitive behavioral therapy (CBT) program called 'Fit after Covid' and Usual care 96
Supplement 21: Summary of findings table comparing a Mobile application providing education on long COVID ('telerehabilitation mobile app') and Usual care 98
Supplement 22: Summary of findings table comparing Amygdala and insula retraining and Education related to self-management 99
Supplement 23: Summary of findings table comparing a formulation of Probiotics and prebiotics ('Synbiotics') called SIM01 and Placebo 100

Supplement 24: Summary of findings table comparing Coenzyme Q10 and Placebo 102

Supplement 25: Summary of findings table comparing a combination of L-arginine, vitamin C and Placebo 103

Supplement 26: Summary of findings table comparing Brainmax and Placebo 104

Supplement 27: Summary of findings table comparing Hyperbaric oxygen therapy and Placebo 106

Supplement 28: Summary of findings table comparing Transcranial direct current stimulation, Physiotherapy, Education related to activities of daily living and Physiotherapy, Education related to self-management 108

Supplement 29: Summary of findings table comparing Photobiomodulation and Placebo 110

Supplement 30: Summary of findings table comparing Physical and mental health rehabilitation and Usual care 111

Supplement 1. Summary of search strategy

Summary of search and strategies long COVID RCTs

MEDLINE	3145
Embase	3855
PsycInfo	96
AMED	14
CINAHL	733
Central	6088
Subtotal	13931
-dupes	-2257
Total	11674

Search strategy adapted from Campbell SM. Filter to Retrieve Studies Related to Long COVID in the OVID Medline Database. John W. Scott Health Sciences Library, University of Alberta, Rev. Jan 10, 2023.. https://docs.google.com/document/d/1iEN3WvRGAtj_NF60LUgElBA4FEcCdOgW1gcadK45ltE/edit#

Nov 30, 2023

MEDLINE (OVID)

Database: OVID Medline Epub Ahead of Print, In-Process & Other Non-Indexed Citations, Ovid MEDLINE(R) Daily and Ovid MEDLINE(R) 1946 to Present

Search Strategy:

----

1 Post-Acute COVID-19 Syndrome/ (2693)

2 (long* adj3 (covid or covid-19 or covid19 or sars cov 2)).mp. [mp=title, book title, abstract, original title, name of substance word, subject heading word, floating sub-heading word, keyword heading word, organism supplementary concept word, protocol supplementary concept word, rare disease supplementary concept word, unique identifier, synonyms, population supplementary concept word, anatomy supplementary concept word] (6413)

3 ((covid* or "corona virus 2019" or "coronavirus2019" or SARS-CoV-2 or sars cov 2) adj3 (syndrome or persist* or lingering or chronic or ongoing or long-term or "long term" or long-haul or "long haul" or convalescen* or rehabilitat*)).ti. (5492)

4 ((exp SARS-CoV-2/ or exp COVID-19/) and sequela*.ti,ab.) or ("long Covid" or ((Covid or Covid19 or "corona virus 2019" or "coronavirus 2019" or SARS-CoV-2 or "B.1.1.7" or "B.1.351" or "B.1.1.28" or "B.1.617" or "BA.1" or "BA.2" or "BA.3" or "BA.4" or "BA.5" or omicron or deltacron or "delta variant" or "delta subvariant" or "XBB.1.3") adj3 (PASC or sequela* or "post acute" or postacute or prolonged or "long haul*" or chronic or lingering or ongoing or persistent or "long term" or "more than 12 weeks" or "more than 24 weeks"))).mp. (14366)

Annotation: Sandra Campbell filter

5 1 or 2 or 3 or 4 (18940)

6 randomized controlled trial.pt. (604012)

7 controlled clinical trial.pt. (95469)

8 randomi?ed.ab. (747389)

9 placebo.ab. (243520)

10 drug therapy.fs. (2643985)

11 randomly.ab. (421817)

12 trial.ab. (674743)

13 groups.ab. (2602907)

14 or/6-13 (5837395)

15 exp animals/ not humans.sh. (5174725)

16 14 not 15 (5102329)

17 5 and 16 (3145)

Embase (OVID)

Database: Embase <1974 to 2023 November 29>

Search Strategy:

----

1 long COVID/ (6134)

2 (long* adj3 (covid or covid-19 or covid19 or sars cov 2)).mp. [mp=title, abstract, heading word, drug trade name, original title, device manufacturer, drug manufacturer, device trade name, keyword heading word, floating subheading word, candidate term word] (9976)

3 ((covid* or "corona virus 2019" or "coronavirus2019" or SARS-CoV-2 or sars cov 2) adj3 (syndrome or persist* or lingering or chronic or ongoing or long-term or "long term" or long-haul or "long haul" or convalescen* or rehabilitat*)).ti. (6624)

4 exp coronavirus disease 2019/ and sequela*.ti,ab. (4716)

5 ((Covid or Covid19 or "corona virus 2019" or "coronavirus 2019" or SARS-CoV-2 or "B.1.1.7" or "B.1.351" or "B.1.1.28" or "B.1.617" or "BA.1" or "BA.2" or "BA.3" or "BA.4" or "BA.5" or omicron or deltacron or "delta variant" or "delta subvariant" or "XBB.1.3") adj3 (PASC or sequela* or "post acute" or postacute or prolonged or "long haul*" or chronic or lingering or ongoing or persistent or "long term" or "more than 12 weeks" or "more than 24 weeks")).mp. [mp=title, abstract, heading word, drug trade name, original title, device manufacturer, drug manufacturer, device trade name, keyword heading word, floating subheading word, candidate term word] (12484)

6 or/1-5 (24627)

7 randomized controlled trial/ (795458)

8 Controlled clinical study/ (471569)

9 random$.ti,ab. (2003505)

10 randomization/ (98950)

11 intermethod comparison/ (302807)

12 placebo.ti,ab. (369125)

13 (compare or compared or comparison).ti. (611285)

14 ((evaluated or evaluate or evaluating or assessed or assess) and (compare or compared or comparing or comparison)).ab. (2822956)

15 (open adj label).ti,ab. (111156)

16 ((double or single or doubly or singly) adj (blind or blinded or blindly)).ti,ab. (276603)

17 double blind procedure/ (213168)

18 parallel group$1.ti,ab. (32580)

19 (crossover or cross over).ti,ab. (125730)

20 ((assign$ or match or matched or allocation) adj5 (alternate or group$1 or intervention$1 or patient$1 or subject$1 or participant$1)).ti,ab. (420795)

21 (assigned or allocated).ti,ab. (497209)

22 (controlled adj7 (study or design or trial)).ti,ab. (456970)

23 (volunteer or volunteers).ti,ab. (284653)

24 human experiment/ (651172)

25 trial.ti. (408345)

26 or/7-25 (6411600)

27 (random$ adj sampl$ adj7 ("cross section$" or questionnaire$1 or survey$ or database$1)).ti,ab. not (comparative study/ or controlled study/ or randomi?ed controlled.ti,ab. or randomly assigned.ti,ab.) (9678)

28 Cross-sectional study/ not (randomized controlled trial/ or controlled clinical study/ or controlled study/ or randomi?ed controlled.ti,ab. or control group$1.ti,ab.) (368853)

29 (((case adj control$) and random$) not randomi?ed controlled).ti,ab. (21826)

30 (Systematic review not (trial or study)).ti. (267248)

31 (nonrandom$ not random$).ti,ab. (19102)

32 "Random field$".ti,ab. (2993)

33 (random cluster adj3 sampl$).ti,ab. (1604)

34 (review.ab. and review.pt.) not trial.ti. (1150148)

35 "we searched".ab. and (review.ti. or review.pt.) (50455)

36 "update review".ab. (137)

37 (databases adj4 searched).ab. (64166)

38 (rat or rats or mouse or mice or swine or porcine or murine or sheep or lambs or pigs or piglets or rabbit or rabbits or cat or cats or dog or dogs or cattle or bovine or monkey or monkeys or trout or marmoset$1).ti. and animal experiment/ (1232332)

39 Animal experiment/ not (human experiment/ or human/) (2587994)

40 or/27-39 (4401726)

41 26 not 40 (5654324)

42 6 and 41 (3855)

PsycInfo OVID

Database: APA PsycInfo <1806 to November Week 3 2023>

Search Strategy:

----

1 post-covid-19 conditions/ (140)

2 (long* adj3 (covid or covid-19 or covid19 or sars cov 2)).mp. (476)

3 ((covid* or "corona virus 2019" or "coronavirus2019" or SARS-CoV-2 or sars cov 2) adj3 (syndrome or persist* or lingering or chronic or ongoing or long-term or "long term" or long-haul or "long haul" or convalescen* or rehabilitat*)).ti. (190)

4 exp covid-19/ and sequela*.ti,ab. (276)

5 ((Covid or Covid19 or "corona virus 2019" or "coronavirus 2019" or SARS-CoV-2 or "B.1.1.7" or "B.1.351" or "B.1.1.28" or "B.1.617" or "BA.1" or "BA.2" or "BA.3" or "BA.4" or "BA.5" or omicron or deltacron or "delta variant" or "delta subvariant" or "XBB.1.3") adj3 (PASC or sequela* or "post acute" or postacute or prolonged or "long haul*" or chronic or lingering or ongoing or persistent or "long term" or "more than 12 weeks" or "more than 24 weeks")).mp. [mp=title, abstract, heading word, table of contents, key concepts, original title, tests & measures, mesh word] (877)

6 or/1-5 (1399)

7 clinical trials/ (12263)

8 random:.tw. or placebo:.mp. or double-blind:.tw. (273444)

9 ((treatment or control) adj3 group*).ab. (125944)

10 (allocat* adj5 group*).ab. (3242)

11 ((clinical or control*) adj3 trial).ti,ab. (55610)

12 or/7-11 (371422)

13 6 and 12 (96)

AMED (OVID)

Database: AMED (Allied and Complementary Medicine) <1985 to October 2023>

Search Strategy:

----

1 (long* adj3 (covid or covid-19 or covid19 or sars cov 2)).mp. (40)

2 ((covid* or "corona virus 2019" or "coronavirus2019" or SARS-CoV-2 or sars cov 2) adj3 (syndrome or persist* or lingering or chronic or ongoing or long-term or "long term" or long-haul or "long haul" or convalescen* or rehabilitat*)).ti. (100)

3 ((Covid or Covid19 or "corona virus 2019" or "coronavirus 2019" or SARS-CoV-2 or "B.1.1.7" or "B.1.351" or "B.1.1.28" or "B.1.617" or "BA.1" or "BA.2" or "BA.3" or "BA.4" or "BA.5" or omicron or deltacron or "delta variant" or "delta subvariant" or "XBB.1.3") adj3 (PASC or sequela* or "post acute" or postacute or prolonged or "long haul*" or chronic or lingering or ongoing or persistent or "long term" or "more than 12 weeks" or "more than 24 weeks")).mp. (80)

4 or/1-3 (159)

5 exp clinical trials/ (5249)

6 random:.tw. or placebo:.mp. or double-blind:.tw. (27785)

7 ((treatment or control) adj3 group*).ab. (15197)

8 (allocat* adj5 group*).ab. (1155)

9 ((clinical or control*) adj3 trial).ti,ab. (10341)

10 or/5-9 (37012)

11 4 and 10 (14)

CINAHL (EBSCO)

Thursday, November 30, 2023 8:45:56 PM				
**#**	**Query**	**Limiters/Expanders**	**Last Run Via**	**Results**
S32	S23 AND S31	Search modes - Boolean/Phrase	Interface - EBSCOhost Research Databases	733

			Search Screen - Advanced Search<br>Database - CINAHL	
S31	S24 OR S25 OR S26 OR S27 OR S30	Search modes - Boolean/Phrase	Interface - EBSCOhost Research Databases<br>Search Screen - Advanced Search<br>Database - CINAHL	7,822
S30	S28 AND S29	Search modes - Boolean/Phrase	Interface - EBSCOhost Research Databases<br>Search Screen - Advanced Search<br>Database - CINAHL	398
S29	TX sequela*	Search modes - Boolean/Phrase	Interface - EBSCOhost Research Databases<br>Search Screen - Advanced Search<br>Database - CINAHL	17,889
S28	(MH "COVID-19+")	Search modes - Boolean/Phrase	Interface - EBSCOhost Research Databases<br>Search Screen - Advanced Search<br>Database - CINAHL	45,583
S27	TX ((Covid or Covid19 or "corona virus 2019" or "coronavirus 2019" or SARS-CoV-2 or "B.1.1.7" or "B.1.351" or "B.1.1.28" or "B.1.617" or "BA.1" or "BA.2" or "BA.3" or "BA.4" or "BA.5" or omicron or deltacron or "delta variant" or "delta subvariant" or "XBB.1.3") N3 (PASC or sequela* or "post acute" or postacute or prolonged or "long haul*" or chronic or lingering or ongoing or persistent or "long term" or "more than 12	Search modes - Boolean/Phrase	Interface - EBSCOhost Research Databases<br>Search Screen - Advanced Search<br>Database - CINAHL	4,549

	weeks" or "more than 24 weeks"))			
S26	TI ((covid* or "corona virus 2019" or "coronavirus2019" or SARS-CoV-2 or sars cov 2) N3 (syndrome or persist* or lingering or chronic or ongoing or long-term or "long term" or long-haul or "long haul" or convalescen* or rehabilitat*))	Search modes - Boolean/Phrase	Interface - EBSCOhost Research Databases<br>Search Screen - Advanced Search<br>Database - CINAHL	2,806
S25	TX (long* N3 (covid or covid-19 or covid19 or sars cov 2))	Search modes - Boolean/Phrase	Interface - EBSCOhost Research Databases<br>Search Screen - Advanced Search<br>Database - CINAHL	2,826
S24	(MH "Post-Acute COVID-19 Syndrome")	Search modes - Boolean/Phrase	Interface - EBSCOhost Research Databases<br>Search Screen - Advanced Search<br>Database - CINAHL	1,047
S23	S22 NOT S21	Expanders - Apply equivalent subjects<br>Search modes - Boolean/Phrase	Interface - EBSCOhost Research Databases<br>Search Screen - Advanced Search<br>Database - CINAHL	981,337
S22	S1 OR S2 OR S3 OR S4 OR S5 OR S6 OR S7 OR S8 OR S9 OR S10 OR S11 OR S12 OR S13 OR S14 OR S15	Expanders - Apply equivalent subjects<br>Search modes - Boolean/Phrase	Interface - EBSCOhost Research Databases<br>Search Screen - Advanced Search<br>Database - CINAHL	1,029,252
S21	S19 NOT S20	Expanders - Apply equivalent subjects<br>Search modes - Boolean/Phrase	Interface - EBSCOhost Research Databases<br>Search Screen - Advanced Search<br>Database - CINAHL	214,511
S20	MH (human)	Expanders - Apply equivalent subjects<br>Search modes - Boolean/Phrase	Interface - EBSCOhost Research Databases<br>Search Screen - Advanced Search<br>Database - CINAHL	2,730,925

S19	S16 OR S17 OR S18	Expanders - Apply equivalent subjects<br>Search modes - Boolean/Phrase	Interface - EBSCOhost Research Databases<br>Search Screen - Advanced Search<br>Database - CINAHL	248,624
S18	TI (animal model*)	Expanders - Apply equivalent subjects<br>Search modes - Boolean/Phrase	Interface - EBSCOhost Research Databases<br>Search Screen - Advanced Search<br>Database - CINAHL	3,599
S17	MH (animal studies)	Expanders - Apply equivalent subjects<br>Search modes - Boolean/Phrase	Interface - EBSCOhost Research Databases<br>Search Screen - Advanced Search<br>Database - CINAHL	154,533
S16	MH animals+	Expanders - Apply equivalent subjects<br>Search modes - Boolean/Phrase	Interface - EBSCOhost Research Databases<br>Search Screen - Advanced Search<br>Database - CINAHL	103,115
S15	AB (cluster W3 RCT)	Expanders - Apply equivalent subjects<br>Search modes - Boolean/Phrase	Interface - EBSCOhost Research Databases<br>Search Screen - Advanced Search<br>Database - CINAHL	503
S14	MH (crossover design) OR MH (comparative studies)	Expanders - Apply equivalent subjects<br>Search modes - Boolean/Phrase	Interface - EBSCOhost Research Databases<br>Search Screen - Advanced Search<br>Database - CINAHL	482,768
S13	AB (control W5 group)	Expanders - Apply equivalent subjects<br>Search modes - Boolean/Phrase	Interface - EBSCOhost Research Databases<br>Search Screen - Advanced Search<br>Database - CINAHL	146,556
S12	PT (randomized controlled trial)	Expanders - Apply equivalent subjects<br>Search modes - Boolean/Phrase	Interface - EBSCOhost Research Databases<br>Search Screen - Advanced Search<br>Database - CINAHL	153,885
S11	MH (placebos)	Expanders - Apply equivalent subjects<br>Search modes - Boolean/Phrase	Interface - EBSCOhost Research Databases<br>Search Screen - Advanced Search<br>Database - CINAHL	13,875
S10	MH (sample size) AND AB (assigned OR allocated OR control)	Expanders - Apply equivalent subjects<br>Search modes - Boolean/Phrase	Interface - EBSCOhost Research Databases<br>Search Screen - Advanced Search<br>Database - CINAHL	4,452
S9	TI (trial)	Expanders - Apply equivalent subjects<br>Search modes - Boolean/Phrase	Interface - EBSCOhost Research Databases<br>Search Screen - Advanced Search<br>Database - CINAHL	186,494

S8	AB (random*)	Expanders - Apply equivalent subjects<br>Search modes - Boolean/Phrase	Interface - EBSCOhost Research Databases<br>Search Screen - Advanced Search<br>Database - CINAHL	403,154
S7	TI (randomised OR randomized)	Expanders - Apply equivalent subjects<br>Search modes - Boolean/Phrase	Interface - EBSCOhost Research Databases<br>Search Screen - Advanced Search<br>Database - CINAHL	145,326
S6	MH cluster sample	Expanders - Apply equivalent subjects<br>Search modes - Boolean/Phrase	Interface - EBSCOhost Research Databases<br>Search Screen - Advanced Search<br>Database - CINAHL	5,356
S5	MH pretest-posttest design	Expanders - Apply equivalent subjects<br>Search modes - Boolean/Phrase	Interface - EBSCOhost Research Databases<br>Search Screen - Advanced Search<br>Database - CINAHL	54,200
S4	MH random assignment	Expanders - Apply equivalent subjects<br>Search modes - Boolean/Phrase	Interface - EBSCOhost Research Databases<br>Search Screen - Advanced Search<br>Database - CINAHL	82,143
S3	MH single-blind studies	Expanders - Apply equivalent subjects<br>Search modes - Boolean/Phrase	Interface - EBSCOhost Research Databases<br>Search Screen - Advanced Search<br>Database - CINAHL	16,069
S2	MH double-blind studies	Expanders - Apply equivalent subjects<br>Search modes - Boolean/Phrase	Interface - EBSCOhost Research Databases<br>Search Screen - Advanced Search<br>Database - CINAHL	54,318
S1	MH randomized controlled trials	Expanders - Apply equivalent subjects<br>Search modes - Boolean/Phrase	Interface - EBSCOhost Research Databases<br>Search Screen - Advanced Search<br>Database - CINAHL	140,118

Cochrane Library (Wiley)

Search Name: 2023-11-30 Long Covid revised

Date Run: 30/11/2023 22:23:02

Comment:

ID Search Hits

#1 MeSH descriptor: [Post-Acute COVID-19 Syndrome] explode all trees 78

#2 (((covid* or "corona virus 2019" or "coronavirus2019" or SARS-CoV-2 or sars cov 2) NEAR/3 (syndrome or persist* or lingering or chronic or ongoing or long-term or "long term" or long-haul or "long haul" or convalescen* or rehabilitat*))):ti (Word variations have been searched) 1294

#3 ((Covid or Covid19 or "corona virus 2019" or "coronavirus 2019" or SARS-CoV-2 or "B.1.1.7" or "B.1.351" or "B.1.1.28" or "B.1.617" or "BA.1" or "BA.2" or "BA.3" or "BA.4" or "BA.5" or omicron or deltacron or "delta variant" or "delta subvariant" or "XBB.1.3") NEAR/3 (PASC or sequela* or "post acute" or postacute or prolonged or "long haul" or chronic or lingering or ongoing or persistent or "long term" or "more than 12 weeks" or "more than 24 weeks")) 608

#4 MeSH descriptor: [COVID-19] explode all trees 4984

#5 MeSH descriptor: [SARS-CoV-2] explode all trees 2457

#6 #4 or #5 5198

#7 sequela* 5641

#8 #6 and #7 94

#9 long covid 1900

#10 long NEAR/3 (covid or covid19 or covid 19 or sars cov 2) 3808

#11 (long-haul or long-term) NEAR/3 (covid or covid19 or covid 19 or sars cov 2) 2171

#12 #1 or #2 or #3 or #8 or #9 or #10 or #11 in Trials 6088

Supplement 2. Minimal important differences of measurement tools

Measure	MID	Lowest	Highest	Higher scores mean	Study	Population	Number of participants	Method for estimation of MID	Anchor
Brief Pain Inventory (BPI) pain severity subscale	2.2	0	10	More impairment	Mease PJ, Spaeth M, Clauw DJ, Arnold LM, Bradley LA, Russell IJ, et al. Estimation of minimum clinically important difference for pain in fibromyalgia. Arthritis Care & Research. 2011;63(6):821-6.	Patients with fibromyalgia randomized to duloxetine or placebo	489	Average change approach	Patient's Global Impressions of Improvement scale (PGI-I) (clinically stable: 4 or minimal clinically relevant improvement: 2)
Brief Pain Inventory (BPI) pain severity subscale	1.15*	0	10	More impairment	Zilberman-Itskovich S, Catalogna M, Sasson E, Elman-Shina K, Hadanny A, Lang E, et al. Hyperbaric oxygen therapy improves neurocognitive functions and symptoms of post-COVID condition: randomized controlled trial. Sci Rep. 2022;12(1):11252.				
Brief Symptom Inventory-18 (BSI-18)	6.2*	0	72	More impairment	Zilberman-Itskovich S, Catalogna M, Sasson E, Elman-				

					Shina K, Hadanny A, Lang E, et al. Hyperbaric oxygen therapy improves neurocognitive functions and symptoms of post-COVID condition: randomized controlled trial. Sci Rep. 2022;12(1):11252.
Brief Symptom Inventory-18 (BSI-18) anxiety subscale	2.65*	0	24	More impairment	Zilberman-Itskovich S, Catalogna M, Sasson E, Elman-Shina K, Hadanny A, Lang E, et al. Hyperbaric oxygen therapy improves neurocognitive functions and symptoms of post-COVID condition: randomized controlled trial. Sci Rep. 2022;12(1):11252.
Brief Symptom Inventory-18 (BSI-18) depression subscale	3.05*	0	24	More impairment	Zilberman-Itskovich S, Catalogna M, Sasson E, Elman-Shina K, Hadanny A, Lang E, et al. Hyperbaric oxygen therapy improves neurocognitive functions and

					symptoms of post-COVID condition: randomized controlled trial. Sci Rep. 2022;12(1):11252.
Brief Symptom Inventory-18 (BSI-18) somatization subscale	3*	0	24	More impairment	Zilberman-Itskovich S, Catalogna M, Sasson E, Elman-Shina K, Hadanny A, Lang E, et al. Hyperbaric oxygen therapy improves neurocognitive functions and symptoms of post-COVID condition: randomized controlled trial. Sci Rep. 2022;12(1):11252.
Checklist Individual Strength (CIS) concentration problems subscale	3.4*	5	35	More impairment	Kuut TA, Muller F, Csorba I, Braamse A, Aldenkamp A, Appelman B, et al. Efficacy of Cognitive-Behavioral Therapy Targeting Severe Fatigue Following Coronavirus Disease 2019: Results of a Randomized Controlled Trial.

					Clin Infect Dis. 2023;77(5):687-95.				
Checklist Individual Strength (CIS) fatigue subscale	9.3	8	56	More impairment	Rebelo P, Oliveira A, Andrade L, Valente C, Marques A. Minimal Clinically Important Differences for Patient-Reported Outcome Measures of Fatigue in Patients With COPD Following Pulmonary Rehabilitation. Chest. 2020;158(2):550-61.	Patients with COPD following pulmonary rehabilitation	53	Arithmetic weighted mean, resulting from the combination of anchor-based (weight, two-thirds): average change approach and distribution-based (weight, one-third) methods: 0.5xSD, SE of measurement, 1.96xSE of measurement, and minimal detectable change	Acute exacerbation of COPD (AECOPD)
Checklist Individual Strength (CIS) fatigue subscale	3*	8	56	More impairment	Kuut TA, Muller F, Csorba I, Braamse A, Aldenkamp A, Appelman B, et al. Efficacy of Cognitive-Behavioral Therapy Targeting Severe Fatigue Following Coronavirus Disease 2019: Results of a Randomized				

					Controlled Trial. Clin Infect Dis. 2023;77(5):687-95.				
Checklist Individual Strength (CIS) fatigue subscale	9.6	8	56	More impairment	Rebelo P, Oliveira A, Andrade L, Valente C, Marques A. Minimal Clinically Important Differences for Patient-Reported Outcome Measures of Fatigue in Patients With COPD Following Pulmonary Rehabilitation. Chest. 2020;158(2):550-61.	Patients with COPD following pulmonary rehabilitation	53	Average change approach	Acute exacerbation of COPD (AECOPD)
Digital Symbol Substitution Test	5	0	Infinity	Less impairment	Jehu DA, Davis JC, Madden K, Parmar N, Liu-Ambrose T. Minimal Clinically Important Difference of Executive Function Performance in Older Adults Who Fall: A Secondary Analysis of a Randomized Controlled Trial. Gerontology. 2021;68(7):771-9.	Patients with falls randomized to Otago Exercise Program (OEP) or usual care	179	Average change approach	Montreal Cognitive Assessment (MoCA) (≥3 or ≤ −3 points)

Digital Symbol Substitution Test	2.5	0	Infinity	Less impairment	Jehu DA, Davis JC, Madden K, Parmar N, Liu-Ambrose T. Minimal Clinically Important Difference of Executive Function Performance in Older Adults Who Fall: A Secondary Analysis of a Randomized Controlled Trial. Gerontology. 2021;68(7):771-9.	Patients with falls randomized to Otago Exercise Program (OEP) or usual care	114	ROC curve	Montreal Cognitive Assessment (MoCA) (≥3 or ≤ −3 points)
EQ-5D health index	0.03 to 0.3	0	100	Less impairment	Coretti S, Ruggeri M, McNamee P. The minimum clinically important difference for EQ-5D index: a critical review. Expert Review of Pharmacoeconomics & Outcomes Research. 2014;14(2):221-33.	Patients with low back pain and undergoing lumbar disc herniation surgery	NA	Systematic review of published studies	NA
EQ-5D health index	0.03	0	100	Less impairment	Soer R, Reneman MF, Speijer BLGN, Coppes MH, Vroomen PCAJ. Clinimetric properties of the EuroQol-5D in patients with chronic low back	Patients with low back pain	151	ROC curve	Roland Morris Disability Questionnaire, Numeric rating scale, Pain Disability Index

					pain. The Spine Journal. 2012;12(11):1035-9.				
EQ-5D health index	0.05	0	100	Less impairment	Le QA, Doctor JN, Zoellner LA, Feeny NC. Minimal clinically important differences for the EQ-5D and QWB-SA in Post-traumatic Stress Disorder (PTSD): results from a Doubly Randomized Preference Trial (DRPT). Health and Quality of Life Outcomes. 2013;11(1):59.	Patients with PTSD randomized to cognitive behavioral therapy or sertraline	155	Regression method	Clinical Global Impression-Severity (CGI-S)
EQ-5D health index	0.08	0	100	Less impairment	Le QA, Doctor JN, Zoellner LA, Feeny NC. Minimal clinically important differences for the EQ-5D and QWB-SA in Post-traumatic Stress Disorder (PTSD): results from a Doubly Randomized Preference Trial (DRPT). Health and Quality of Life Outcomes. 2013;11(1):59.	Patients with PTSD randomized to cognitive behavioral therapy or sertraline	155	Regression method	Clinical Global Impression-Improvement (CGI-I) (clinically meaningful improvement: ≤3

Fatigue Assessment Scale-10 (FAS-10)	3.5	10	50	More impairment	de Kleijn WPE, De Vries J, Wijnen PAHM, Drent M. Minimal (clinically) important differences for the Fatigue Assessment Scale in sarcoidosis. Respiratory Medicine. 2011;105(9):1388-95.	Patients with sarcoidosis	321	ROC curve	World Health Organization Quality of Life BREF (WHOQOL-BREF) (improved: ≥1.63 or worsened: ≤-1.63)
Fatigue Severity Scale (FSS)	0.5 to 1.2	1	7	More impairment	Rooney S, McFadyen DA, Wood DL, Moffat DF, Paul PL. Minimally important difference of the fatigue severity scale and modified fatigue impact scale in people with multiple sclerosis. Multiple Sclerosis and Related Disorders. 2019;35:158-63.	Patients with multiple sclerosis	365	Regression method	3 items EQ-5D, 23 items Multiple Sclerosis Impact Scale-29 (MSIS-29) (worsened or improved)
Fatigue Severity Scale (FSS)	20.2	9	63	More impairment	Pouchot J, Kherani RB, Brant R, Lacaille D, Lehman AJ, Ensworth S, et al. Determination of the minimal clinically important difference for	Patients with rheumatoid arthritis	61	Regression method	Patient global impression of fatigue compared to peers

					seven fatigue measures in rheumatoid arthritis. Journal of Clinical Epidemiology. 2008;61(7):705-13.				
Fatigue Severity Scale (FSS)	0.6	9	63	More impairment	Ewan CG, Jacques P, Rollin B, Raheem BK, Aviña-Zubieta JA, Diane L, et al. Minimal clinically important difference for 7 measures of fatigue in patients with systemic lupus erythematosus. The Journal of Rheumatology. 2008;35(4):635.	Patients with systemic lupus erythematosus (SLE)	80	Regression method	Global assessment of fatigue using an 11-point numerical rating scale (NRS) (no fatigue at all: 0 or fatigue as bad as it could be: 10)
Fatigue Severity Scale (FSS)	0.5 to 1.2	9	63	More impairment	Nordin Å, Taft C, Lundgren-Nilsson Å, Dencker A. Minimal important differences for fatigue patient reported outcome measures—a systematic review. BMC Medical Research Methodology. 2016;16(1):62.	Patients with systemic lupus erythematosus (SLE), rheumatoid arthritis (RA), and multiple sclerosis (MS)	NA	Systematic review of published studies	NA

Hamilton Anxiety Rating Scale (HAM-A)	4	0	56	More impairment	Fan J-q, Lu W-j, Tan W-q, Liu X, Wang Y-t, Wang N-b, Zhuang L-x. Effectiveness of Acupuncture for Anxiety Among Patients With Parkinson Disease: A Randomized Clinical Trial. JAMA Network Open. 2022;5(9):e2232133-e.	Patients with Parkinson's disease and undergoing acupuncture	64	Effective standard method (Score difference of patients who differ by at least one grade in the anchor options before and after the intervention calculated. If the difference obeys the normal distribution, the mean value of the difference is taken as the MCID. If the difference follows a skew distribution, the median is MCID.)	Unified Parkinson’s Disease Rating Scale (cutoff: 1)
Hamilton Anxiety Rating Scale (HAM-A)	3*	0	56	More impairment	Santana K, Franca E, Sato J, Silva A, Queiroz M, de Farias J, et al. Non-invasive brain stimulation for fatigue in post-acute sequelae of SARS-CoV-2 (PASC).				

					Brain Stimul. 2023;16(1):100-7.				
Hospital Anxiety and Depression Scale (HADS)	1.5	0	21	More impairment	Puhan MA, Frey M, Büchi S, Schünemann HJ. The minimal important difference of the hospital anxiety and depression scale in patients with chronic obstructive pulmonary disease. Health Qual Life Outcomes. 2008;6:46.	Patients with chronic obstructive pulmonary disease	88	Regression method	Chronic Respiratory Questionnaire (CRQ) (cutoff: 0.5), Feeling Thermometer (cutoff: 8 points)
Hospital Anxiety and Depression Scale (HADS) anxiety subscale	1.5	0	21	More impairment	Lemay KR, Tulloch HE, Pipe AL, Reed JL. Establishing the Minimal Clinically Important Difference for the Hospital Anxiety and Depression Scale in Patients With Cardiovascular Disease. Journal of Cardiopulmonary Rehabilitation and Prevention. 2019;39(6).	Patients with cardiovascular disease undergoing a cardiac rehabilitation program	591	ROC curve	Item 2 of the SF-36 (ie, “Compared to 1 yr ago, how would you rate your health in general now?”), rated on a 5-point scale ranging from 1 (not better: >2 or better: ≤2)

Hospital Anxiety and Depression Scale (HADS) anxiety subscale	1.57	0	21	More impairment	Puhan MA, Frey M, Büchi S, Schünemann HJ. The minimal important difference of the hospital anxiety and depression scale in patients with chronic obstructive pulmonary disease. Health Qual Life Outcomes. 2008;6:46.	Patients with chronic obstructive pulmonary disease	88	Regression method	Chronic Respiratory Questionnaire (CRQ) emotional function (cutoff: 0.5), Feeling Thermometer (cutoff: 8 points)
Hospital Anxiety and Depression Scale (HADS) anxiety subscale	1.41	0	21	More impairment	Puhan MA, Frey M, Büchi S, Schünemann HJ. The minimal important difference of the hospital anxiety and depression scale in patients with chronic obstructive pulmonary disease. Health Qual Life Outcomes. 2008;6:46.	Patients with chronic obstructive pulmonary disease	88	Regression method	Chronic Respiratory Questionnaire (CRQ) mastery subscale (cutoff: 0.5), Feeling Thermometer (cutoff: 8 points)

Hospital Anxiety and Depression Scale (HADS) anxiety subscale	2	0	21	More impairment	Melanie C, Samantha K, Jane C, Sarah J, Claire N, Amy C, William M. The minimum important difference of the hospital anxiety and depression scale in COPD. European Respiratory Journal. 2014;44(Suppl 58):4829.	Patients with chronic obstructive pulmonary disease	337	ROC curve	Chronic Respiratory Questionnaire (CRQ) fatigue subscale (cutoff: 8 points)
Hospital Anxiety and Depression Scale (HADS) depression subscale	0.5	0	21	More impairment	Lemay KR, Tulloch HE, Pipe AL, Reed JL. Establishing the Minimal Clinically Important Difference for the Hospital Anxiety and Depression Scale in Patients With Cardiovascular Disease. Journal of Cardiopulmonary Rehabilitation and Prevention. 2019;39(6).	Patients with cardiovascular disease undergoing a cardiac rehabilitation program	591	ROC curve	Item 2 of the SF-36 (ie, "Compared to 1 yr ago, how would you rate your health in general now?"), rated on a 5-point scale ranging from 1 (not better: >2 or better: ≤2)

Hospital Anxiety and Depression Scale (HADS) depression subscale	3	0	21	More impairment	Melanie C, Samantha K, Jane C, Sarah J, Claire N, Amy C, William M. The minimum important difference of the hospital anxiety and depression scale in COPD. European Respiratory Journal. 2014;44(Suppl 58):4829.	Patients with chronic obstructive pulmonary disease	294	ROC curve	Chronic Respiratory Questionnaire (CRQ) fatigue subscale (cutoff: 8 points)
King's Brief Interstitial Lung Disease (K-BILD)-Breathlessness and activities domain	6.9	0	100	Less impairment	Sinha A, Patel AS, Siegert RJ, Bajwah S, Maher TM, Renzoni EA, et al. The King's Brief Interstitial Lung Disease (KBILD) questionnaire: an updated minimal clinically important difference. BMJ Open Respir Res. 2019;6(1):e000363.	Patients with Interstitial Lung Disease	57	Average change approach	Forced Vital Cspscity (FVC) (7 to 12%)
King's Brief Interstitial Lung Disease (K-BILD)-Breathlessness and activities domain	8.3	0	100	Less impairment	Sinha A, Patel AS, Siegert RJ, Bajwah S, Maher TM, Renzoni EA, et al. The King's Brief Interstitial Lung Disease (KBILD) questionnaire: an	Patients with Interstitial Lung Disease	57	Average change approach	Global Rating of Change questionnaire (GRCQ) (cutoff: "small change")

					updated minimal clinically important difference. BMJ Open Respir Res. 2019;6(1):e000363.				
King’s Brief Interstitial Lung Disease (K-BILD)-Breathlessness and activities domain	4.4	0	100	Less impairment	Claire MN, Surinder SB, Matthew M, Toby MM, Suhani P, Ruth EB, et al. King's Brief Interstitial Lung Disease questionnaire: responsiveness and minimum clinically important difference. European Respiratory Journal. 2019;54(3):1900281.	Patients with Interstitial Lung Disease and undergoing pulmonary rehabilitation	209	Regression method, ROC curve	Chronic Respiratory Questionnaire (CRQ) dyspnoea subscale (2.5 points), Chronic Respiratory Questionnaire (CRQ) fatigue subscale (2 points), Chronic Respiratory Questionnaire (CRQ) emotion subscale (3.5 points), Chronic Respiratory Questionnaire (CRQ) mastery subscale (2 points), Chronic Respiratory Questionnaire (CRQ) total (10 points), incremental shuttle walk test (ISWT) (44 metres)

King’s Brief Interstitial Lung Disease (K-BILD)-Breathlessness and activities domain	3.6	0	100	Less impairment	Prior TS, Hoyer N, Hilberg O, Shaker SB, Davidsen JR, Bendstrup E. Responsiveness and minimal clinically important difference of SGRQ-I and K-BILD in idiopathic pulmonary fibrosis. Respiratory Research. 2020;21(1):91.	Patients with idiopathic pulmonary fibrosis	124	ROC curve	Global Rating of Change Scales (GRCS) (deteriorated: − 5 to − 2 or improved: 2 to 5), 6-min walk test (6MWD) (deteriorated: Δ6MWD≤ − 28m or improved Δ6MWD≥ 28m)
King’s Brief Interstitial Lung Disease (K-BILD)-Total score	6.1	0	100	Less impairment	Sinha A, Patel AS, Siegert RJ, Bajwah S, Maher TM, Renzoni EA, et al. The King's Brief Interstitial Lung Disease (KBILD) questionnaire: an updated minimal clinically important difference. BMJ Open Respir Res. 2019;6(1):e000363.	Patients with Interstitial Lung Disease	57	Average change approach	Forced Vital Capacity (FVC) (7 to 12%)
King’s Brief Interstitial Lung Disease (K-BILD)-Total score	6.7	0	100	Less impairment	Sinha A, Patel AS, Siegert RJ, Bajwah S, Maher TM, Renzoni EA, et al. The King's Brief Interstitial Lung Disease (KBILD) questionnaire: an updated minimal	Patients with Interstitial Lung Disease	57	Average change approach	Global Rating of Change questionnaire (GRCQ) (cutoff: "small change")

					clinically important difference. BMJ Open Respir Res. 2019;6(1):e000363.				
King's Brief Interstitial Lung Disease (K-BILD)-Total score	3.9	0	100	Less impairment	Claire MN, Surinder SB, Matthew M, Toby MM, Suhani P, Ruth EB, et al. King's Brief Interstitial Lung Disease questionnaire: responsiveness and minimum clinically important difference. European Respiratory Journal. 2019;54(3):1900281.	Patients with Interstitial Lung Disease and undergoing pulmonary rehabilitation	209	Regression method, ROC curve	Chronic Respiratory Questionnaire (CRQ) dyspnoea subscale (2.5 points), Chronic Respiratory Questionnaire (CRQ) fatigue subscale (2 points), Chronic Respiratory Questionnaire (CRQ) emotion subscale (3.5 points), Chronic Respiratory Questionnaire (CRQ) mastery subscale (2 points), Chronic Respiratory Questionnaire (CRQ) total (10 points), incremental shuttle walk test (ISWT) (44 metres)

King's Brief Interstitial Lung Disease (K-BILD)-Total score	4.7	0	100	Less impairment	Prior TS, Hoyer N, Hilberg O, Shaker SB, Davidsen JR, Bendstrup E. Responsiveness and minimal clinically important difference of SGRQ-I and K-BILD in idiopathic pulmonary fibrosis. Respiratory Research. 2020;21(1):91.	Patients with idiopathic pulmonary fibrosis	124	ROC curve	Global Rating of Change Scales (GRCS) (deteriorated: − 5 to − 2 or improved: 2 to 5)
Modified Fatigue Impact Scale (MFIS)	13.8	0	84	More impairment	Kluger BM, Garimella S, Garvan C. Minimal clinically important difference of the Modified Fatigue Impact Scale in Parkinson's disease. Parkinsonism & Related Disorders. 2017;43:101-4.	Patients with Parkinson's disease fatigue randomized to acupuncture or sham	88	Average change approach	Clinical Global Impression of Improvement scale (minimally worse: 5 or minimally improved: 3)
Modified Fatigue Impact Scale (MFIS)	6.25	0	84	More impairment	Rooney S, McFadyen DA, Wood DL, Moffat DF, Paul PL. Minimally important difference of the fatigue severity scale and modified fatigue impact	Patients with multiple sclerosis	365	Regression method	4 items EQ-5D, 24 items Multiple Sclerosis Impact Scale-29 (MSIS-29) (worsened or improved)

					scale in people with multiple sclerosis. Multiple Sclerosis and Related Disorders. 2019;35:158-63.				
Modified Fatigue Impact Scale (MFIS)	7.48*	0	84	More impairment	Santana K, Franca E, Sato J, Silva A, Queiroz M, de Farias J, et al. Non-invasive brain stimulation for fatigue in post-acute sequelae of SARS-CoV-2 (PASC). Brain Stimul. 2023;16(1):100-7.				
Modified Fatigue Impact Scale (MFIS)- Cognitive subscale	6.94	0	40	More impairment	Rooney S, McFadyen DA, Wood DL, Moffat DF, Paul PL. Minimally important difference of the fatigue severity scale and modified fatigue impact scale in people with multiple sclerosis. Multiple Sclerosis and Related Disorders. 2019;35:158-63.	Patients with multiple sclerosis	365	Regression method	3 items EQ-5D, Items 1, 23 items MS Impact Scale-29 (MSIS-29) (worsened or improved)

Modified Fatigue Impact Scale (MFIS)- Physical subscale	6.6	0	36	More impairment	Rooney S, McFadyen DA, Wood DL, Moffat DF, Paul PL. Minimally important difference of the fatigue severity scale and modified fatigue impact scale in people with multiple sclerosis. Multiple Sclerosis and Related Disorders. 2019;35:158-63.	Patients with multiple sclerosis	365	Regression method	3 items EQ-5D, Items 1, 23 items MS Impact Scale-29 (MSIS-29) (worsened or improved)
Modified Medical Research Council dyspnea scale (mMRC dypnea scale)	-0.5 to -0.6	0	4	More impairment	Oliveira A, Machado A, Marques A. Minimal Important and Detectable Differences of Respiratory Measures in Outpatients with AECOPD†. COPD: Journal of Chronic Obstructive Pulmonary Disease. 2018;15(5):479-88	Patients with acute exacerbations of chronic obstructive pulmonary disease (AECOPD) following pharmacologic treatment	44	ROC curve, Regression method	COPD assessment test (≥2 or ≤2)

Multidimensional Dyspnoea Profile (MDP)-Breathing discomfort	0.82	0	10	More impairment	Ekström MP, Bornefalk H, Sköld CM, Janson C, Blomberg A, Bornefalk-Hermansson A, et al. Minimal Clinically Important Differences and Feasibility of Dyspnea-12 and the Multidimensional Dyspnea Profile in Cardiorespiratory Disease. Journal of Pain and Symptom Management. 2020;60(5):968-75.e1.	Patients with cardiorespiratory disease	149	Regression method	Global Impression of Change (GIC)
Multidimensional Dyspnoea Profile (MDP)-Breathing discomfort	0.97*	0	10	More impairment	Romanet C, Wormser J, Fels A, Lucas P, Prudat C, Sacco E, et al. Effectiveness of exercise training on the dyspnoea of individuals with long COVID: A randomised controlled multicentre trial. Ann Phys Rehabil Med. 2023;66(5):101765				

Multidimensional Dyspnoea Profile (MDP)-Emotional response	2.37	0	50	More impairment	Ekström MP, Bornefalk H, Sköld CM, Janson C, Blomberg A, Bornefalk-Hermansson A, et al. Minimal Clinically Important Differences and Feasibility of Dyspnea-12 and the Multidimensional Dyspnea Profile in Cardiorespiratory Disease. Journal of Pain and Symptom Management. 2020;60(5):968-75.e1.	Patients with cardiorespiratory disease	142	Regression method	Global Impression of Change (GIC)
Multidimensional Dyspnoea Profile (MDP)-Emotional response	6.59*	0	50	More impairment	Romanet C, Wormser J, Fels A, Lucas P, Prudat C, Sacco E, et al. Effectiveness of exercise training on the dyspnoea of individuals with long COVID: A randomised controlled multicentre trial. Ann Phys Rehabil Med. 2023;66(5):101765.				

Multidimensional Dyspnoea Profile (MDP)- Sensory dimension	6.32*	0	50	More impairment	Romanet C, Wormser J, Fels A, Lucas P, Prudat C, Sacco E, et al. Effectiveness of exercise training on the dyspnoea of individuals with long COVID: A randomised controlled multicentre trial. Ann Phys Rehabil Med. 2023;66(5):101765.				
Multidimensional Fatigue Inventory-20 (MFI-20)	16.6	20	100	More impairment	Pouchot J, Kherani RB, Brant R, Lacaille D, Lehman AJ, Ensworth S, et al. Determination of the minimal clinically important difference for seven fatigue measures in rheumatoid arthritis. J Clin Epidemiol. 2008;61(7):705-13.	Patients with rheumatoid arthritis	61	Regression method	Patient global impression of fatigue compared to peers
Multidimensional Fatigue Inventory-20 (MFI-20)	11.5	20	100	More impairment	Ewan CG, Jacques P, Rollin B, Raheem BK, Aviña-Zubieta JA, Diane L, et al. Minimal clinically important difference for 7	Patients with systemic lupus erythematosus (SLE)	80	Regression method	Global assessment of fatigue using an 11-point numerical rating scale (NRS) (no fatigue at all: 0 or fatigue as bad as it could be: 10)

					measures of fatigue in patients with systemic lupus erythematosus. The Journal of Rheumatology. 2008;35(4):635.				
Multidimensional Fatigue Inventory-20 (MFI-20)	11.5 to 13.3	20	100	More impairment	Nordin Å, Taft C, Lundgren-Nilsson Å, Dencker A. Minimal important differences for fatigue patient reported outcome measures—a systematic review. BMC Medical Research Methodology. 2016;16(1):62.	Patients with systemic lupus erythematosus (SLE) and rheumatoid arthritis (RA)	141	Regression method	Patient global rating scale and interviews
Multidimensional Fatigue Inventory-20 (MFI-20)	2	20	100	More impairment	Purcell A, Fleming J, Bennett S, Burmeister B, Haines T. Determining the minimal clinically important difference criteria for the Multidimensional Fatigue Inventory in a radiotherapy population. Supportive Care in Cancer. 2010;18(3):307-15.	Patients undergoing radiotherapy	156	Average change approach	Difference in Multidimensional Fatigue Inventory (MFI) scores between pre- and post-radiotherapy intervention

Multidimensional Fatigue Inventory-20 (MFI-20)	7.5*	20	100	More impairment	McIntyre RS, Phan L, Kwan ATH, Mansur RB, Rosenblat JD, Guo Z, et al. Vortioxetine for the treatment of post-COVID-19 condition: a randomized controlled trial. Brain. 2024;147(3):849-57.				
Multidimensional Fatigue Inventory-20 (MFI-20) general fatigue subscale	2	4	20	More impairment	Purcell A, Fleming J, Bennett S, Burmeister B, Haines T. Determining the minimal clinically important difference criteria for the Multidimensional Fatigue Inventory in a radiotherapy population. Supportive Care in Cancer. 2010;18(3):307-15.	Patients undergoing radiotherapy	156	Average change approach	Difference in Multidimensional Fatigue Inventory (MFI) scores between pre- and post-radiotherapy intervention

Multidimensional Fatigue Inventory-20 (MFI-20) mental fatigue subscale	2	4	20	More impairment	Purcell A, Fleming J, Bennett S, Burmeister B, Haines T. Determining the minimal clinically important difference criteria for the Multidimensional Fatigue Inventory in a radiotherapy population. Supportive Care in Cancer. 2010;18(3):307-15.	Patients undergoing radiotherapy	156	Average change approach	Difference in Multidimensional Fatigue Inventory (MFI) scores between pre- and post-radiotherapy intervention
Multidimensional Fatigue Inventory-20 (MFI-20) mental fatigue subscale	10.25 *	4	20	More impairment	McIntyre RS, Phan L, Kwan ATH, Mansur RB, Rosenblat JD, Guo Z, et al. Vortioxetine for the treatment of post-COVID-19 condition: a randomized controlled trial. Brain. 2024;147(3):849-57.				

Multidimensional Fatigue Inventory-20 (MFI-20) physical fatigue subscale	2	4	20	More impairment	Purcell A, Fleming J, Bennett S, Burmeister B, Haines T. Determining the minimal clinically important difference criteria for the Multidimensional Fatigue Inventory in a radiotherapy population. Supportive Care in Cancer. 2010;18(3):307-15.	Patients undergoing radiotherapy	156	Average change approach	Difference in Multidimensional Fatigue Inventory (MFI) scores between pre- and post-radiotherapy intervention
Multidimensional Fatigue Inventory-20 (MFI-20) physical fatigue subscale	8.1*	4	20	More impairment	McIntyre RS, Phan L, Kwan ATH, Mansur RB, Rosenblat JD, Guo Z, et al. Vortioxetine for the treatment of post-COVID-19 condition: a randomized controlled trial. Brain. 2024;147(3):849-57.				
PROMIS 29+2 Profile v2.1 (PROPr) (HRQoL)	0.04	-0.022	1	Less impairment	https://www.proprscore.com/faqs/#:~:text=The%20minimally%20important%20difference%20for%20PROPr%20ha		NR	NR	NR

					s%20not,minimally%20important%20difference%20thresholds%20between%200.03%20and%200.05.
PTSD Symptom Severity (IES-r)	10*	0	88	More impairment	McGregor G, Sandhu H, Bruce J, Sheehan B, McWilliams D, Yeung J, et al. Clinical effectiveness of an online supervised group physical and mental health rehabilitation programme for adults with post-covid-19 condition (REGAIN study): multicentre randomised controlled trial. BMJ. 2024;384:e076506.
Quick Inventory of Depressive Symptomatology -16-item	2.14*	0	27	More impairment	McIntyre RS, Phan L, Kwan ATH, Mansur RB, Rosenblat JD, Guo Z, et al. Vortioxetine for the treatment of post-COVID-19 condition: a randomized

					controlled trial. Brain. 2024;147(3):849-57.				
Quick Inventory of Depressive Symptomatology -16-item	3.5	0	27	More impairment	McIntyre RS, Lipsitz O, Lui LMW, Rodrigues NB, Gill H, Nasri F, et al. The meaningful change threshold as measured by the 16-item quick inventory of depressive symptomatology in adults with treatment-resistant major depressive and bipolar disorder receiving intravenous ketamine. Journal of Affective Disorders. 2021;294:592-6.	Patients with treatment-resistant major depressive and bipolar disorder receiving intravenous ketamine	297	Average change approach	Patient Global Impression - Severity (PGI-S) (improvement or worsening)
Sarcopenia and Quality of Life (SarQol)	5	0	100	Less impairment	Witham MD, Heslop P, Dodds RM, Clegg AP, Hope SV, McDonald C, et al. Performance of the SarQoL quality of life tool in a UK population of older people with probable	Patients 65 and over with self-reported impairment in physical function	125	Average change approach	Patient global impression in change in quality of life (slight improvement or slight worsening)

					sarcopenia and implications for use in clinical trials: findings from the SarcNet registry. BMC Geriatr. 2022;22(1):368.				
SF-36 bodily pain subscale	16.86	0	100	Less impairment	Escobar A, Quintana JM, Bilbao A, Aróstegui I, Lafuente I, Vidaurreta I. Responsiveness and clinically important differences for the WOMAC and SF-36 after total knee replacement. Osteoarthritis Cartilage. 2007;15(3):273-80.	Patients diagnosed with diagnosis of knee osteoarthritis and undergoing total knee replacement	76	Average change approach	Improvement in knee at 6 months and 2 years after the intervention (cutoff: ‘‘somewhat better’’)
SF-36 bodily pain subscale	16.8*	0	100	Less impairment	Zilberman-Itskovich S, Catalogna M, Sasson E, Elman-Shina K, Hadanny A, Lang E, et al. Hyperbaric oxygen therapy improves neurocognitive functions and symptoms of post-COVID condition: randomized controlled trial. Sci				

					Rep. 2022;12(1):11252.				
SF-36 bodily pain subscale	11.1	0	100	Less impairment	Clement ND, Weir D, Deehan D. Meaningful values in the Short Form Health Survey-36 after total knee arthroplasty - an alternative to the EuroQol five-dimension index as a measure for health-related quality of life : minimal clinically important difference, minimal important change, patient-acceptable symptom state thresholds, and responsiveness. Bone Joint Res. 2022;11(7):477-83.	Patients undergoing total knee arthroplasty	375	Average change approach	Patient's impressions of their change in quality of life and satisfaction with knee arthroplasty (no improvement or little improvement)
SF-36 Mental component score	3.2	0	100	Less impairment	Badhiwala JH, Witiw CD, Nassiri F, Akbar MA, Jaja B, Wilson JR, Fehlings MG. Minimum Clinically Important Difference in SF-36 Scores for Use in Degenerative Cervical	Patients undergoing surgery for degenerative cervical myelopathy (DCM)	606	ROC curve	Change in Neck Disability Index (NDI) (unchanged; 7.5≥ΔNDI>−7.5 or slightly improved; −7.5≥ΔNDI>−15)

					Myelopathy. Spine. 2018;43(21).				
SF-36 mental health subscale	4	0	100	Less impairment	Escobar A, Quintana JM, Bilbao A, Aróstegui I, Lafuente I, Vidaurreta I. Responsiveness and clinically important differences for the WOMAC and SF-36 after total knee replacement. Osteoarthritis Cartilage. 2007;15(3):273-80.	Patients diagnosed with diagnosis of knee osteoarthritis and undergoing total knee replacement	65	Average change approach	Improvement in knee at 6 months and 2 years after the intervention (cutoff: ‘‘somewhat better’’)
SF-36 mental health subscale	4.4	0	100	Less impairment	Clement ND, Weir D, Deehan D. Meaningful values in the Short Form Health Survey-36 after total knee arthroplasty - an alternative to the EuroQol five-dimension index as a measure for health-related quality of life : minimal clinically	Patients undergoing total knee arthroplasty	375	Change difference approach	Patient's impressions of their change in quality of life and satisfaction with knee arthroplasty (no improvement or little improvement)

					important difference, minimal important change, patient-acceptable symptom state thresholds, and responsiveness. Bone Joint Res. 2022;11(7):477-83.				
SF-36 Physical component score	3.9	0	100	Less impairment	Badhiwala JH, Witiw CD, Nassiri F, Akbar MA, Jaja B, Wilson JR, Fehlings MG. Minimum Clinically Important Difference in SF-36 Scores for Use in Degenerative Cervical Myelopathy. Spine. 2018;43(21).	Patients undergoing surgery for degenerative cervical myelopathy (DCM)	606	ROC curve	Change in Neck Disability Index (NDI) (unchanged; 7.5≥ΔNDI>−7.5 or slightly improved; −7.5≥ΔNDI>−15)
SF-36 Physical component score	3	0	100	Less impairment	Fu V, Weatherall M, McNaughton H. Estimating the minimal clinically important difference for the Physical Component Summary of the Short Form 36 for patients with stroke. Journal of International Medical Research.	Patients in post-hospital discharge phase of stroke rehabilitation	381	Regression method	Modified version Perceived Health Change (PHC) question (much better: 1 or much worse: 5)

					2021;49(12):03000605211067902.				
SF-36 Physical component score	2.1	0	100	Less impairment	Fu V, Weatherall M, McNaughton H. Estimating the minimal clinically important difference for the Physical Component Summary of the Short Form 36 for patients with stroke. Journal of International Medical Research. 2021;49(12):03000605211067902.	Patients in post-hospital discharge phase of stroke rehabilitation	351	Regression method	Modified version Perceived Health Change (PHC) question (much better: 1 or much worse: 5)
SF-36 physical functioning subscale	13.5	0	100	Less impairment	Clement ND, Weir D, Deehan D. Meaningful values in the Short Form Health Survey-36 after total knee arthroplasty - an alternative to the EuroQol five-dimension index as a measure for health-related quality of life : minimal clinically important difference, minimal important change, patient-acceptable	Patients undergoing total knee arthroplasty	3321	ROC curve	Patient's impressions of their change in quality of life and satisfaction with knee arthroplasty (no improvement or little improvement)

					symptom state thresholds, and responsiveness. Bone Joint Res. 2022;11(7):477-83.				
SF-36 physical functioning subscale	11.56	0	100	Less impairment	Escobar A, Quintana JM, Bilbao A, Aróstegui I, Lafuente I, Vidaurreta I. Responsiveness and clinically important differences for the WOMAC and SF-36 after total knee replacement. Osteoarthritis Cartilage. 2007;15(3):273-80.	Patients diagnosed with diagnosis of knee osteoarthritis and undergoing total knee replacement	76	Average change approach	Improvement in knee at 6 months and 2 years after the intervention (cutoff: ‘‘somewhat better’’)
SF-36 physical functioning subscale	10.4	0	100	Less impairment	Clement ND, Weir D, Deehan D. Meaningful values in the Short Form Health Survey-36 after total knee arthroplasty - an alternative to the EuroQol five-dimension index as a measure for health-related quality of life : minimal clinically important difference, minimal	Patients undergoing total knee arthroplasty	375	ROC curve	Patient's impressions of their change in quality of life and satisfaction with knee arthroplasty (no improvement or little improvement)

					important change, patient-acceptable symptom state thresholds, and responsiveness. Bone Joint Res. 2022;11(7):477-83.				
Transition Dyspnea Index (TDI)	1	-9	9	Less impairment	Witek TJ, Jr., Mahler DA. Minimal important difference of the transition dyspnoea index in a multinational clinical trial. Eur Respir J. 2003;21(2):267-72.	Patients with chronic obstructuve pulmonary disease randomized to tiotropium, salmeterol, or placebo in addition to usual care	997	Average change approach	Physician's Global Evaluation (PGE)
World Health Organization quality of life questionnaire (brief version)	0.51 to 1.27 (varies based on domain)	0	100	Less impairment	Den Oudsten BL, Zijlstra WP, De Vries J. The minimal clinical important difference in the World Health Organization Quality of Life instrument—100. Supportive Care in Cancer. 2013;21(5):1295-301	Female patients with early-stage breast cancer	359	Average change approach	General Health and Overall QOL facet of WHOQOL-100 (small positive change: $2 \le C \le 4$ or small negative change: $-4 \le C \le -2$)

World Health Organization quality of life questionnaire (brief version)	6.66*	0	100	Less impairment	Santana K, Franca E, Sato J, Silva A, Queiroz M, de Farias J, et al. Non-invasive brain stimulation for fatigue in post-acute sequelae of SARS-CoV-2 (PASC). Brain Stimul. 2023;16(1):100-7.

*MID was derived by ½ of standard deviation

Supplement 3. Trial characteristics

Author	Year	Trial name	Registration	Publication status	Funding	Trial design	Country	Recruitment	Method for recruitment	Subtype of long COVID	% Male	Age	Laboratory -confirmed infection	Hospitalized	ICU	Fully vaccinated (according to CDC)	Duration of long COVID	Interventions	Intervention Description	Number of participants randomized
Tosato	2022	NR	NCT04947488	Peer-reviewed	None	Parallel	Italy	July 2021 to not reported	NR	General	34.8	50.5 (median)	100	47.8	8.7	NR	≥2	L-arginine, vitamin C	Patients received 1.66 g of L-arginine and 500 mg of liposomal vitamin C twice daily for 28 days.	25
																		Placebo	NA	25
Romanet	2022	RECOVER	NCT04569266	Peer-reviewed, Preprint	Institutional	Parallel	France	July 2020 to January 2022	Other (Hospital)	Respiratory	61.67	58.1	NR	100	100	NR	NR	Exercise training rehabilitation	Patients received two 60 minute exercise treatment rehabilitation sessions weekly for 10 weeks, consisting of progressively increasing intensity of endurance training and muscle strength training delivered by a pulmonary rehabilitation physiotherapist. During the first assessment, a 6-minute walking test was conducted by the physiotherapist to determine target heart rate. For endurance training, patients initially began at 60–70% of their maximal peak power and the target dyspnoea was 4–6 on the modified Borg scale. The	27

	initial effort lasted 15 minutes and was progressively increased to 45 to 60 minutes of continuous endurance training. Power intensity was tailored to each participant’s progress until the target heart rate and dyspnoea was reached. Muscle strength training consisted of exercises left to the discretion of the physiotherapist and targeted the lower limbs, upper limbs, and core in 4 sets of 6-12 repetitions.	
Standard physiotherapy	Patients received two 30 minute standard physiotherapy sessions weekly for 10 weeks, which were left to the discretion of the general physiotherapist practitioner and according to the patients’s mandatory initial assessment. Sessions involved low-to-moderate intensity aerobic training on an exercise bicycle, ergometer or treadmill, strength training for limbs and trunk muscles, and stretching, balance exercises, electrostimulation and respiratory therapy. Muscle strength training was offered during	33

																			every session consisting of exercises left to the discretion of the physiotherapist and targeted the lower limbs, upper limbs, and core in 4 sets of 6-12 repetitions.	
Gaylis	2022	NR	NCT04678830	Peer-reviewed	Industry, Institutional	Parallel	United States of America	NR	NR	General	NR	48.49	NR	NR	NR	NR	>3	Leronlimab	Patients received 700 mg of leronlimab weekly for 8 weeks.	28
																		Placebo	NA	27
Hansen	2023	NR	NCT04960215	Peer-reviewed	Industry	Cross-over	Denmark	May 2021 to September 2021	Long COVID outpatient clinic	General	25.21	49	NR	15.1	0	84.87	≥3	Coenzyme Q10	Patients received 500 mg of coenzyme Q10 daily for 6 weeks.	59
																		Placebo	NA	62
Oliveira	2023	NR	RBR-7yh559g	Peer-reviewed	NR	Parallel	Brazil	March 2022 to October 2022	NR	General	42.4	52.32	100	38.98	NR	NR	NR	Multicomponent rehabilitation	Patients received two weekly 60 minute multicomponent individual exercise sessions for 12 weeks, consisting of warm-up, resistance, strength, balance, and relaxation.	31
																		Educational orientation	Patients received educational orientations and performed activities of daily living for 12 weeks.	28
Kutashov	2021	NR	NR	Peer-reviewed	None	Parallel	Russia	April 2020 to not reported	Other (Inpatient neurology unit)	Neurological /cognitive	31.98	67.7	100	100	NR	NR	NR	Actovegin	Patients received 400 mg of Actovegin three times daily for 60 days.	222
																		Standard care	NA	222
Samper-Pardo	2023	NR	ISRCTN91104012	Peer-reviewed, Preprint	Government, Institutional	Parallel	Spain	January 2022 to March 2022	General practitioner	General	20	48.28	NR	NR	NR	NR	≥3	ReCOVery APP	Patients continued their treatment as usual overseen by their primary health care professionals and used the APP ReCOVery-telerehabilitation application designed to improve quality of life of	52

																			Long COVID patients. Patients attended one group and two individual sessions over three consecutive weeks which were led by clinical psychologists to promote the adherence to the APP. The APP included six modules: (1) Recommendation to adhere to a Mediterranean diet and to address deficiencies in vitamin D, vitamin B12, complex B, folic acid, and omega-3 fatty acids, (2) Recommendation to improve sleep and rest, (3) Physical exercises with graphical representations, (4) Respiratory physiotherapy with video tutorial, (5) Cognitive stimulation exercises with different difficulty levels, and (6) Participation in community resources.	
																		Standard care	NA	48
Tanashyan	2022	NR	NCT05689827	Peer-reviewed	Industry	Parallel	Russia	April 2022 to not reported	NR	General	25	44.5 (median)	100	NR	NR	NR	NR	Brainmax	Patients received 5 ml (500 mg + 500 mg) of Brainmax daily intramuscularly in the first 10 days, then 2 capsules (250 mg + 250 mg) twice daily for the next 30 days. The supplement contains trimethylhydrazinium	80

																			propionate and ethylmethylhydroxypyridine succinate.	
																		Placebo	NA	80
Zilberman-Itsko	2022	NR	NCT04647656	Peer-reviewed	Institutional	Parallel	Israel	December 2020 to not reported	NR	Neurological /cognitive	39.73	48.1	100	16.44	NR	NR	>3	Hyperbaric oxygen therapy	Patients received hyperbaric oxygen therapy for 40 daily sessions, five sessions per week over a 2 month period. Using a multi-place hyperbaric oxygen chamber system, patients were subjected to 100% oxygen by mask at 2 atmosphere for 90 minutes with five minute air breaks every 20 minutes.	40
																		Placebo	NA	39
Toussaint	2023	NR	NR	Peer-reviewed	Independent donors	Parallel	United States of America	NR	(Social)media	General	12	43.6	NR	NR	NR	NR	NR	Amygdala and insula retraining	Patients attended an introductory online workshop to Amygdala and Insula Retraining (AIR) and received supporting materials online and by mail. AIR involved specialized neuroplasticity techniques, mindfulness-based meditation, alternate nostril breathing, and other lifestyle therapies. Patients received weekly webinars to support their trianing. Over a 3 month period, patients practiced AIR 40 to 60 minutes daily which included the main neuroplasticity processes, a few	50

																			minutes of alternate nostril breathing, and a simple 20-minute mindfulness meditation practice. Patients also practiced abbreviated versions of neuroplaticity techniques throughout the day which took about 30 to 60 seconds.	
																		General education about health and wellbeing	Patients received an online educational program for general health and well-being for 12 weeks which involved general advice on diet, exercise, energy, nutrition, sleep, and other lifestyle interventions. Patients attended weekly webinars, were provided with online resources, and were offered optional online support with a coach trained in the 12 weeks to wellness program.	50
Nambi	2022	NR	NCT04796064	Peer-reviewed	Institutional	Parallel	Saudi Arabia	March 2020 to not reported	Other (Local and government hospitals)	General (sarcopenia)	100	63.65	NR	NR	NR	NR	NR	Low-intensity aerobic training, Strength training	Patients received daily sessions delivered by a physiotherapist of low intensity aerobic training and strength training four days per week for eight weeks, which consisted of a 15 minute warm up, 30 minutes of low intensity aerobic training (40-60% of maximum heart rate), resistance training, and 15 minutes of cool	38

	down. Resistance training was gradually increased as per individual requirements and the major group muscles such as shoulder flexors, shoulder extensors, shoulder abductors, elbow flexors, elbow extensors, hip flexors, hip extensors, knee flexors, knee, extensors, abdominal, and back muscles were trained in three sets of ten repetitions with a rest period of 60 seconds.	
High-intensity aerobic training, Strength training	Patients received daily sessions delivered by a physiotherapist of high intensity aerobic training and strength training four days per week for eight weeks, which consisted of a 15 minute warm up, 30 minutes of low intensity aerobic training (60-80% of maximum heart rate), resistance training, and 15 minutes of cool down. Resistance training was gradually increased as per individual requirements and the major group muscles such as shoulder flexors, shoulder extensors, shoulder abductors, elbow flexors, elbow extensors, hip flexors,	38

																			hip extensors, knee flexors, knee, extensors, abdominal, and back muscles were trained in three sets of ten repetitions with a rest period of 60 seconds.	
Santana	2023	HD-RECOVERY	NCT05289115	Peer-reviewed	Government	Parallel	Brazil	NR	Other (Outpatient clinic)	General	35.71	53.05	100	25.7	NR	NR	NR	Active high-definition transcranial direct current stimulation and rehabilitation	Patients received 3 mA high-definition transcranial direct current stimulation 30 minute sessions targeting the left motor cortex twice weekly for five weeks delivered by researchers, paired with a rehabilitation program. At each session, patients received individually tailored rehabilitation sessions which involved gradual stretching, breathing exercises, resistance training, and educational programs focused on self-management or adapting treatment and coping processes and skills, all in which were led by a physical therapist.	35
																		Placebo and rehabilitation	Patients received individually tailored rehabilitation sessions which involved gradual stretching, breathing exercises, resistance training, and educational programs focused on self-management or	35

																			adapting treatment and coping processes and skills, all in which were led by a physical therapist weekly for five weeks.	
Omarova	2023	NR	NR	Peer-reviewed	None	Parallel	Kazakhstan	March 2022 to not reported	Other (Rehabilitation center)	General	24.4	61.3	NR	NR	NR	NR	NR	Complex rehabilitation methods	Patients received individually tailored rehabilitation prescribed by a physiotherapist, consisting of respiratory gymnastics, massage, myorelaxation, physical therapy, speleotherapy, exercise equipment, aerosol therapy, oxygen cocktail, magnetotherapy, amplipulse, ultrawave frequencies, ultrasound therapy, ultraviolet irradiation, shungite therapy, inhalation, and outdoor walks for 10 to 14 days. The intervention was administered in an in-patient rehabilitation setting.	80
																		Complex rehabilitation methods with acupuncture	Patients received individually tailored rehabilitation prescribed by a physiotherapist, consisting of respiratory gymnastics, massage, myorelaxation, physical therapy, speleotherapy, exercise equipment, aerosol therapy, oxygen cocktail, magnetotherapy,	80

																			amplipulse, ultrawave frequencies, ultrasound therapy, ultraviolet irradiation, shungite therapy, inhalation, and outdoor walks for 10 to 14 days. Patients received individually tailored full-body dry acupuncture treatment, in which 10 points were applied: 9 basic points, and 1 point depending on the specific complaint (shortness of breath, cough, cognitive impairment, increased blood pressure, joint pain, and headache). Each acupuncture session lasted 30 minutes and was limited to only 11 disposable needles, with 7-10 treatment sessions conducted every day for 10 to 14 days. The intervention was administered in an in-patient rehabilitation setting.	
Pleguezu elos	2023	NR	NR	Peer-reviewed	NR	Parallel	Spain	January 2021 to not reported	Other (Hospital)	General	57.25	54.55	100	64.89	33.5 9	NR	>3	Telerehabilitatio n program	Patients performed 3 weekly 60 minute telerehabilitation sessions over 15 weeks, consisting of aerobic and strength training which was supervised by an experienced physiotherapist. Patients warmed up (10 minutes, 40 to 50% heart rate), performed	75

the main workout (50 minutes, 50 to 75% heart rate), and cooled down (10 minutes, resting heart rate). The main part of the session was divided into an aerobic and strength exercise circuit alternating or combining the lower and upper extremities and the core. Specifically, patients performed steps (aerobic), knee elevations, elbow, shoulder, knee, ankle, and neck extension and flexion, abduction-adduction of shoulder, hips, squats, jumps, scissors, calisthenics, and plyometric exercises. Several calisthenic exercises were performed: speed jack, shoulder bridge, superman, lunge, and strong-man flexion. The plyometric exercises were forward step-up and lateral step-up on a box (20–30–40 cm), and counter- movement jump. From weeks 1 to 6, plyometric exercises were performed at regular speed without jumping, weeks 7 to 12 progressed to submaximal jumping with a short eccentric

																			phase before all jumps, and weeks 13 to 15 progressed to maximal jumping with an explosive eccentric-concentric phase. Exercise intensity was monitored by heart rate and perceived exertion (RPE) using the modified BORG scale.	
																		Control	NA	75
Elbanna	2022	NR	NCT04676074	Peer-reviewed	None	Parallel	Egypt	November 2020 to not reported	Other (Hospital)	General	NR	63.55	100	NR	NR	NR	NR	Photobiomodulation Group	Patients received photobiomodulation at one medial and one lateral sites of the calf muscles of both lower limbs three times per week for four weeks. During application, the patients remained prone, their feet out of the plinth, performing a circulatory exercise consisting of dorsiflexion and plantar flexion: two minute intervals of working out were interspersed with one minute rests for 10-minutes for each foot separately.	50
																		Control	NA	50
Alshaimaa	2023	NR	NR	Peer-reviewed	NR	Parallel	Egypt	NR	Other (Hospital)	Respiratory	41.67	45.67	NR	NR	NR	NR	NR	Active cycle of breathing technique and physiotherapy	Active Cycle of Breathing Technique: Patients performed thoracic expansion exercises and forced expiration technique three times a week for 12 weeks. Patients performed thoracic expansion exercises	30

consisting of controlled breathing for 20 to 30 seconds in 3 to 4 repetitions, followed by normal breathing for 20 to 30 seconds or 6 breaths. Aerobic exercise: Patients completed twenty minutes of aerobic exercise on a treadmill three times per week for 12 weeks under the supervision of a physiotherapist. On the 1st day, patients performed 20 minutes of aerobic training (5-minute warming-up, 10-minute training, as well as 5 minutes of cooling-down session). The duration of the aerobic exercises was gradually increased each day based on participants' tolerability. Strengthening exercises: Patients performed active limb exercises followed by progressive muscle strengthening 1 to 3 times a week for 12 weeks. Low-intensity (30-40% of 1RM) to high- intensity (80% of 1RM) exercises were indicated on the Borg scale. The duration of the strengthening exercises were 10 to 45 minutes per session consisting of 3 sets of 8

to 15 repetitions with one minute of resting among sets. Diaphragmatic breathing exercise: Patients performed diaphragmatic breathing exercises in repetitions of 10 with 20 seconds of relaxation in between, 3 times a week for 12 weeks. The patient was directed to take slow, deep breaths through their nose towards their lower belly. Their chest hand should remain still, while their abdomen hand should rise. The patient was instructed to exhale slowly through their nose. Ten times repetition for each exercise was done and 20 seconds of relaxation in between. Pursed-lip abdominal breathing exercise: Patients performed pursed-lip abdominal breathing 3 to 4 times daily for no more than three minutes each time for 12 weeks. The patient sat against an armchair's back, with their arms rested on the chair's armrests or their thighs. The patient closed their mouth and breathed in through their nose for a few

	seconds before gently exhaling through tightly pursed lips for 4 to 6 seconds. The patient formed a large, thin slit with his lips, which delayed their expiration and increased the pressure inside their mouth. In general, the duration of an exhale was double or triple that of an inhale.	
Physiotherapy	Aerobic exercise: Patients completed twenty minutes of aerobic exercise on a treadmill three times per week for 12 weeks under the supervision of a physiotherapist. On the 1st day, patients performed 20 minutes of aerobic training (5-minute warming-up, 10-minute training, as well as 5 minutes of cooling-down session). The duration of the aerobic exercises was gradually increased each day based on participants' tolerability. Strengthening exercises: Patients performed active limb exercises followed by progressive muscle strengthening 1 to 3 times a week for 12 weeks. Low-intensity (30-40% of 1RM) to high- intensity (80% of 1RM) exercises were	30

indicated on the Borg scale. The duration of the strengthening exercises were 10 to 45 minutes per session consisting of 3 sets of 8 to 15 repetitions with one minute of resting among sets. Diaphragmatic breathing exercise: Patients performed diaphragmatic breathing exercises in repetitions of 10 with 20 seconds of relaxation in between, 3 times a week for 12 weeks. The patient was directed to take slow, deep breaths through their nose towards their lower belly. Their chest hand should remain still, while their abdomen hand should rise. The patient was instructed to exhale slowly through their nose. Ten times repetition for each exercise was done and 20 seconds of relaxation in between. Pursed-lip abdominal breathing exercise: Patients performed pursed-lip abdominal breathing 3 to 4 times daily for no more than three minutes each time for 12 weeks. The patient sat against an armchair's back, with

																			their arms rested on the chair's armrests or their thighs. The patient closed their mouth and breathed in through their nose for a few seconds before gently exhaling through tightly pursed lips for 4 to 6 seconds. The patient formed a large, thin slit with his lips, which delayed their expiration and increased the pressure inside their mouth. In general, the duration of an exhale was double or triple that of an inhale.	
McNarry	2022	NR	NR	Peer-reviewed	Government	Parallel	United Kingdom	NR	(Social)media, Other (Hospital, Online support groups)	Respiratory	12.84	46.6	NR	NR	NR	NR	NR	Inspiratory Muscle training	Patients performed 3 unsupervised weekly inspiratory muscle training sessions (on non consecutive days) for 8 weeks. Sustained maximal inspiratory pressure (SMIP) was determined prior to each session and >80% SMIP was required to be maintained during training. Each session had a maximum duration of 20 minutes and consisted of up to six blocks of six inspirations, with the rest periods between each inspiration progressively decreasing from 40 to 10 seconds with each block. Patients completed as	224

																			many inspirations as possible prior to failure.	
																		Standard care	NA	57
Mooren	2023	NR	NCT06016192	Peer-reviewed	NR	Parallel	Germany	August 2021 to not reported	Other (Inpatient rehabilitation clinic)	General	61.8	49.3	100	28.2	NR	NR	≥3	Continuous Training	Patients performed continuous bicycle ergometer training 3 to 5 times per week for 4 to 6 weeks. Training was prescribed by clinicians, scheduled by therapy management, and implemented in groups (4 to 6 patients) by therapists. A standard workload of 50% of maximal workload was applied and each session lasted 18 minutes, with a gradual increase (ramp) at session start until training load was reached. In addition, patients received other individualized physical therapies such as (aerobic) group exercise, medical training therapy, aqua fitness, terrain training/walking, and circuit training.	69
																		Interval Training	Patients performed interval bicycle ergometer training 3 to 5 times per week for 4 to 6 weeks. Training was prescribed by clinicians, scheduled by therapy management, and implemented in groups (4 to 6 patients) by therapists. The workload was 60% at	70

																			load (100 seconds) and 30% during recovery (48 seconds) and each session lasted 18 minutes, with a gradual increase (ramp) at session start until training load was reached. In addition, patients received other individualized physical therapies such as (aerobic) group exercise, medical training therapy, aqua fitness, terrain training/walking, and circuit training.	
McIntyre	2023	NR	NCT05047952	Peer-reviewed	Industry	Parallel	Canada	November 2021 to January 2023	Long COVID outpatient clinic, (Social)media	General	34.23	44.29	NR	NR	NR	NR	≥2	Vortioxetine	Patients aged 18 to 65 received vortioxetine at 10 mg daily during weeks 1 and 2 and 20 mg daily from weeks 3 to 8. Patients aged 65 or older received vortioxetine at 5 mg daily during weeks 1 and 2 and 10 mg daily from weeks 3 to 8. Patients unable to tolerate higher doses, down titration to the index dose was permitted.	75
																		Placebo	NA	74
Ryabokon	2023	NR	NR	Peer-reviewed	NR	Parallel	Russia	May 2020 to November 2020	NR	General	19	51.4	NR	NR	NR	NR	NR	Active hydrogen therapy and standard therapy	Patients received standard therapy according to the protocol for managing patients with CFS: physiotherapy and medication therapy with drugs containing magnesium, B vitamins	30

																			and L-carnitine for 10 days. Patients received active hydrogen therapy (through a nasal cannula connected to the device "SUISONIA") daily for 10 days for 90 minutes.	
																		Standard therapy	Patients received standard therapy according to the protocol for managing patients with CFS: physiotherapy and medication therapy with drugs containing magnesium, B vitamins and L-carnitine for 10 days.	30
Kuut	2023	ReCOVer	NL8947	Peer-reviewed	Government	Parallel	Netherlands	NR	Long COVID outpatient clinic, Other (Self-referral, Hospital)	General	27.19	45.85	100	11.4	NR	100	NR	Cognitive behavioural therapy	Patients received cognitive behavioural therapy through a program called Fit after COVID for 17 weeks, adapted based on existing cognitive behavioral therapy protocols for severe fatigue in long-term medical conditions. The 7 perpetuating factors addressed were (1) a disrupted sleep–wake pattern; (2) unhelpful beliefs about fatigue; (3) a low or unevenly distributed activity level; (4) perceived low social support; (5) problems with psychological processing of COVID-19; (6) fears and worries regarding	57

COVID-19; and (7) poor coping with pain. Patients could access treatment modules on an online platform, but face-to-face contact was also available for those patients who were unable or unwilling to use the internet-based format. All therapists were psychologists trained in the treatment protocol during a 4-day course and supervised biweekly by experienced clinical psychologists to ensure treatment integrity. The treatment modules were as follows: Goal setting: Psychoeducation regarding the cognitive-behavioral model of post-infectious fatigue following COVID-19. Patients set treatment goals in concrete activities which will be performed when the fatigue is alleviated. Sleep-wake pattern (Instrument and cut off score: Sleep diary ISI ≥ 10): Targeted a disrupted sleep-wake pattern. Patients established a regular sleep-wake pattern and follow sleep-hygiene practices. Patients were encouraged to stop

sleeping or lying down at daytime. Helpful thinking (Instrument and cut off score: J-FCS ≥ 16, IMQ ≥ 30, SES ≤ 19): Targeted unhelpful cognitions regarding fatigue. Patients learned to identify unhelpful thoughts and replace them with helpful thoughts and increase their self-efficacy. Patients learned to redirect their attention away from fatigue. Social support (optional module) (Instrument and cut off score: SSL-I ≥ 14, SSL-D ≥ 50): Targeted low perceived social support and negative interactions. Patients learned how to communicate with significant others about their fatigue, be assertive and adapt expectations about their environment. Graded activity: Targeted a low or fluctuating physical activity pattern. Patients with low activity pattern started with a gradual increase of their daily physical activity. Patients with a relative active activity pattern learned first to evenly distribute their activities during the day and then subsequently gradually

increased their daily activity. Processing COVID-19 (optional module) (Instrument and cut off score: IES subscales intrusion and/or avoidance ≥ 10): Targeted emotional problems of patients who did not process COVID-19. Patients were helped to process negative experiences of their illness. Fears and worries regarding COVID-19 (optional module) (Instrument and cut off score: COWS ≥ 10): Targeted excessive fears and worries regarding COVID-19. Patients recorded what the content of their fears and worries were regarding COVID-19. Patients learned to formulate helpful thoughts and to distance themselves from their anxious thoughts. Coping with pain (optional module) (Instrument and cut off score: SF-36, pain subscale ≤ 40): Targeted unhelpful cognitions with respect to pain. Patients were helped to deal with pain in such a way that it does not limit them during the gradual increase of

																			activities. Realizing goals: Patients made an action plan to realize their treatment goals, like increasing social and mental activities. Patients learned about the difference between severe fatigue and normal fatigue. Patients learned to let go of the regular sleep-wake pattern and even distribution of activities. Patients evaluated their progress. The intervention was personalized in two ways: First, content of the graded activity module was adapted based on the patient's scores on the baseline assessment (T0) and based on data provided with an actigraph, a device worn at the wrist to assess physical activity and discerning a low active and relative active pattern. Second, of the optional modules only those are selected that apply to the patient, based on T0 scores as well as information collected by the therapist during the intake session.	
																		Standard care	NA	57
Sizyakina	2023	NR	NR	Peer-reviewed	None	Parallel	Russia	NR	NR	General	50	54	NR	NR	NR	NR	NR	Licopid	Patients received 1 mg of glucosaminylmuramyl dipeptide twice daily for	30

																			10 days, followed by a break for 20 days.	
																		Control	NA	30
Lau	2023	RECOVERY	NCT04950803	Peer-reviewed	Government, Not-for-profit foundation	Parallel	China	June 2021 to August 2022	Other (Hospital)	General	34.56	49.45	100	30.67	NR	69.11	≥1	Microbiome immunity formula	Patients recieved a sachet containing 10 billion colony-forming units of three bacterial strains (ie, B adolescentis, B bifidum, and B longum) and three prebiotic compounds (ie, galacto-oligosaccharides, xylo-oligosaccharides, and resistant dextrin) twice daily for 6 months.	232
																		Placebo	NA	231
McGregor	2024	REGAIN	ISRCTN11466448	Peer-reviewed	Government	Parallel	United Kingdom	January 2021 to July 2022	Other (Post via NHS trusts, NHS digital mailout, self-referral)	General	47.86	56.1	NR	100	34.36	NR	NR	REGAIN	Patients recieved an online group rehabilitation program, supported by a workbook for 8 weeks. Patients initially received a 30 to 60 minute, virtual, one-to-one consultation with a practioner to discuss their medical history and ways in which physical and mental health recovery could be supported. Patients attended weekly physiologist/physiotherapist led live online group exercise sessions and six live online group psychological support sessions led by health psychologists (one hour each). Topics of discussion during psychological support	298

	sessions, which was also supported by short introductory videos, included motivation, fear avoidance, activity pacing, managing emotions and set-backs, sleep and fatigue, and stress and anxiety management. Patients were offered the opportunity to share their own experiences with the group. Patients recieved on demand exercise videos varying in duration and intensity from simple breathing exercises, pilates, yoga, light seated activity, and upright moderate to high intensity exercise.	
Standard care	Patients received a 30 minute online consultation with a practitoner and a trial booklet, both consisiting of generic information and advice regarding recovery from COVID-19.	287

Supplement 4. Risk of bias of trials reporting on pharmacologic interventions

Trial	Outcome	Measure	Randomization	Deviations from the intended intervention	Missing outcome data	Measurement of outcome	Selection of the reported results
		**Leronlimab vs. Placebo**					
Gaylis 2022	Cognitive function	Ad-hoc symptom severity score (0-3)	Probably low risk of bias	Probably low risk of bias	Low risk of bias	Probably low risk of bias	Probably low risk of bias
Gaylis 2022	Dyspnea	Ad-hoc symptom severity score (0-3)	Probably low risk of bias	Probably low risk of bias	Low risk of bias	Probably low risk of bias	Probably low risk of bias
Gaylis 2022	Fatigue	Ad-hoc symptom severity score (0-3)	Probably low risk of bias	Probably low risk of bias	Low risk of bias	Probably low risk of bias	Probably low risk of bias
Gaylis 2022	Mental health	Ad-hoc symptom severity score (0-3)	Probably low risk of bias	Probably low risk of bias	Low risk of bias	Probably low risk of bias	Probably low risk of bias
Gaylis 2022	Post-exertional malaise	Ad-hoc symptom severity score (0-3)	Probably low risk of bias	Probably low risk of bias	Low risk of bias	Probably low risk of bias	Probably low risk of bias
		**Actovegin vs. Usual care**					
Kutashov 2022	Cognitive function	Montreal Cognitive Assessment test (MoCA)	Probably high risk of bias	Probably high risk of bias	Low risk of bias	High risk of bias	Probably low risk of bias
Kutashov 2022	Fatigue	Multidimensional Fatigue Inventory-20 (MFI-20)	Probably high risk of bias	Probably high risk of bias	Low risk of bias	High risk of bias	Probably low risk of bias
		**Vortioxetine vs. Placebo**					
McIntyre 2023	Cognitive function	Digital Symbol Substitution Test	Probably low risk of bias	Low risk of bias	Low risk of bias	Low risk of bias	Low risk of bias
McIntyre 2023	Mental health	Quick Inventory of Depressive Symptomatology-16-item (QIDS-SR16)	Probably low risk of bias	Low risk of bias	Low risk of bias	Low risk of bias	Low risk of bias
McIntyre 2023	Quality of life	World Health Organisation-5 Well-Being Index (WHO-5)	Probably low risk of bias	Low risk of bias	Low risk of bias	Low risk of bias	Low risk of bias
		**Glucosaminyl muramyl dipeptide vs. Usual care**					
Sizyakina 2023	Mental health	Hospital Anxiety and Depression Scale (HADS) anxiety subscale	Probably high risk of bias	Probably high risk of bias	Probably low risk of bias	High risk of bias	Probably low risk of bias
Sizyakina 2023	Mental health	Hospital Anxiety and Depression Scale (HADS) depression subscale	Probably high risk of bias	Probably high risk of bias	Probably low risk of bias	High risk of bias	Probably low risk of bias
Sizyakina 2023	Mental health	SF-36 Mental component score	Probably high risk of bias	Probably high risk of bias	Probably low risk of bias	High risk of bias	Probably low risk of bias
Sizyakina 2023	Mental health	SF-36 mental health subscale	Probably high risk of bias	Probably high risk of bias	Probably low risk of bias	High risk of bias	Probably low risk of bias
Sizyakina 2023	Pain	SF-36 bodily pain subscale	Probably high risk of bias	Probably high risk of bias	Probably low risk of bias	High risk of bias	Probably low risk of bias
Sizyakina 2023	Physical function	SF-36 Physical component score	Probably high risk of bias	Probably high risk of bias	Probably low risk of bias	High risk of bias	Probably low risk of bias
Sizyakina 2023	Physical function	SF-36 physical functioning subscale	Probably high risk of bias	Probably high risk of bias	Probably low risk of bias	High risk of bias	Probably low risk of bias

Legend:
- Low risk of bias (green)
- Probably low risk of bias (yellow)
- Probably high risk of bias (pink)
- High risk of bias (red)

Supplement 5. Risk of bias of trials reporting on physical activity and rehabilitative interventions

Trial	Outcome	Measure	Randomization	Deviations from the intended intervention	Missing outcome data	Measurement of outcome	Selection of the reported results
		**Active cycle of breathing technique, Physiotherapy vs. Physiotherapy**					
Alshaimaa 2023	Fatigue	Fatigue Assessment Scale-10 (FAS-10)	Probably high risk of bias	Probably high risk of bias	Probably low risk of bias	Probably low risk of bias	Probably low risk of bias
		**Inspiratory muscle training vs. Usual care**					
McNarry 2023	Dyspnea	King's Brief Interstitial Lung Disease (K-BILD)- Breathlessness and activities domain	Probably low risk of bias	Probably high risk of bias	High risk of bias	High risk of bias	Probably low risk of bias
McNarry 2023	Dyspnea	Transition Dyspnea Index (TDI)	Probably low risk of bias	Probably high risk of bias	High risk of bias	High risk of bias	Probably low risk of bias
McNarry 2023	Quality of life	King's Brief Interstitial Lung Disease (K-BILD)- Total score	Probably low risk of bias	Probably high risk of bias	High risk of bias	High risk of bias	Probably low risk of bias
		**Continuous aerobic exercise vs. Intermittent aerobic exercise**					
Mooren 2023	Fatigue	Multidimensional Fatigue Inventory-20 (MFI-20)	Probably high risk of bias	Probably low risk of bias	High risk of bias	Probably low risk of bias	Low risk of bias
Mooren 2023	Fatigue	Multidimensional Fatigue Inventory-20 (MFI-20) mental fatigue subscale	Probably high risk of bias	Probably low risk of bias	High risk of bias	Probably low risk of bias	Low risk of bias
Mooren 2023	Fatigue	Multidimensional Fatigue Inventory-20 (MFI-20) physical fatigue subscale	Probably high risk of bias	Probably low risk of bias	High risk of bias	Probably low risk of bias	Low risk of bias
Mooren 2023	Mental health	Hospital Anxiety and Depression Scale (HADS) anxiety subscale	Probably high risk of bias	Probably low risk of bias	High risk of bias	Probably low risk of bias	Probably low risk of bias
Mooren 2023	Mental health	Hospital Anxiety and Depression Scale (HADS) depression subscale	Probably high risk of bias	Probably low risk of bias	High risk of bias	Probably low risk of bias	Probably low risk of bias
Mooren 2023	Mental health	SF-36 Mental component score	Probably high risk of bias	Probably low risk of bias	High risk of bias	Probably low risk of bias	Probably low risk of bias
Mooren 2023	Physical function	SF-36 Physical component score	Probably high risk of bias	Probably low risk of bias	High risk of bias	Probably low risk of bias	Probably low risk of bias
Mooren 2023	Quality of life	World Health Organisation-5 Well-Being Index (WHO-5)	Probably high risk of bias	Probably low risk of bias	High risk of bias	Probably low risk of bias	Low risk of bias
		**Low-intensity aerobic training, Strength training vs. High-intensity aerobic training, Strength training**					
Nambi 2022	Quality of life	Sarcopenia and Quality of Life (SarQol)	Probably low risk of bias	Probably low risk of bias	Low risk of bias	Probably low risk of bias	Probably low risk of bias
		**In-patient rehabilitation, Physiotherapy vs. In-patient rehabilitation, Physiotherapy, Acupuncture**					
Omarova 2022	Dyspnea	Modified Medical Research Council dyspnea scale (mMRC dypnea scale)	Low risk of bias	Probably high risk of bias	Low risk of bias	High risk of bias	Probably low risk of bias
		**Physiotherapy, Multicomponent exercise of progressively increasing intensity vs. Physiotherapy**					
Romanet 2022 (RECOVER)	Dyspnea	Multidimensional Dyspnoea Profile (MDP)	Low risk of bias	Probably low risk of bias	Low risk of bias	Probably low risk of bias	Low risk of bias
Romanet 2022 (RECOVER)	Dyspnea	Multidimensional Dyspnoea Profile (MDP)- Breathing discomfort	Low risk of bias	Probably low risk of bias	Low risk of bias	Probably low risk of bias	Low risk of bias
Romanet 2022 (RECOVER)	Dyspnea	Multidimensional Dyspnoea Profile (MDP)- Emotional response	Low risk of bias	Probably low risk of bias	Low risk of bias	Probably low risk of bias	Low risk of bias
Romanet 2022 (RECOVER)	Dyspnea	Multidimensional Dyspnoea Profile (MDP)- Sensory dimension	Low risk of bias	Probably low risk of bias	Low risk of bias	Probably low risk of bias	Low risk of bias
Romanet 2022 (RECOVER)	Mental health	SF-12 Mental component score	Low risk of bias	Probably low risk of bias	Low risk of bias	Probably low risk of bias	Probably low risk of bias
Romanet 2022 (RECOVER)	Physical function	SF-12 Physical component score	Low risk of bias	Probably low risk of bias	Low risk of bias	Probably low risk of bias	Probably low risk of bias
Romanet 2022 (RECOVER)	Quality of life	Short-Form Survey (SF-12)- Total score	Low risk of bias	Probably low risk of bias	Low risk of bias	Probably low risk of bias	Low risk of bias

Low risk of bias
Probably low risk of bias
Probably high risk of bias
High risk of bias

Supplement 6. Risk of bias of trials reporting on behavioral interventions

Trial	Outcome	Measure	Randomization	Deviations from the intended intervention	Missing outcome data	Measurement of outcome	Selection of the reported results
**CBT vs. Usual care**							
Kuut 2023(ReCOVer)	Cognitive function	Checklist Individual Strength (CIS) concentration problems subscale	Low	Probably high	Low	High	Low
Kuut 2023(ReCOVer)	Fatigue	Checklist Individual Strength (CIS) fatigue subscale	Low	Probably high	Low	High	Low
Kuut 2023(ReCOVer)	Physical function	SF-36 physical functioning subscale	Low	Probably high	Low	High	Low
Kuut 2023(ReCOVer)	Recovery or important improv	NA	Low	Probably high	Low	High	Low
Kuut 2023(ReCOVer)	Serious adverse events	NA	Low	Probably high	Low	High	Low
**Telerehabilitation app vs. Usual care**							
Samper-Pardo 2023	Cognitive function	Montreal Cognitive Assessment test (MoCA)	Probably high	Probably high	Probably low	High	Low
Samper-Pardo 2023	Mental health	SF-36 Mental component score	Probably high	Probably high	Probably low	High	Probably low
Samper-Pardo 2023	Mental health	Hospital Anxiety and Depression Scale (HADS)	Probably high	Probably high	Probably low	High	Low
Samper-Pardo 2023	Mental health	Brief Symptom Inventory-18 (BSI-18)	Probably high	Probably high	Probably low	High	Low
Samper-Pardo 2023	Physical function	Physical function	Probably high	Probably high	Probably low	High	Probably low
Samper-Pardo 2023	Serious adverse events	NA	Probably high	Probably high	Probably low	High	Probably low
**Amygdala and insula retraining vs. Education related to self-management**							
Toussaint 2023	Fatigue	Multidimensional Fatigue Inventory-20 (MFI-20) general fatigue subscale	Probably high	Probably low	High	Probably low	Probably low

Legend:
- Low risk of bias (green)
- Probably low risk of bias (yellow)
- Probably high risk of bias (pink)
- High risk of bias (red)

Supplement 7. Risk of bias of trials reporting on dietary supplements and other dietary interventions

Trial	Outcome	Measure	Randomization	Deviations from the intended intervention	Missing outcome data	Measurement of outcome	Selection of the reported results
**Coenzyme Q10 vs. Placebo**							
Hansen 2023	Quality of life	EQ-5D health index	Low risk of bias	Low risk of bias	Low risk of bias	Low risk of bias	Probably low risk of bias
Hansen 2023	Serious adverse events	NA	Low risk of bias	Low risk of bias	Low risk of bias	Low risk of bias	Low risk of bias
**Probiotics and prebiotics vs. Placebo**							
Lau 2023 (RECOVERY)	Quality of life	Visual analogue scale (VAS) (1-100)	Low risk of bias	Low risk of bias	Probably low risk of bias	Low risk of bias	Probably low risk of bias
Lau 2023 (RECOVERY)	Recovery or important improvement	NA	Low risk of bias	Low risk of bias	Probably low risk of bias	Low risk of bias	Probably high risk of bias
Lau 2023 (RECOVERY)	Serious adverse events	NA	Low risk of bias	Low risk of bias	Probably low risk of bias	Low risk of bias	Probably low risk of bias
**Brainmax vs. Placebo**							
Tanashyan 2022	Cognitive function	Montreal Cognitive Assessment test (MoCA)	Probably low risk of bias	Low risk of bias	Low risk of bias	Low risk of bias	Low risk of bias
Tanashyan 2022	Fatigue	Fatigue Assessment Scale-10 (FAS-10)	Probably low risk of bias	Low risk of bias	Low risk of bias	Low risk of bias	Low risk of bias
Tanashyan 2022	Fatigue	Multidimensional Fatigue Inventory-20 (MFI-20)	Probably low risk of bias	Low risk of bias	Low risk of bias	Low risk of bias	Low risk of bias
Tanashyan 2022	Fatigue	Multidimensional Fatigue Inventory-20 (MFI-20) asthenia subscale	Probably low risk of bias	Low risk of bias	Low risk of bias	Low risk of bias	Low risk of bias
Tanashyan 2022	Mental health	Beck Anxiety Inventory	Probably low risk of bias	Low risk of bias	Low risk of bias	Low risk of bias	Low risk of bias
Tanashyan 2022	Recovery or important improvement	NA	Probably low risk of bias	Low risk of bias	Low risk of bias	Low risk of bias	Probably low risk of bias
Tanashyan 2022	Serious adverse events	NA	Probably low risk of bias	Low risk of bias	Low risk of bias	Low risk of bias	Probably low risk of bias
**L-arginine, vitamin C vs. Placebo**							
Tosato 2022	Recovery or important improvement	NA	Probably high risk of bias	Probably high risk of bias	Low risk of bias	Low risk of bias	Probably low risk of bias
Tosato 2022	Serious adverse events	NA	Probably high risk of bias	Probably high risk of bias	Low risk of bias	Probably high risk of bias	Probably low risk of bias

Low risk of bias
Probably low risk of bias
Probably high risk of bias
High risk of bias

Supplement 8. Risk of bias of trials reporting on medical devices and technologies

Trial	Outcome	Measure	Randomization	Deviations from the intended intervention	Missing outcome data	Measurement of outcome	Selection of the reported results
**Photobiomodulation vs. Placebo**							
Elbanna 2022	Fatigue	Fatigue Severity Scale (FSS)	Probably high risk of bias	Low risk of bias	Low risk of bias	Low risk of bias	Probably low risk of bias
Elbanna 2022	Physical function	Katz Index of Independence in Tasks of Everyday Living	Probably high risk of bias	Low risk of bias	Low risk of bias	Low risk of bias	Probably low risk of bias
**Transcranial direct current stimulation, Physiotherapy, Education related to activities of daily living vs. Physiotherapy, Education related to self-management**							
Santana 2023 (HD-RECOVERY)	Fatigue	Modified Fatigue Impact Scale (MFIS)	Probably low risk of bias	Low risk of bias	Probably low risk of bias	Low risk of bias	Low risk of bias
Santana 2023 (HD-RECOVERY)	Fatigue	Modified Fatigue Impact Scale (MFIS)- Cognitive subscale	Probably low risk of bias	Low risk of bias	Probably low risk of bias	Low risk of bias	Low risk of bias
Santana 2023 (HD-RECOVERY)	Fatigue	Modified Fatigue Impact Scale (MFIS)- Physical subscale	Probably low risk of bias	Low risk of bias	Probably low risk of bias	Low risk of bias	Low risk of bias
Santana 2023 (HD-RECOVERY)	Mental health	Hamilton Anxiety Rating Scale (HAM-A)	Probably low risk of bias	Low risk of bias	Probably low risk of bias	Low risk of bias	Low risk of bias
Santana 2023 (HD-RECOVERY)	Pain	McGill Pain Questionnaire	Probably low risk of bias	Low risk of bias	Probably low risk of bias	Low risk of bias	Low risk of bias
Santana 2023 (HD-RECOVERY)	Quality of life	World Health Organization quality of life questionnaire (brief version)	Probably low risk of bias	Low risk of bias	Probably low risk of bias	Low risk of bias	Low risk of bias
Santana 2023 (HD-RECOVERY)	Recovery or important improvement	NA	Probably low risk of bias	Low risk of bias	Probably low risk of bias	Low risk of bias	Probably low risk of bias
Santana 2023 (HD-RECOVERY)	Serious adverse events	NA	Probably low risk of bias	Low risk of bias	Probably low risk of bias	Low risk of bias	Probably low risk of bias
**Hyperbaric oxygen therapy vs. Placebo**							
Zilberman 2022	Cognitive function	NeuroTrax computerized cognitive testing battery- Global score	Probably low risk of bias	Low risk of bias	Low risk of bias	Low risk of bias	Probably low risk of bias
Zilberman 2022	Mental health	Brief Symptom Inventory-18 (BSI-18)	Probably low risk of bias	Low risk of bias	Low risk of bias	Low risk of bias	Probably low risk of bias
Zilberman 2022	Mental health	Brief Symptom Inventory-18 (BSI-18) anxiety subscale	Probably low risk of bias	Low risk of bias	Low risk of bias	Low risk of bias	Probably low risk of bias
Zilberman 2022	Mental health	Brief Symptom Inventory-18 (BSI-18) depression subscale	Probably low risk of bias	Low risk of bias	Low risk of bias	Low risk of bias	Probably low risk of bias
Zilberman 2022	Mental health	Brief Symptom Inventory-18 (BSI-18) somatization subscale	Probably low risk of bias	Low risk of bias	Low risk of bias	Low risk of bias	Probably low risk of bias
Zilberman 2022	Mental health	SF-36 mental health subscale	Probably low risk of bias	Low risk of bias	Low risk of bias	Low risk of bias	Probably low risk of bias
Zilberman 2022	Pain	Brief Pain Inventory (BPI) pain severity subscale	Probably low risk of bias	Low risk of bias	Low risk of bias	Low risk of bias	Probably low risk of bias
Zilberman 2022	Pain	SF-36 bodily pain subscale	Probably low risk of bias	Low risk of bias	Low risk of bias	Low risk of bias	Probably low risk of bias
Zilberman 2022	Physical function	SF-36 physical functioning subscale	Probably low risk of bias	Low risk of bias	Low risk of bias	Low risk of bias	Probably low risk of bias

Low risk of bias
Probably low risk of bias
Probably high risk of bias
High risk of bias

Supplement 9: Risk of bias of trials reporting on combination therapies

Trial	Outcome	Measure	Randomization	Deviations from the intended intervention	Missing outcome data	Measurement of outcome	Selection of the reported results
**Physical and mental health rehabilitation vs. Usual care**							
McGregor 2024 (REGAIN)	Cognitive function	Cognitive Function (PROMIS Neuro-QoL)	Low risk of bias	Probably high risk	High risk of bias	High risk of bias	Low risk of bias
McGregor 2024 (REGAIN)	Cognitive function	PROMIS (patient-reported outcomes measurement information system)- Cognitive function abilities subscore	Low risk of bias	Probably high risk	High risk of bias	High risk of bias	Low risk of bias
McGregor 2024 (REGAIN)	Dyspnea	PROMIS (patient-reported outcomes measurement information system) Dyspnoea Severity Short Form	Low risk of bias	Probably high risk	High risk of bias	High risk of bias	Low risk of bias
McGregor 2024 (REGAIN)	Fatigue	PROMIS (patient-reported outcomes measurement information system)- Fatigue subscore	Low risk of bias	Probably high risk	High risk of bias	High risk of bias	Low risk of bias
McGregor 2024 (REGAIN)	Mental health	Hospital Anxiety and Depression Scale (HADS) anxiety subscale	Low risk of bias	Probably high risk	High risk of bias	High risk of bias	Low risk of bias
McGregor 2024 (REGAIN)	Mental health	Hospital Anxiety and Depression Scale (HADS) depression subscale	Low risk of bias	Probably high risk	High risk of bias	High risk of bias	Low risk of bias
McGregor 2024 (REGAIN)	Mental health	PROMIS (patient-reported outcomes measurement information system)- Emotional distress – Anxiety subscore	Low risk of bias	Probably high risk	High risk of bias	High risk of bias	Low risk of bias
McGregor 2024 (REGAIN)	Mental health	PROMIS (patient-reported outcomes measurement information system)- Emotional distress – Depression subscore	Low risk of bias	Probably high risk	High risk of bias	High risk of bias	Low risk of bias
McGregor 2024 (REGAIN)	Pain	PROMIS (patient-reported outcomes measurement information system)- Pain intensity subscore	Low risk of bias	Probably high risk	High risk of bias	High risk of bias	Low risk of bias
McGregor 2024 (REGAIN)	Physical function	PROMIS (patient-reported outcomes measurement information system)- Physical function abilities subscore	Low risk of bias	Probably high risk	High risk of bias	High risk of bias	Low risk of bias
McGregor 2024 (REGAIN)	Quality of life	EQ5D-5L Index Score	Low risk of bias	Probably high risk	High risk of bias	High risk of bias	Low risk of bias
McGregor 2024 (REGAIN)	Quality of life	EQ5D-5L Visual Anlogue Scale (0-100 cm)	Low risk of bias	Probably high risk	High risk of bias	High risk of bias	Low risk of bias
McGregor 2024 (REGAIN)	Quality of life	PROMIS 29+2 Profile v2.1 (PROPr) (HRQoL)	Low risk of bias	Probably high risk	High risk of bias	High risk of bias	Low risk of bias
McGregor 2024 (REGAIN)	Recovery or important improvement	NA	Low risk of bias	Probably high risk	High risk of bias	High risk of bias	Probably low risk
McGregor 2024 (REGAIN)	Serious adverse events	NA	Low risk of bias	Probably high risk	High risk of bias	High risk of bias	Probably low risk

Legend:
- Low risk of bias
- Probably low risk
- Probably high risk
- High risk of bias

Supplement 10: Summary of findings table comparing Vortioxetine and Placebo

Patients: Patients with long COVID
Intervention: Vortioxetine
Comparator: Placebo

Outcome	Trials (patients)	Relative Risk (95% CI)	Absolute effect (per 1000 patients)	Certainty of the evidence	Plain language summary
Mental health<br><br>8 weeks	1 (140 patients)	MD: -1.59 (95% CI: -3 to -0.18)<br><br>Quick Inventory of Depressive Symptomatology-16-item (QIDS-SR-16) (Range: 0 to 27; higher scores indicate greater impairment)		Moderate<br><br>due to serious imprecision[a]	Probably little or no important effect on depression.
Quality of life<br><br>8 weeks	1 (140 patients)	MD: 2.36 (95% CI: 0.71 to 4.01)<br><br>World Health Organisation-5 Well-Being Index (WHO-5) (Range: 0 to 25; higher scores indicate less impairment)		Moderate<br><br>due to serious imprecision[a]	Probably little or no important effect on quality of life.
Cognitive function<br><br>8 weeks	1 (141 patients)	MD: -0.02 (95% CI: -0.24 to 0.2)<br><br>Digital Symbol Substitution Test (Higher scores indicate less impairment)		High	Little or no important effect on cognitive function.

RR: Relative risk; RD: Risk difference; MD: Mean difference; CI: Confidence interval

a The confidence interval includes both appreciable benefit and no important effect.

McIntyre RS, Phan L, Kwan ATH, Mansur RB, Rosenblat JD, Guo Z, et al. Vortioxetine for the treatment of post-COVID-19 condition: a randomized controlled trial. Brain. 2024;147(3):849-57.

Supplement 11: Summary of findings table comparing Leronlimab and Placebo

Patients: Patients with long COVID
Intervention: Leronlimab
Comparator: Placebo

Outcome	Trials (patients)	Relative Risk (95% CI)	Absolute effect (per 1000 patients)	Certainty of the evidence	Plain language summary
Fatigue<br><br>8 weeks	1 (55 patients)	MD: -0.08 (95% CI: -0.65 to 0.49)<br><br>Ad-hoc symptom severity score (Range: 0 to 3; higher scores indicate greater impairment)		Low<br><br>due to very serious imprecision[a]	May have little or no important effect on fatigue.
Mental health<br><br>8 weeks	1 (55 patients)	MD: 0.03 (95% CI: -0.45 to 0.51)<br><br>Ad-hoc symptom severity score (Range: 0 to 3; higher scores indicate greater impairment)		Low<br><br>due to very serious imprecision[a]	May have little or no important effect on mental health.
Cognitive function<br><br>8 weeks	1 (55 patients)	MD: 0.08 (95% CI: -0.45 to 0.61)<br><br>Ad-hoc symptom severity score (Range: 0 to 3; higher scores indicate greater impairment)		Low<br><br>due to very serious imprecision[a]	May have little or no important effect on cognitive function.
Dyspnea<br><br>8 weeks	1 (55 patients)	MD: -0.23 (95% CI: -0.75 to 0.29)<br><br>Ad-hoc symptom severity score (Range: 0 to 3; higher scores indicate greater impairment)		Low<br><br>due to very serious imprecision[a]	May have little or no important effect on dyspnea.
Post-exertional malaise<br><br>8 weeks	1 (55 patients)	MD: -0.11 (95% CI: -0.66 to 0.44)<br><br>Ad-hoc symptom severity score (Range: 0 to 3; higher scores indicate greater impairment)		Low<br><br>due to very serious imprecision[a]	May have little or no important effect post-exertional malaise.

RR: Relative risk; RD: Risk difference; MD: Mean difference; CI: Confidence interval

a Likely much too few participants to achieve prognostic balance.

Gaylis NB, Ritter A, Kelly SA, Pourhassan NZ, Tiwary M, Sacha JB, et al. Reduced Cell Surface Levels of C-C Chemokine Receptor 5 and Immunosuppression in Long Coronavirus Disease 2019 Syndrome. Clin Infect Dis. 2022;75(7):1232-4.

Supplement 12: Summary of findings table comparing Glucosaminyl muramyl dipeptide ('Licopid') and Usual care

Patients: Patients with long COVID
Intervention: Glucosaminyl muramyl dipeptide ('Licopid')
Comparator: Usual care

Outcome	Trials (patients)	Relative Risk (95% CI)	Absolute effect (per 1000 patients)	Certainty of the evidence	Plain language summary
Physical function<br><br>8 weeks	1 (60 patients)	MD: 1.82 (95% CI: -2.68 to 6.32)<br><br>SF-36 Physical component score (Range: 0 to 100; higher scores indicate less impairment)		Very low<br><br>due to serious risk of bias[a] and very serious imprecision[b]	Uncertain of the effect on physical function.
Physical function<br><br>24 weeks	1 (60 patients)	MD: 6.88 (95% CI: 2.92 to 10.84)<br><br>SF-36 Physical component score (Range: 0 to 100; higher scores indicate less impairment)		Very low<br><br>due to serious risk of bias[a] and very serious imprecision[b]	Uncertain of the effect on physical function.
Physical function<br><br>8 weeks	1 (60 patients)	MD: 1 (95% CI: -4.14 to 6.14)<br><br>SF-36 physical functioning subscale (Range: 0 to 100; higher scores indicate less impairment)		Very low<br><br>due to serious risk of bias[a] and very serious imprecision[b]	Uncertain of the effect on physical function.
Physical function<br><br>24 weeks	1 (60 patients)	MD: 4.67 (95% CI: 0.13 to 9.2)<br><br>SF-36 physical functioning subscale (Range: 0 to 100; higher scores indicate less impairment)		Very low<br><br>due to serious risk of bias[a] and very serious imprecision[b]	Uncertain of the effect on physical function.
Mental health<br><br>8 weeks	1 (60 patients)	MD: -1.51 (95% CI: -3.41 to 0.39)<br><br>Hospital Anxiety and Depression Scale (HADS) anxiety subscale (Range: 0 to 21; higher scores indicate greater impairment)		Very low<br><br>due to serious risk of bias[a] and very serious imprecision[b]	Uncertain of the effect on anxiety.
Mental health<br><br>24 weeks	1 (60 patients)	MD: -2.47 (95% CI: -4.52 to -0.42)<br><br>Hospital Anxiety and Depression Scale (HADS) anxiety subscale (Range: 0 to 21; higher scores indicate greater impairment)		Very low<br><br>due to serious risk of bias[a] and very serious imprecision[b]	Uncertain of the effect on anxiety.

Mental health<br><br>8 weeks	1 (60 patients)	MD: -1.6 (95% CI: -3.73 to 0.53)<br><br>Hospital Anxiety and Depression Scale (HADS) depression subscale (Range: 0 to 21; higher scores indicate greater impairment)	Very low<br><br>due to serious risk of bias[a] and very serious imprecision[b]	Uncertain of the effect on depression.
Mental health<br><br>24 weeks	1 (60 patients)	MD: -1.57 (95% CI: -3.41 to 0.27)<br><br>Hospital Anxiety and Depression Scale (HADS) depression subscale (Range: 0 to 21; higher scores indicate greater impairment)	Very low<br><br>due to serious risk of bias[a] and very serious imprecision[b]	Uncertain of the effect on depression.
Mental health<br><br>8 weeks	1 (60 patients)	MD: 5.13 (95% CI: -0.19 to 10.46)<br><br>SF-36 Mental component score (Range: 0 to 100; higher scores indicate less impairment)	Very low<br><br>due to serious risk of bias[a] and very serious imprecision[b]	Uncertain of the effect on mental health.
Mental health<br><br>24 weeks	1 (60 patients)	MD: 7.73 (95% CI: 2.61 to 12.85)<br><br>SF-36 Mental component score (Range: 0 to 100; higher scores indicate less impairment)	Very low<br><br>due to serious risk of bias[a] and very serious imprecision[b]	Uncertain of the effect on mental health.
Mental health<br><br>8 weeks	1 (60 patients)	MD: 1.5 (95% CI: -4.68 to 7.68)<br><br>SF-36 mental health subscale (Range: 0 to 100; higher scores indicate less impairment)	Very low<br><br>due to serious risk of bias[a] and very serious imprecision[b]	Uncertain of the effect on mental health.
Mental health<br><br>24 weeks	1 (60 patients)	MD: 5 (95% CI: 1.45 to 8.55)<br><br>SF-36 mental health subscale (Range: 0 to 100; higher scores indicate less impairment)	Very low<br><br>due to serious risk of bias[a] and very serious imprecision[b]	Uncertain of the effect on mental health.
Pain<br><br>8 weeks	1 (60 patients)	MD: -2 (95% CI: -4.4 to 0.4)<br><br>SF-36 bodily pain subscale (Range: 0 to 100; higher scores indicate less impairment)	Very low<br><br>due to serious risk of bias[a] and very serious imprecision[b]	Uncertain of the effect on pain.
Pain<br><br>24 weeks	1 (60 patients)	MD: -1 (95% CI: -2.78 to 0.78)<br><br>SF-36 bodily pain subscale (Range: 0 to 100; higher scores indicate less impairment)	Very low<br><br>due to serious risk of bias[a] and very serious imprecision[b]	Uncertain of the effect on pain.

RR: Relative risk; RD: Risk difference; MD: Mean difference; CI: Confidence interval
a Concerns related to bias due to the randomization process, deviations from the intended intervention, and measurement of outcome.
b Likely much too few participants to achieve prognostic balance.
Sizyakina LP, Zakurskaya VY, Guryanova SV. Glucosaminyl muramyl dipeptide efficacy in post-COVID-19 patient rehabilitation treatment. Infectious diseases: News, Opinions, Training. 2023;12(1):17-25.

Supplement 13: Summary of findings table comparing Actovegin and Usual care

<table>
<tr><td colspan="2">Patients: Patients with long COVID</td><td colspan="4" rowspan="3">NOTE: CONCERNS WITH TRIAL INTEGRITY. The trial reported recruiting 444 patients with long COVID being treated at a single in-patient neurology unit in Russia—what we considered to be a very large number of patients with long COVID at a single center. The trial reports an equal number of participants randomized to each arm though it does not describe using block randomization. The p-values reported for baseline characteristics do not appear to correspond to the figures reported. Baseline characteristics across arms appear inconceivably similar to each other to an extent that may be improbable with randomization. The trial also reports implausibly large improvement in cognitive function according to the (MoCA). The trial is unregistered.</td></tr>
<tr><td colspan="2">Intervention: Actovegin</td></tr>
<tr><td colspan="2">Comparator: Usual care</td></tr>
<tr><th>Outcome</th><th>Trials (patients)</th><th>Relative Risk (95% CI)</th><th>Absolute effect (per 1000 patients)</th><th>Certainty of the evidence</th><th>Plain language summary</th></tr>
<tr><td>Fatigue

8.57 weeks</td><td>1 (444 patients)</td><td colspan="2">MD: -12.8 (95% CI: -15.36 to -10.24)

Multidimensional Fatigue Inventory-20 (MFI-20) (Range: 20 to 100; higher scores indicate greater impairment)</td><td>Moderate

due to serious risk of bias[a]</td><td>Probably improves fatigue.</td></tr>
<tr><td>Cognitive function

8.57 weeks</td><td>1 (444 patients)</td><td colspan="2">MD: 3 (95% CI: 2.02 to 3.98)

Montreal Cognitive Assessment test (MoCA) (Range: 0 to 30; higher scores indicate less impairment)</td><td>Moderate

due to serious risk of bias[a]</td><td>Probably improves cognitive function.</td></tr>
<tr><td colspan="6">RR: Relative risk; RD: Risk difference; MD: Mean difference; CI: Confidence interval</td></tr>
<tr><td colspan="6">a Concerns related to bias due to the randomization process, deviations from the intended intervention, and measurement of outcome.</td></tr>
<tr><td colspan="6">Kutashov VA. Actovegin use in patients with cognitive impairment after coronavirus infection (COVID-19). Neurology, Neuropsychiatry, Psychosomatics. 2021;13(2):65-72.</td></tr>
</table>

Supplement 14: Summary of findings table comparing Physiotherapy, Multicomponent exercise of progressively increasing intensity and Physiotherapy

Patients: Patients with long COVID
Intervention: Physiotherapy, Multicomponent exercise of progressively increasing intensity
Comparator: Physiotherapy

Outcome	Trials (patients)	Relative Risk (95% CI)	Absolute effect (per 1000 patients)	Certainty of the evidence	Plain language summary
Physical function<br>12.85 weeks	1 (60 patients)	MD: 6.96 (95% CI: 2.7 to 11.22)<br>SF-36 Physical Component Score (Range: 0 to 100; higher scores indicate less impairment)		Low<br>due to very serious imprecision[a]	May improve physical function.
Mental health<br>12.85 weeks	1 (60 patients)	MD: 2.06 (95% CI: -3.52 to 7.64)<br>SF-36 Mental Component Score (Range: 0 to 100; higher scores indicate less impairment)		Low<br>due to very serious imprecision[a]	May have little or no important effect on mental health.
Dyspnea<br>12.85 weeks	1 (60 patients)	MD: -0.76 (95% CI: -1.2 to -0.32)<br>Modified Medical Research Council Dyspnea Scale (Range: 0 to 4; higher scores indicate greater impairment)		Low<br>due to very serious imprecision[a]	May improve dyspnea.
Dyspnea<br>12.85 weeks	1 (60 patients)	MD: -18.61 (95% CI: -27.4 to -9.82)<br>Multidimensional Dyspnoea Profile (Range: 0 to 10; higher scores indicate greater impairment)		Low<br>due to very serious imprecision[a]	May improve dyspnea.
Dyspnea<br>12.85 weeks	1 (60 patients)	MD: -1.74 (95% CI: -2.79 to -0.69)<br>Multidimensional Dyspnoea Profile - Breathing Discomfort (Range: 0 to 10; higher scores indicate greater impairment)		Low<br>due to very serious imprecision[a]	May improve breathing discomfort.
Dyspnea<br>12.85 weeks	1 (60 patients)	MD: -6.95 (95% CI: -12.44 to -1.46)<br>Multidimensional Dyspnoea Profile - Emotional Response (Range: 0 to 10; higher scores indicate greater impairment)		Low<br>due to very serious imprecision[a]	May improve emotional response to dyspnea.
Dyspnea<br>12.85 weeks	1 (60 patients)	MD: -9.93 (95% CI: -14.56 to -5.3)<br>Multidimensional Dyspnoea Profile - Sensory Dimension		Low<br>due to very serious imprecision[a]	May improve dyspnea.

		(Range: 0 to 10; higher scores indicate greater impairment)		
RR: Relative risk; RD: Risk difference; MD: Mean difference; CI: Confidence interval				
a Likely much too few participants to achieve prognostic balance.				
Romanet C, Wormser J, Fels A, Lucas P, Prudat C, Sacco E, et al. Effectiveness of exercise training on the dyspnoea of individuals with long COVID: A randomised controlled multicentre trial. Ann Phys Rehabil Med. 2023;66(5):101765.				

Supplement 15: Summary of findings table comparing Intermittent aerobic exercise and Continuous aerobic exercise

Patients: Patients with long COVID
Intervention: Intermittent aerobic exercise
Comparator: Continuous aerobic exercise

Outcome	Trials (patients)	Relative Risk (95% CI)	Absolute effect (per 1000 patients)	Certainty of the evidence	Plain language summary
Physical function<br><br>5 weeks	1 (110 patients)	MD: 3.8 (95% CI: 1.12 to 6.48)<br><br>SF-36 Physical component score (Range: 0 to 100; higher scores indicate less impairment)		Moderate<br><br>due to serious risk of bias[a]	Probably improves physical function.
Mental health<br><br>5 weeks	1 (110 patients)	MD: 0 (95% CI: -3.69 to 3.69)<br><br>SF-36 Mental component score (Range: 0 to 100; higher scores indicate less impairment)		Low<br><br>due to serious risk of bias[a] and serious imprecision[b]	May have little or no important effect on mental health.

RR: Relative risk; RD: Risk difference; MD: Mean difference; CI: Confidence interval

a Concerns related to bias due to the randomization process and missing data.

b The confidence interval include both appreciable benefit and harm.

Mooren JM, Garbsch R, Schafer H, Kotewitsch M, Waranski M, Teschler M, et al. Medical Rehabilitation of Patients with Post-COVID-19 Syndrome-A Comparison of Aerobic Interval and Continuous Training. J Clin Med. 2023;12(21).

Supplement 16: Summary of findings table comparing Low-intensity aerobic exercise, Strength training and High-intensity aerobic exercise, Strength training

**Patients:** Men with long COVID and sarcopenia<br>**Intervention:** Low-intensity aerobic training, Strength training<br>**Comparator:** High-intensity aerobic training, Strength training		**NOTE:** CONCERNS WITH TRIAL INTEGRITY. The trial is registered in March 2021. The trial, however, was conducted between March 2020 to April 2021. The standard deviations of baseline characteristics and outcome measures are remarkably small.			
**Outcome**	**Trials (patients)**	**Relative Risk (95% CI)**	**Absolute effect (per 1000 patients)**	**Certainty of the evidence**	**Plain language summary**
Quality of life<br><br>8 weeks	1 (73 patients)	MD: 8.5 (95% CI: 8.08 to 8.92)<br><br>Sarcopenia and Quality of Life (SarQol) (Range: 0 to 100; higher scores indicate less impairment)		Low<br><br>due to serious indirectness[a] and serious imprecision[b]	May improve quality of life.
Quality of life<br><br>24 weeks	1 (69 patients)	MD: 10.4 (95% CI: 9.97 to 10.83)<br><br>Sarcopenia and Quality of Life (SarQol) (Range: 0 to 100; higher scores indicate less impairment)		Low<br><br>due to serious indirectness[a] and serious imprecision[b]	May improve quality of life.
RR: Relative risk; RD: Risk difference; MD: Mean difference; CI: Confidence interval					
a The trial was conducted in men with sarcopenia.					
b Likely too few participants to achieve prognostic balance.					
Nambi GA-O, Abdelbasset WA-OX, Alrawaili SM, Elsayed SH, Verma A, Vellaiyan A, et al. Comparative effectiveness study of low versus high-intensity aerobic training with resistance training in community-dwelling older men with post-COVID 19 sarcopenia: A randomized controlled trial. 2021(1477-0873 (Electronic))					

Supplement 17: Summary of findings table comparing Inspiratory muscle training and Usual care

**Patients:** Patients with long COVID<br>**Intervention:** Inspiratory muscle training<br>**Comparator:** Usual care					
**Outcome**	**Trials (patients)**	**Relative Risk (95% CI)**	**Absolute effect (per 1000 patients)**	**Certainty of the evidence**	**Plain language summary**
Quality of life<br><br>8 weeks	1 (148 patients)	MD: -1.3 (95% CI: -5.9 to 3.3)<br><br>King's Brief Interstitial Lung Disease - Total score (Range: 0 to 100; higher scores indicate less impairment)		Low<br><br>due to serious risk of bias[a] and serious imprecision[b]	May have little or no important effect on quality of life.
Dyspnea<br><br>8 weeks	1 (148 patients)	MD: 2.4 (95% CI: -2.66 to 7.46)<br><br>King's Brief Interstitial Lung Disease - Breathlessness and activities domain (Range: 0 to 100; higher scores indicate less impairment)		Low<br><br>due to serious risk of bias[a] and serious imprecision[c]	May have little or no important effect on breathlessness and activities.
Dyspnea<br><br>8 weeks	1 (148 patients)	MD: 1.1 (95% CI: 0.44 to 1.76)<br><br>Transition Dyspnea Index (TDI) (Range: -9 to 9; higher scores indicate less impairment)		Low<br><br>due to serious risk of bias[a] and serious imprecision[c]	May improve dyspnea.
RR: Relative risk; RD: Risk difference; MD: Mean difference; CI: Confidence interval					
a Concerns related to bias due to deviations from the intended intervention, missing outcome data, and measurement of outcome.					
b The confidence interval includes both appreciable benefit and harm.					
c The confidence interval includes both appreciable benefit and no important effect.					
McNarry MA, Berg RMG, Shelley J, Hudson J, Saynor ZL, Duckers J, et al. Inspiratory muscle training enhances recovery post-COVID-19: a randomised controlled trial. Eur Respir J. 2022;60(4).					

Supplement 18: Summary of findings table comparing Active cycle of breathing technique, Physiotherapy and Physiotherapy

**Patients:** Patients with long COVID<br>**Intervention:** Active cycle of breathing technique, Physiotherapy<br>**Comparator:** Physiotherapy		**NOTE:** CONCERNS WITH TRIAL INTEGRITY. The trial reports minimal baseline characteristics that appear remarkably similar across trial arms with little variability and 0 patients lost to follow-up. Equal numbers of participants are randomized to each trial arm though the trial report does not describe block randomization. The trial reports even, round numbers (e.g., age of participants is reported to range between 40 and 50 years). The p value reported corresponding to the difference in age between arms at baseline appears incorrect. The trial is unregistered.			
**Outcome**	**Trials (patients)**	**Relative Risk (95% CI)**	**Absolute effect (per 1000 patients)**	**Certainty of the evidence**	**Plain language summary**
Fatigue<br><br>12 weeks	1 (60 patients)	MD: -9.97 (95% CI: -11.17 to -8.77)<br><br>Fatigue Assessment Scale-10 (Range: 10 to 50; higher scores indicate greater impairment)		Very Low<br><br>due to serious risk of bias[a] and very serious imprecision[b]	Uncertain of the effect on fatigue.

RR: Relative risk; RD: Risk difference; MD: Mean difference; CI: Confidence interval

a Concerns related to bias due to the randomization process and deviations from the intended intervention.

b Likely much too few participants to achieve prognostic balance.

Alshaimaa A. Ali NGE, Samir A. Algazzar , Abdel Wahab M. Lotfy , Emad M. Taha. Impact Of Active Cycle Of Breathing Technique On Selected Pulmonary Outcomes In Post-COVID Syndrome Patients. Journal of Pharmaceutical Negative Results. 2023:710-7.

Supplement 19: Summary of findings table comparing In-patient rehabilitation, Physiotherapy, Acupuncture and In-patient rehabilitation, Physiotherapy

**Patients:** Patients with long COVID		**NOTE:** CONCERNS WITH TRIAL INTEGRITY. The trial reports an equal number of participants randomized to each arm though the trial does not describe block randomization. The trial reports implausibly small measures of variability for outcome measures. The trial is unregistered.			
**Intervention:** In-patient rehabilitation, Physiotherapy, Acupuncture					
**Comparator:** In-patient rehabilitation, Physiotherapy					
**Outcome**	**Trials (patients)**	**Relative Risk (95% CI)**	**Absolute effect (per 1000 patients)**	**Certainty of the evidence**	**Plain language summary**
Dyspnea<br><br>1.7 weeks	1 (160 patients)	MD: -0.6 (95% CI: -0.74 to -0.46)<br><br>Modified Medical Research Council dyspnea scale (mMRC dyspnea scale) (Range: 0 to 4; higher scores indicate greater impairment)		Low<br><br>due to serious risk of bias[a] and serious indirectness[b]	May improve dyspnea.
RR: Relative risk; RD: Risk difference; MD: Mean difference; CI: Confidence interval					
a Concerns related to bias due to deviations from the intended intervention and the measurement of outcome.					
b In-patient setting.					
Omarova I, Akanova A, Kurmanova A, Kurmanova G, Glushkova N, Seidanova A, et al. Acupuncture as an Additional Method of Rehabilitation Post-COVID-19: a randomized controlled trial. J Pharmacopuncture. 2023;26(3):238-46.					

Supplement 20: Summary of findings table comparing an Online cognitive behavioral therapy (CBT) program called 'Fit after Covid' and Usual care

Patients: Patients with long COVID
Intervention: Cognitive behavioral therapy (CBT)
Comparator: Usual care

Outcome	Trials (patients)	Relative Risk (95% CI)	Absolute effect (per 1000 patients)	Certainty of the evidence	Plain language summary
Recovery/improvement[a]<br><br>19 weeks	1 (113 patients)	RR: 2.24 (95% CI: 1.38 to 3.64)	326 more (95% CI: 100 more to 694 more)	Low<br><br>due to serious risk of bias[b] and serious imprecision[c]	May increase the proportion of patients who experience important recovery/improvement.
Recovery/improvement[a]<br><br>24 weeks	1 (108 patients)	RR: 2.43 (95% CI: 1.48 to 3.98)	371 more (95% CI: 124 more to 773 more)	Low<br><br>due to serious risk of bias[b] and serious imprecision[c]	May increase the proportion of patients who experience important recovery/improvement.
Fatigue<br><br>19 weeks	1 (114 patients)	MD: -9.3 (95% CI: -13.18 to -5.42)<br><br>Checklist Individual Strength (CIS) fatigue subscale (Range: 8 to 56; higher scores indicate greater impairment)		Moderate<br><br>due to serious risk of bias[b]	Probably improves fatigue.
Fatigue<br><br>24 weeks	1 (114 patients)	MD: -8.4 (95% CI:-13.11 to -3.69)<br><br>Checklist Individual Strength (CIS) fatigue subscale (Range: 8 to 56; higher scores indicate greater impairment)		Moderate<br><br>due to serious risk of bias[b]	Probably improves fatigue.
Physical function<br><br>19 weeks	1 (114 patients)	MD: 9.4 (95% CI: 4.41 to 14.39)<br><br>SF-36 Physical function subscale (Range: 0 to 100; higher scores indicate less impairment)		Moderate<br><br>due to serious risk of bias[b]	Probably improves physical function.
Physical function<br><br>24 weeks	1 (114 patients)	MD: 4.9 (95% CI: -1.89 to 11.69)<br><br>SF-36 Physical function subscale (Range: 0 to 100; higher scores indicate less impairment)		Low<br><br>due to serious risk of bias[b] and serious imprecision[d]	May have little or no important effect on physical function.

Cognitive function<br><br>19 weeks	1 (114 patients)	MD: -5.2 (95% CI: -7.14 to -3.26)<br><br>Checklist Individual Strength (CIS) concentration problems subscale (Range: 5 to 35; higher scores indicate greater impairment)		Moderate<br><br>due to serious risk of bias[b]	Probably improves concentration.
Cognitive function<br><br>24 weeks	1 (114 patients)	MD: -5.2 (95% CI: -7.97 to -2.43)<br><br>Checklist Individual Strength (CIS) concentration problems subscale (Range: 5 to 35; higher scores indicate greater impairment)		Moderate<br><br>due to serious risk of bias[b]	Probably improves concentration.
Serious adverse events<br><br>24 weeks	1 (114 patients)	RD: 0% (95% CI: -3% to 3%)	0 more (95% CI: 30 fewer to 30 more)	Very low<br><br>due to serious risk of bias[b] and very serious imprecision[e]	Uncertain of the effect on SAE.
RR: Relative risk; RD: Risk difference; MD: Mean difference; CI: Confidence interval					
a Defined as no longer fatigued (score <35) according to the Checklist Individual Strength (CIS-fatigue).					
b Concerns related to bias due to deviations from the intended intervention and measurement of outcome.					
c Few observed events make results fragile.					
d The confidence interval includes both appreciable benefit and no important effect.					
e Confidence interval includes no important effect and appreciable harm. Only one event observed in the trial.					
Kuut TA, Muller F, Csorba I, Braamse A, Aldenkamp A, Appelman B, et al. Efficacy of Cognitive-Behavioral Therapy Targeting Severe Fatigue Following Coronavirus Disease 2019: Results of a Randomized Controlled Trial. Clin Infect Dis. 2023;77(5):687-95.					

Supplement 21: Summary of findings table comparing a Mobile application providing education on long COVID ('telerehabilitation mobile app') and Usual care

**Patients:** Patients with long COVID<br>**Intervention:** Telerehabilitation app<br>**Comparator:** Usual care					
**Outcome**	**Trials (patients)**	**Relative Risk (95% CI)**	**Absolute effect (per 1000 patients)**	**Certainty of the evidence**	**Plain language summary**
Physical function<br><br>24 weeks	1 (87 patients)	MD: -3.46 (95% CI: -9.07 to 2.15)<br><br>SF-36 Physical component score (Range: 0 to 100; higher scores indicate less impairment)		Low<br><br>due to serious risk of bias[a] and serious imprecision[b]	May have little or no important effect on physical function.
Mental health<br><br>24 weeks	1 (87 patients)	MD: 1.87 (95% CI: -5.39 to 9.13)<br><br>SF-36 Mental component score (Range: 0 to 100; higher scores indicate less impairment)		Low<br><br>due to serious risk of bias[a] and serious imprecision[b]	May have little or no important effect on mental health.
Mental health<br><br>24 weeks	1 (87 patients)	MD: -0.12 (95% CI: -2.51 to 2.27)<br><br>Hospital Anxiety and Depression Scale (HADS) (Range: 0 to 21; higher scores indicate greater impairment)		Low<br><br>due to serious risk of bias[a] and serious imprecision[b]	May have little or no important effect on mental health.
Cognitive function<br><br>24 weeks	1 (87 patients)	MD: 0.61 (95% CI: -0.9 to 2.12)<br><br>Montreal Cognitive Assessment test (MoCA) (Range: 0 to 30; higher scores indicate less impairment)		Low<br><br>due to serious risk of bias[a] and serious imprecision[b]	May have little or no important effect on cognitive function.
Serious adverse events<br><br>24 weeks	1 (100 patients)	RD: 0% (95% CI: -4% to 4%)	0 more (95% CI: 40 fewer to 40 more)	Very low<br><br>due to serious risk of bias[a] and very serious imprecision[c]	Uncertain of the effect on SAE.
RR: Relative risk; RD: Risk difference; MD: Mean difference; CI: Confidence interval					
a Concerns related to bias due to the randomization process, deviations from the intended intervention, and measurement of outcome.					
b Likely too few participants to achieve prognostic balance.					
c Confidence interval includes no important effect and appreciable harm. No events reported in trial.					
Samper-Pardo M, Leon-Herrera S, Olivan-Blazquez B, Mendez-Lopez F, Dominguez-Garcia M, Sanchez-Recio R. Effectiveness of a telerehabilitation intervention using ReCOVery APP of long COVID patients: a randomized, 3-month follow-up clinical trial. Sci Rep. 2023;13(1):7943.					

Supplement 22: Summary of findings table comparing Amygdala and insula retraining and Education related to self-management

Patients: Patients with long COVID
Intervention: Amygdala and insula retraining
Comparator: Education related to self-management

Outcome	Trials (patients)	Relative Risk (95% CI)	Absolute effect (per 1000 patients)	Certainty of the evidence	Plain language summary
Fatigue<br><br>12 weeks	1 (42 patients)	MD: -1.48 (95% CI: -3 to 0.04)<br><br>Multidimensional Fatigue Inventory-20 (MFI-20) general fatigue subscale (Range: 20 to 100; higher scores indicate greater impairment)		Very Low<br><br>due to serious risk of bias[a] and very serious imprecision[b]	Uncertain of the effect on fatigue.

RR: Relative risk; RD: Risk difference; MD: Mean difference; CI: Confidence interval

a Concerns related to bias due to the randomization process and missing outcome data.

b Likely much too few participants to achieve prognostic balance.

Toussaint LL, Bratty AJ. Amygdala and Insula Retraining (AIR) Significantly Reduces Fatigue and Increases Energy in People with Long COVID. Evid Based Complement Alternat Med. 2023;2023:7068326.

Supplement 23: Summary of findings table comparing a formulation of Probiotics and prebiotics ('Synbiotics') called SIM01 and Placebo

Patients: Patients with long COVID
Intervention: A formulation of probiotics and prebiotics (SIM01)
Comparator: Placebo

Outcome	Trials (patients)	Relative Risk (95% CI)	Absolute effect (per 1000 patients)	Certainty of the evidence	Plain language summary
Recovery/improvement[a]<br><br>24 weeks	1 (398 patients)	RR: 1.47 (95% CI: 1.22 to 1.79)	200 more (95% CI: 94 more to 336 more)	Low<br><br>due to serious risk of bias[d]<br>due to other concerns [e]	May increase the proportion of patients who experience important recovery/improvement.
Recovery/improvement[b]<br><br>24 weeks	1 (323 patients)	RR: 1.62 (95% CI: 1.29 to 2.04)	239 more (95% CI: 112 more to 401 more)	Low<br><br>due to serious risk of bias[d]<br>due to other concerns [e]	May increase the proportion of patients who experience important recovery/improvement.
Recovery/improvement[c]<br><br>24 weeks	1 (285 patients)	RR: 1.28 (95% CI: 1.05 to 1.54)	150 more (95% CI: 27 more to 290 more)	Low<br><br>due to serious risk of bias[d]<br>due to other concerns [e]	May increase the proportion of patients who experience important recovery/improvement.
Quality of life<br><br>24 weeks	1 (403 patients)	MD: 1.5 (95% CI: -0.87 to 3.87)<br><br>Visual analogue scale (VAS) (0-100) (Range: 0 to 100; higher scores indicate less impairment)		Moderate<br><br>due to other concerns [e]	Probably has little or no important effect on quality of life.
Serious adverse events<br><br>24 weeks	1 (463 patients)	RD: 0% (95% CI: -1% to 1%)	0 more (95% CI: 10 fewer to 10 more)	Low<br><br>due to very serious imprecision[f]	May have little or no important effect on SAE.

RR: Relative risk; RD: Risk difference; MD: Mean difference; CI: Confidence interval

a Alleviation of fatigue symptoms was defined as reduction in the severity of symptoms leading to improvement in activities of daily living using PACSQ-14 questionnaire.

b Alleviation of difficulty in concentration was defined as reduction in the severity of symptoms leading to improvement in activities of daily living using PACSQ-14 questionnaire.
c Alleviation of shortness of breath was defined as reduction in the severity of symptoms leading to improvement in activities of daily living using PACSQ-14 questionnaire.
d Concerns related to bias due to selection of reported results.
e While the trial reports a large effect for alleviation of fatigue, concentration, and dyspnea, there is no plausible mechanism of action for these effects, particularly for concentration and dyspnea. Further, this formulation of synbiotics, SIM01, has not been independently tested and shown to be effective for other conditions except by its innovators and patent holders.
f No events reported in trial.
Lau RI, Su Q, Lau ISF, Ching JYL, Wong MCS, Lau LHS, et al. A synbiotic preparation (SIM01) for post-acute COVID-19 syndrome in Hong Kong (RECOVERY): a randomised, double-blind, placebo-controlled trial. Lancet Infect Dis. 2024;24(3):256-65.

Supplement 24: Summary of findings table comparing Coenzyme Q10 and Placebo

Patients: Patients with long COVID
Intervention: Coenzyme Q10
Comparator: Placebo

Outcome	Trials (patients)	Relative Risk (95% CI)	Absolute effect (per 1000 patients)	Certainty of the evidence	Plain language summary
Quality of life<br><br>6 weeks	1 (119 patients)	MD: -0.04 (95% CI: -0.1 to 0.02)<br><br>EQ-5D health index (Range: 0 to 1; higher scores indicate less impairment)		Moderate<br><br>due to serious imprecision[a]	Probably has little or no important effect on quality of life.
Serious adverse events<br><br>6 weeks	1 (119 patients)	RD: 0% (95% CI: -3% to 3%)	0 more (95% CI: 30 fewer to 30 more)	Low<br><br>due to very serious imprecision[b]	May have little or no important effect on SAE.

RR: Relative risk; RD: Risk difference; MD: Mean difference; CI: Confidence interval

a The confidence interval include both appreciable harm and benefit.

b The confidence interval includes no important effect and appreciable harm. No events reported in trial.

Hansen KS, Mogensen TH, Agergaard J, Schiøttz-Christensen B, Østergaard L, Vibholm LK, et al. High-dose coenzyme Q10 therapy versus placebo in patients with post COVID-19 condition: a randomized, phase 2, crossover trial. Lancet Reg Health Eur. 2023;24:100539.

Supplement 25: Summary of findings table comparing a combination of L-arginine, vitamin C and Placebo

Patients: Patients with long COVID
Intervention: L-arginine, vitamin C
Comparator: Placebo

Outcome	Trials (patients)	Relative Risk (95% CI)	Absolute effect (per 1000 patients)	Certainty of the evidence	Plain language summary
Recovery/Improvement[a]<br><br>4 weeks	1 (46 patients)	RR: 10.5 (95% CI: 2.78 to 39.71)	826 more (95% CI: 155 more to 3366 more)	Very Low<br><br>due to serious risk of bias[b] and very serious imprecision[d]	Uncertain of the effect on important recovery/improvement.
Serious adverse events<br><br>4 weeks	1 (46 patients)	RD: 0% (95% CI: -0.08% to 0.08%)	0 more (95% CI: 80 fewer to 80 more)	Very Low<br><br>due to serious risk of bias[c] and very serious imprecision[e]	Uncertain of the effect on SAE.

RR: Relative risk; RD: Risk difference; MD: Mean difference; CI: Confidence interval

a Absence of fatigue. Fatigue was operationalized as the response "most or all the time" to item seven of the Center for Epidemiological Studies Depression Scale (CES-D, "I felt that everything I did was an effort").

b Concerns related to bias due to the randomization process and deviations from the intended intervention.

c Concerns related to bias due to the randomization process, deviations from the intended intervention, and measurement of outcome.

d Likely much too few participants to achieve prognostic balance and low event rate makes results fragile.

e Likely much too few participants to achieve prognostic balance and no events observed in the trial.

Tosato M, Calvani R, Picca A, Ciciarello F, Galluzzo V, Coelho-Junior HJ, et al. Effects of l-Arginine Plus Vitamin C Supplementation on Physical Performance, Endothelial Function, and Persistent Fatigue in Adults with Long COVID: A Single-Blind Randomized Controlled Trial. Nutrients. 2022;14(23).

Supplement 26: Summary of findings table comparing Brainmax and Placebo

**Patients:** Patients with long COVID<br>**Intervention:** Trimethylhydrazinium propionate and ethylmethylhydroxypyridine succinate ('Brainmax')<br>**Comparator:** Placebo		**NOTE:** CONCERNS WITH TRIAL INTEGRITY. The trial is registered retrospectively. Equal numbers of participants are randomized to each group though the trial does not describe block randomization. The trial does not report any baseline characteristics. The trial also reported an inconceivably large effect on the MoCA.			
**Outcome**	**Trials (patients)**	**Relative Risk (95% CI)**	**Absolute effect (per 1000 patients)**	**Certainty of the evidence**	**Plain language summary**
Recovery/improvement[a]<br><br>5.85 weeks	1 (159 patients)	RR: 4.27 (95% CI: 2.68 to 7.14)	597 more (95% CI: 298 to 1088)	High	Increases the proportion of patients who experience important recovery/improvement.
Fatigue<br><br>5.85 weeks	1 (159 patients)	MD: -3.33 (95% CI: -5.56 to -1.11)<br><br>Fatigue Assessment Scale-10 (FAS-10) (Range: 10 to 50; higher scores indicate greater impairment)		High	Improves fatigue.
Fatigue<br><br>5.85 weeks	1 (159 patients)	MD: -15.5 (95% CI: -18.33 to -12.67)<br><br>Multidimensional Fatigue Inventory-20 (MFI-20) (Range: 20 to 100; higher scores indicate greater impairment)		High	Improves fatigue.
Mental health<br><br>5.85 weeks	1 (159 patients)	MD: -1.12 (95% CI: -5.94 to 3.71)<br><br>Beck Anxiety Inventory (Range: 0 to 63; higher scores indicate greater impairment)		Moderate<br><br>due to serious imprecision[b]	Probably has little or no important effect on anxiety.
Cognitive function<br><br>5.85 weeks	1 (159 patients)	MD: -3.67 (95% CI: -5.46 to -1.87)<br><br>Montreal Cognitive Assessment test (MoCA) (Range: 0 to 30; higher scores indicate less impairment)		High	Reduces cognitive function.
Serious adverse events<br><br>5.85 weeks	1 (159 patients)	RD: -0.01% (95% CI: -0.05% to 0.02%)	10 fewer (50 fewer to 20 more)	Low	May have little or no important effect on SAE.

				due to very serious imprecision[c]	
RR: Relative risk; RD: Risk difference; MD: Mean difference; CI: Confidence interval					
a Recovery without consequences.					
b The confidence interval includes both appreciable benefit and no important effect.					
c The confidence interval includes no important effect and appreciable harm. Only one event observed in the trial.					
Tanashyan MM, Raskurazhev AA, Kuznetsova PI, Bely PA, Zaslavskaya KI. [Prospects and possibilities for the treatment of patients with long COVID-19 syndrome]. Ter Arkh. 2022;94(11):1285-93.					

Supplement 27: Summary of findings table comparing Hyperbaric oxygen therapy and Placebo

Patients: Patients with long COVID
Intervention: Hyperbaric oxygen therapy
Comparator: Placebo

Outcome	Trials (patients)	Relative Risk (95% CI)	Absolute effect (per 1000 patients)	Certainty of the evidence	Plain language summary
Physical function<br><br>10 weeks	1 (73 patients)	MD: -5.2 (95% CI: -14.06 to 3.66)<br><br>SF-36 Physical function subscale (Range: 0 to 100; higher scores indicate less impairment)		Low<br><br>due to very serious imprecision[a]	May have little or no important effect on physical function.
Mental health<br><br>10 weeks	1 (73 patients)	MD: -7.1 (95% CI: -12.23 to -1.97)<br><br>Brief Symptom Inventory-18 (BSI-18) (Range: 0 to 72; higher scores indicate greater impairment)		Low<br><br>due to very serious imprecision[a]	May improve mental health.
Mental health<br><br>10 weeks	1 (73 patients)	MD: -2 (95% CI: -4.26 to 0.26)<br><br>Brief Symptom Inventory-18 (BSI-18) Anxiety Subscale (Range: 0 to 24; higher scores indicate greater impairment)		Low<br><br>due to very serious imprecision[a]	May have little or no important effect on anxiety.
Mental health<br><br>10 weeks	1 (73 patients)	MD: -2.4 (95% CI: -4.66 to -0.14)<br><br>Brief Symptom Inventory-18 (BSI-18) Depression Subscale (Range: 0 to 24; higher scores indicate greater impairment)		Low<br><br>due to very serious imprecision[a]	May have little or no important effect on depression.
Mental health<br><br>10 weeks	1 (73 patients)	MD: -2.6 (95% CI: -4.64 to -0.56)<br><br>Brief Symptom Inventory-18 (BSI-18) Somatization Subscale (Range: 0 to 24; higher scores indicate greater impairment)		Low<br><br>due to very serious imprecision[a]	May have little or no important effect on mental health.
Mental health<br><br>10 weeks	1 (73 patients)	MD: 10 (95% CI: -0.01 to 20.01)		Low	May improve mental health.

		SF-36 Mental Health Subscale (Range: 0 to 100; higher scores indicate less impairment)	due to very serious imprecision[a]	
Cognitive function<br><br>10 weeks	1 (73 patients)	MD: 3.4 (95% CI: 0.3 to 6.5)<br><br>NeuroTrax Computerized Cognitive Testing Battery - Global Score (Mean: 100, SD: 15; higher scores indicate less impairment)	Low<br><br>due to very serious imprecision[a]	May have little or no important effect on cognitive function.
Pain<br><br>10 weeks	1 (73 patients)	MD: -0.1 (95% CI: -1.05 to 0.85)<br><br>Brief Pain Inventory (BPI) Pain Severity Subscale (Range: 0 to 10; higher scores indicate greater impairment)	Low<br><br>due to very serious imprecision[a]	May have little or no important effect on pain.
Pain<br><br>10 weeks	1 (73 patients)	MD: 7 (95% CI: -5.78 to 19.78)<br><br>SF-36 Bodily Pain Subscale (Range: 0 to 100; higher scores indicate less impairment)	Low<br><br>due to very serious imprecision[a]	May have little or no important effect on pain.
RR: Relative risk; RD: Risk difference; MD: Mean difference; CI: Confidence interval				
a Likely much too few participants to achieve prognostic balance.				
Zilberman-Itskovich S, Catalogna M, Sasson E, Elman-Shina K, Hadanny A, Lang E, et al. Hyperbaric oxygen therapy improves neurocognitive functions and symptoms of post-COVID condition: randomized controlled trial. Sci Rep. 2022;12(1):11252.				

Supplement 28: Summary of findings table comparing Transcranial direct current stimulation, Physiotherapy, Education related to activities of daily living and Physiotherapy, Education related to self-management

Patients: Patients with long COVID
Intervention: Transcranial direct current stimulation, Physiotherapy, Education related to activities of daily living
Comparator: Physiotherapy, Education related to self-management

Outcome	Trials (patients)	Relative Risk (95% CI)	Absolute effect (per 1000 patients)	Certainty of the evidence	Plain language summary
Recovery/improvement[a]<br><br>5 weeks	1 (70 patients)	RR: 1.69 (95% CI: 1.13 to 2.53)	315 more (95% CI: 59 more to 699 more)	Low<br><br>due to very serious imprecision[b]	May increase the proportion of patients who experience important recovery/improvement.
Fatigue<br><br>5 weeks	1 (70 patients)	MD: -12.4 (95% CI: -17.33 to -7.47)<br><br>Modified Fatigue Impact Scale (MFIS) (Range: 0 to 84; higher scores indicate greater impairment)		Low<br><br>due to very serious imprecision[c]	May improve fatigue.
Fatigue<br><br>5 weeks	1 (70 patients)	MD: -9.32 (95% CI: -13.14 to -5.5)<br><br>Modified Fatigue Impact Scale (MFIS)- Cognitive subscale (Range: 0 to 40; higher scores indicate greater impairment)		Low<br><br>due to very serious imprecision[c]	May improve cognitive fatigue.
Fatigue<br><br>5 weeks	1 (70 patients)	MD: -0.71 (95% CI: -4.77 to 3.35)<br><br>Modified Fatigue Impact Scale (MFIS)- Physical subscale (Range: 0 to 36; higher scores indicate greater impairment)		Low<br><br>due to very serious imprecision[c]	May have little or no important effect on physical fatigue.
Mental health<br><br>5 weeks	1 (70 patients)	MD: -4.91 (95% CI: -7.5 to -2.32)<br><br>Hamilton Anxiety Rating Scale (HAM-A) (Range: 0 to 56; higher scores indicate greater impairment)		Low<br><br>due to very serious imprecision[c]	May improve anxiety.
Pain<br><br>5 weeks	1 (70 patients)	MD: -1.03 (95% CI: -3.84 to 1.78)<br><br>McGill Pain Questionnaire (Range: 0 to 50; higher scores indicate greater impairment)		Low<br><br>due to very serious imprecision[c]	May have little or no important effect on pain.
Quality of life<br><br>5 weeks	1 (70 patients)	MD: 14.8 (95% CI: 8.86 to 20.74)<br><br>World Health Organization quality of life questionnaire		Low	May improve quality of life.

		(brief version) (Range: 0 to 100; higher scores indicate less impairment)		due to very serious imprecision[c]	
Serious adverse events<br><br>5 weeks	1 (70 patients)	RD: 0% (95% CI: -5% to 5%)	0 more (95% CI: 50 fewer to 50 more)	Very low<br><br>due to extremely serious imprecision[d]	Uncertain of the effect on SAE.
RR: Relative risk; RD: Risk difference; MD: Mean difference; CI: Confidence interval					
a 5-point reduction of the baseline MFIS score.					
b Likely much too few participants to achieve prognostic balance and low event rate makes results fragile.					
c Likely much too few participants to achieve prognostic balance.					
d Likely much too few participants to achieve prognostic balance. Confidence interval includes no important effect and appreciable harm. No events reported in trial.					
Santana K, Franca E, Sato J, Silva A, Queiroz M, de Farias J, et al. Non-invasive brain stimulation for fatigue in post-acute sequelae of SARS-CoV-2 (PASC). Brain Stimul. 2023;16(1):100-7.					

Supplement 29: Summary of findings table comparing Photobiomodulation and Placebo

Patients: Patients with long COVID<br>Intervention: Photobiomodulation<br>Comparator: Placebo		**NOTE:** CONCERNS WITH TRIAL INTEGRITY. The trial reports few baseline characteristics, and reports 0 participants lost to follow-up. The trial reports even, round numbers (e.g., age of participants is reported to range between 60 and 70 and BMI between 30 and 35). There is exceptionally little variance in outcome measures. The clinical trial registration cited in the trial report also describes an entirely different trial than the one reported. The authors have a history of retractions due to concerns with research integrity.			
**Outcome**	**Trials (patients)**	**Relative Risk (95% CI)**	**Absolute effect (per 1000 patients)**	**Certainty of the evidence**	**Plain language summary**
Fatigue<br><br>4 weeks	1 (100 patients)	MD: -0.28 (95% CI: -0.38 to -0.18)<br><br>Fatigue Severity Scale (FSS) (Range: 1 to 7; higher scores indicate greater impairment)		Moderate<br><br>due to serious risk of bias[a]	Probably little or no important effect on fatigue.
Physical function<br><br>4 weeks	1 (100 patients)	MD: 0.32 (95% CI: 0 to 0.64)<br><br>Katz Index of Independence in Tasks of Everyday Living (Range: 0 to 6; higher scores indicate less impairment)		Low<br><br>due to serious risk of bias[a] and serious imprecision[b]	May have little or no important effect on physical function.
RR: Relative risk; RD: Risk difference; MD: Mean difference; CI: Confidence interval					
a Concerns related to bias due to the randomization process.					
b The confidence interval include both appreciable benefit and no important effect.					
Elbannaa R, Mogahed H, Zahran M, Mohamed E. The effect of photobiomodulation versus placebo on functional capacity and fatigability in post COVID-19 elderly. Advances in Rehabilitation. 2022;36(3):19-25.					

Supplement 30: Summary of findings table comparing Physical and mental health rehabilitation and Usual care

Patients: Patients with long COVID
Intervention: Physical and mental health rehabilitation
Comparator: Usual care

Outcome	Trials (patients)	Results	Absolute effect (per 1000 patients)	Certainty of the evidence	Plain language summary
Recovery/Improvement[a]<br><br>12 weeks	1 (487 patients)	RR: 1.58 (95% CI: 1.27 to 1.97)	186 more (95% CI: 86 more to 310 more)	Moderate[b]<br><br>due to serious risk of bias[c]	Probably increases the proportion of patients who experience important recovery/improvement.
Recovery/Improvement[a]<br><br>52 weeks	1 (442 patients)	RR: 1.55 (95% CI: 1.21 to 2)	161 more (95% CI: 61 more to 292 more)	Moderate[b]<br><br>due to serious risk of bias[c]	Probably increases the proportion of patients who experience important recovery/improvement.
Fatigue<br><br>12 weeks	1 (485 patients)	MD: -2.42 (95% CI: -3.82 to -1.02)<br><br>PROMIS (patient-reported outcomes measurement information system)- Fatigue subscore (Mean: 50, SD: 10; higher scores indicate greater impairment)		Low[b]<br><br>due to serious risk of bias[c] and serious imprecision[d]	May have little or no important effect on fatigue.
Fatigue<br><br>52 weeks	1 (440 patients)	MD: -2 (95% CI: -3.96 to -0.04)<br><br>PROMIS (patient-reported outcomes measurement information system)- Fatigue subscore (Mean: 50, SD: 10; higher scores indicate greater impairment)		Low[b]<br><br>due to serious risk of bias[c] and serious imprecision[d]	May have little or no important effect on fatigue.
Physical function<br><br>12 weeks	1 (486 patients)	MD: 0.59 (95% CI: -0.29 to 1.47)<br><br>PROMIS (patient-reported outcomes measurement information system)- Physical function abilities subscore (Mean: 50, SD: 10; higher scores indicate greater impairment)		Moderate[b]<br><br>due to serious risk of bias[c]	Probably little or no important effect on physical function.
Physical function<br><br>52 weeks	1 (441 patients)	MD: 0.5 (95% CI: -1.01 to 2.01)<br><br>PROMIS (patient-reported outcomes measurement information system)- Physical function abilities subscore (Mean: 50, SD: 10; higher scores indicate greater impairment)		Moderate[b]<br><br>due to serious risk of bias[c]	Probably little or no important effect on physical function.

Mental health<br><br>12 weeks	1 (426 patients)	MD: -0.6 (95% CI: -1.51 to 0.31)<br><br>Hospital Anxiety and Depression Scale (HADS) anxiety subscale (Range: 0 to 21; higher scores indicate greater impairment)	Low[b]<br><br>due to serious risk of bias[c] and serious imprecision[d]	May have little or no important effect on anxiety.
Mental health<br><br>52 weeks	1 (423 patients)	MD: -1 (95% CI: -1.98 to -0.02)<br><br>Hospital Anxiety and Depression Scale (HADS) anxiety subscale (Range: 0 to 21; higher scores indicate greater impairment)	Low[b]<br><br>due to serious risk of bias[c] and serious imprecision[d]	May have little or no important effect on anxiety.
Mental health<br><br>12 weeks	1 (422 patients)	MD: -0.7 (95% CI: -1.59 to 0.19)<br><br>Hospital Anxiety and Depression Scale (HADS) depression subscale (Range: 0 to 21; higher scores indicate greater impairment)	Low[b]<br><br>due to serious risk of bias[c] and serious imprecision[d]	May have little or no important effect on depression.
Mental health<br><br>52 weeks	1 (417 patients)	MD: -1.5 (95% CI: -2.41 to -0.59)<br><br>Hospital Anxiety and Depression Scale (HADS) depression subscale (Range: 0 to 21; higher scores indicate greater impairment)	Moderate[b]<br><br>due to serious risk of bias[c]	Probably improves depression.
Mental health<br><br>12 weeks	1 (486 patients)	MD: -0.96 (95% CI: -2.33 to 0.41)<br><br>PROMIS (patient-reported outcomes measurement information system)- Emotional distress – Anxiety subscore (Mean: 50, SD: 10; higher scores indicate greater impairment)	Moderate[b]<br><br>due to serious risk of bias[c]	Probably little or no important effect on anxiety.
Mental health<br><br>52 weeks	1 (441 patients)	MD: -1.8 (95% CI: -3.77 to 0.17)<br><br>PROMIS (patient-reported outcomes measurement information system)- Emotional distress – Anxiety subscore (Mean: 50, SD: 10; higher scores indicate greater impairment)	Low[b]<br><br>due to serious risk of bias[c] and serious imprecision[d]	May have little or no important effect on anxiety.
Mental health<br><br>12 weeks	1 (485 patients)	MD: -1.16 (95% CI: -2.49 to 0.17)<br><br>PROMIS (patient-reported outcomes measurement information system)- Emotional distress – Depression subscore (Mean: 50, SD: 10; higher scores indicate greater impairment)	Moderate[b]<br><br>due to serious risk of bias[c]	Probably little or no important effect on depression.

Mental health<br><br>52 weeks	1 (440 patients)	MD: -2.2 (95% CI: -4.16 to -0.24)<br><br>PROMIS (patient-reported outcomes measurement information system)- Emotional distress – Depression subscore (Mean: 50, SD: 10; higher scores indicate greater impairment)	Low[b]<br><br>due to serious risk of bias[c] and serious imprecision[d]	May have little or no important effect on depression.
Quality of life<br><br>12 weeks	1 (482 patients)	MD: 0.02 (95% CI: -0.03 to 0.07)<br><br>EQ5D-5L Index Score (Range: 0 to 100; higher scores indicate less impairment)	Low[b]<br><br>due to serious risk of bias[c] and serious imprecision[e]	May have little or no important effect on quality of life.
Quality of life<br><br>52 weeks	1 (438 patients)	MD: 0.04 (95% CI: -0.01 to 0.09)<br><br>EQ5D-5L Index Score (Range: 0 to 100; higher scores indicate less impairment)	Low[b]<br><br>due to serious risk of bias[c] and serious imprecision[d]	May improve quality of life.
Quality of life<br><br>12 weeks	1 (481 patients)	MD: 4.7 (95% CI: 1.06 to 8.34)<br><br>EQ5D-5L Visual Analogue Scale (0-100 cm) (Range: 0 to 100; higher scores indicate less impairment)	Low[b]<br><br>due to serious risk of bias[c] and serious imprecision[d]	May have little or no important effect on quality of life.
Quality of life<br><br>52 weeks	1 (438 patients)	MD: 5.4 (95% CI: 1.29 to 9.51)<br><br>EQ5D-5L Visual Analogue Scale (0-100 cm) (Range: 0 to 100; higher scores indicate less impairment)	Low[b]<br><br>due to serious risk of bias[c] and serious imprecision[d]	May have little or no important effect on quality of life.
Quality of life<br><br>12 weeks	1 (485 patients)	MD: 0.03 (95% CI: 0.01 to 0.05)<br><br>PROMIS 29+2 Profile v2.1 (PROPr) (HRQoL) (Range: -0.022 to 1; higher scores indicate less impairment)	Low[b]<br><br>due to serious risk of bias[c] and serious imprecision[d]	May have little or no important effect on quality of life.
Quality of life<br><br>52 weeks	1 (444 patients)	MD: 0.04 (95% CI: 0 to 0.08)<br><br>PROMIS 29+2 Profile v2.1 (PROPr) (HRQoL) (Range: -0.022 to 1; higher scores indicate less impairment)	Moderate[b]<br><br>due to serious risk of bias[c]	Probably improves quality of life.
Cognitive function<br><br>12 weeks	1 (444 patients)	MD: -0.2 (95% CI:-1.94 to 1.54)<br><br>Cognitive Function (PROMIS Neuro-QoL) (Mean: 50, SD: 10; higher scores indicate less impairment)	Moderate[b]<br><br>due to serious risk of bias[c]	Probably little or no important effect on cognitive function.

Cognitive function<br><br>52 weeks	1 (427 patients)	MD: 1.1 (95% CI: -0.76 to 2.96)<br><br>Cognitive Function (PROMIS Neuro-QoL) (Mean: 50, SD: 10; higher scores indicate less impairment)	Moderate[b]<br><br>due to serious risk of bias[c]	Probably little or no important effect on cognitive function.
Cognitive function<br><br>12 weeks	1 (485 patients)	MD: 0.13 (95% CI: -1.1 to 1.36)<br><br>PROMIS (patient-reported outcomes measurement information system)- Cognitive function abilities subscore (Mean: 50, SD: 10; higher scores indicate less impairment)	Moderate[b]<br><br>due to serious risk of bias[c]	Probably little or no important effect on cognitive function.
Cognitive function<br><br>52 weeks	1 (440 patients)	MD: 1 (95% CI: -0.44 to 2.44)<br><br>PROMIS (patient-reported outcomes measurement information system)- Cognitive function abilities subscore (Mean: 50, SD: 10; higher scores indicate less impairment)	Moderate[b]<br><br>due to serious risk of bias[c]	Probably little or no important effect on cognitive function.
Dyspnea<br><br>12 weeks	1 (443 patients)	MD: -0.9 (95% CI: -2.59 to 0.79)<br><br>PROMIS (patient-reported outcomes measurement information system) Dyspnoea Severity Short Form (Mean: 50, SD: 10; higher scores indicate greater impairment)	Moderate[b]<br><br>due to serious risk of bias[c]	Probably little or no important effect on dyspnea.
Dyspnea<br><br>52 weeks	1 (425 patients)	MD: -0.5 (95% CI: -2.37 to 1.37)<br><br>PROMIS (patient-reported outcomes measurement information system) Dyspnoea Severity Short Form (Mean: 50, SD: 10; higher scores indicate greater impairment)	Moderate[b]<br><br>due to serious risk of bias[c]	Probably little or no important effect on dyspnea.
Pain<br><br>12 weeks	1 (484 patients)	MD: -0.29 (95% CI: -0.66 to 0.08)<br><br>PROMIS (patient-reported outcomes measurement information system)- Pain intensity subscore (Mean: 50, SD: 10; higher scores indicate greater impairment)	Moderate[b]<br><br>due to serious risk of bias[c]	Probably little or no important effect on pain.
Pain<br><br>52 weeks	1 (440 patients)	MD: -0.2 (95% CI: -0.7 to 0.3)<br><br>PROMIS (patient-reported outcomes measurement information system)- Pain intensity subscore (Mean: 50, SD: 10; higher scores indicate greater impairment)	Moderate[b]<br><br>due to serious risk of bias[c]	Probably little or no important effect on pain.

Serious adverse events<br><br>52 weeks	1 (585 patients)	RD: 0.02% (-0.01% to 0.05%)	20 more (95% CI: 10 fewer to 50 more)	Very low[b]<br><br>due to serious risk of bias[c] and very serious imprecision[f]	Uncertain of the effect on SAE.
RR: Relative risk; RD: Risk difference; MD: Mean difference; CI: Confidence interval					
a Overall health compared to three months ago described as "much better now" or "somewhat better now".					
b All patients experienced severe COVID-19 infection, requiring hospitalization. We opted to not rate down the certainty of evidence for indirectness because there is no evidence that currently suggests the effects of the intervention may be different based on severity of the acute COVID-19 infection.					
c Concerns related to bias due to deviations from the intended intervention, missing outcome data, and measurement of outcome.					
d The confidence interval include both appreciable benefit and no important effect.					
e The confidence interval include both appreciable benefit and appreciable harm.					
f Confidence interval includes no important effect and appreciable harm. Few events observed in the trial.					
McGregor G, Sandhu H, Bruce J, Sheehan B, McWilliams D, Yeung J, et al. Clinical effectiveness of an online supervised group physical and mental health rehabilitation programme for adults with post-covid-19 condition (REGAIN study): multicentre randomised controlled trial. BMJ. 2024;384:e076506.					
